# NtrBC Regulates Invasiveness and Virulence of *Pseudomonas aeruginosa* During High-Density Infection

**DOI:** 10.3389/fmicb.2020.00773

**Published:** 2020-05-05

**Authors:** Morgan A. Alford, Arjun Baghela, Amy T. Y. Yeung, Daniel Pletzer, Robert E. W. Hancock

**Affiliations:** ^1^Centre for Microbial Diseases and Immunity Research, University of British Columbia, Vancouver, BC, Canada; ^2^Wellcome Sanger Institute, Hinxton, United Kingdom; ^3^Department of Microbiology, University of Otago, Dunedin, New Zealand

**Keywords:** abscess, adaptive lifestyles, high-density infection, invasiveness, nitrogen metabolism, NtrC, *Pseudomonas aeruginosa*, virulence

## Abstract

*Pseudomonas aeruginosa* is an opportunistic pathogen that is a major cause of nosocomial and chronic infections contributing to morbidity and mortality in cystic fibrosis patients. One of the reasons for its success as a pathogen is its ability to adapt to a broad range of circumstances. Here, we show the involvement of the general nitrogen regulator NtrBC, which is structurally conserved but functionally diverse across species, in pathogenic and adaptive states of *P. aeruginosa*. The role of NtrB and NtrC was examined in progressive or chronic infections, which revealed that mutants (Δ*ntrB*, Δ*ntrC*, and Δ*ntrBC*) were reduced in their ability to invade and cause damage in a high-density abscess model *in vivo.* Progressive infections were established with mutants in the highly virulent PA14 genetic background, whereas chronic infections were established with mutants in the less virulent clinical isolate LESB58 genetic background. Characterization of adaptive lifestyles *in vitro* confirmed that the double Δ*ntrBC* mutant demonstrated >40% inhibition of biofilm formation, a nearly complete inhibition of swarming motility, and a modest decrease and altered surfing motility colony appearance; with the exception of swarming, single mutants generally had more subtle or no changes. Transcriptional profiles of deletion mutants under swarming conditions were defined using RNA-Seq and unveiled dysregulated expression of hundreds of genes implicated in virulence in PA14 and LESB58 chronic lung infections, as well as carbon and nitrogen metabolism. Thus, transcriptional profiles were validated by testing responsiveness of mutants to several key intermediates of central metabolic pathways. These results indicate that NtrBC is a global regulatory system involved in both pathological and physiological processes relevant to the success of *Pseudomonas* in high-density infection.

## Introduction

Nitrogen is an essential element of life that is critical for the normal synthesis of proteins and nucleic acids constituting 3% of the human body by mass (Rohmer et al., 2011). Pools of inorganic and organic nitrogen are found in the soil, water and atmosphere (Johnson et al., 2010). Despite their abundance, environmental forms of nitrogen are largely inaccessible to plants and animals and must be transformed for biological use. Transformation of nitrogen into its many oxidation states is dependent on microbial, especially bacterial, activity and can occur through nitrification, denitrification or nitrogen fixation among other processes (Vicente and Dean, 2017). *Pseudomonas aeruginosa* is a ubiquitous bacterium that thrives in a wide range of environments, within and outside the host, and is an important symbiont that provides fixed nitrogen to plants through physical association in the rhizosphere (Arat et al., 2015).

*P. aeruginosa* is known for its ability to adapt to many environmental circumstances, which is reflected by numerous regulatory networks essential for sensing and responding appropriately to stimuli (Galan-Vasquez et al., 2011). The rapid response to environmental changes is often mediated by signaling through two-component systems, which are often encoded as a sensor kinase and cognate response regulator under the control of a single promoter, and these systems can be activated by the binding of a particular stimulant to the sensor kinase (Rodrigue et al., 2000; Francis et al., 2018).

NtrBC is a two-component system that is structurally conserved but functionally distinct across bacterial classes (Luque-Almagro et al., 2011; Bhagirath et al., 2019). Nonetheless, it has been described as responsive to intracellular glutamine levels and is important for general nitrogen regulation and nitrate assimilation (Luque-Almagro et al., 2011; Bhagirath et al., 2019). Molecular characterization of NtrBC activity in *P. aeruginosa* is limited and most studies of primary nitrogen metabolism have been performed in distinct species such as *Escherichia coli* (Leigh and Dodsworth, 2007; Hervas et al., 2009). Upon stimulation, the sensor kinase NtrB autophosphorylates and a phosphate group is transferred to its cognate response regulator NtrC for activation (Wang and Gralla, 1996; Hervas et al., 2009). NtrC mediates the expression of genes implicated in numerous other physiological processes, in part by increasing the binding affinity of the alternative sigma factor RpoN/σ^54^, best recognized for its transcriptional regulation of genes involved in bacterial stress responses, to the RNA polymerase (Boor, 2006; Brown et al., 2014).

In contrast to its potentially beneficial role in the rhizosphere, *P. aeruginosa* is listed among the most threatening opportunistic human pathogens for which new antibiotics are urgently needed (Yeung et al., 2012; Klein et al., 2016). It is well known for causing chronic, eventually-fatal lung infections in patients with cystic fibrosis, and is a major cause of nosocomial lung infections, as well as being associated with chronic obstructive pulmonary disorder and local infections. In each of these instances, *P. aeruginosa* infection can form a biofilm, which is highly resistant to antibiotic therapy and immune clearance and is influenced by nitrate sensing and metabolism (Van Alst et al., 2007). *P. aeruginosa* can form biofilms or exhibit rapid surface motility to cope with environmental circumstances (Fuente-Nunez et al., 2013, 2014). Strain PA14 is a highly virulent laboratory strain of *P. aeruginosa* that displays these phenotypes more readily that the less motile, clinical isolate LESB58 (Pletzer et al., 2017, 2020).

Swimming organisms benefit by migrating toward certain optimal nutrient sources and migrating away from unfavorable growth circumstances, enabling them to colonize new environments including host tissues distal to the site of infection (Harshey, 2003; Rendueles and Velicer, 2016). Motility also enables migration to locations where more persistent lifestyles can be adopted by forming surface-associated biofilms (Haiko and Westerlund-Wikstrom, 2013; Rendueles and Velicer, 2016; Sun et al., 2018). Adaptive surface-associated motility, in the form of swarming and surfing in *P. aeruginosa*, is thought to enable the spread of bacteria on surfaces of the body, such as in the lungs. Consistent with these roles, adaptive motility is intrinsically associated with bacterial metabolism and often coupled with the expression of virulence factors (Rajagopala et al., 2007; Haiko and Westerlund-Wikstrom, 2013). Phenotypic screening of transposon mutants revealed that NtrC contributes to swarming motility (Yeung et al., 2009). Furthermore, we previously showed that mutants in *ntrB* and *ntrC* demonstrated modestly (∼20%) increased toxicity and substantially reduced adherence to epithelial cells (Gellatly et al., 2018). Here, the pathogenic properties of NtrB and NtrC were further explored. The role of NtrBC *in vivo* was examined in an abscess model of high-density infection (Pletzer et al., 2017) and shown to be involved in full invasiveness (PA14) and virulence (LESB58). It was shown that deletions in the genes encoding these regulators in the highly virulent PA14 strain significantly reduced or completely inhibited swarming motility as well as biofilm formation, in a medium dependent fashion. These data were explained by the dysregulated expression of hundreds of genes in Ntr deletion mutants that, taken with our phenotypic data, suggests a global role for NtrBC as a regulator of adaptive resistance and virulence that has not been well appreciated previously.

## Materials and Methods

### Bacterial Strains, and Growth Conditions

Bacterial strains and plasmids used in this study are described in Table 1. Overnight cultures were routinely maintained in Luria-Bertani (LB) broth. Overnight and sub-cultures were incubated for no longer than 18 h at 37°C while shaking (250 rpm). Modified forms of basal medium (BM2) consisting of 62 mM potassium phosphate buffer (pH = 7.0), 7 mM (NH_4_)_2_SO_4_, 2 mM MgSO_4_, 10 μM FeSO_4_ supplemented with various sources of carbon: 20 mM glucose, 20 mM citrate, 35 mM succinate or 35 mM malate were used for swarming assays with 0.1% [wt/vol] casamino acids (CAA) replacing (NH_4_)_2_SO_4_ since ammonium inhibits swarming. For testing the influence of nitrogen source on growth and swarming, (NH_4_)_2_SO_4_ was replaced by equimolar concentrations of NaNO_3_, NaNO_2_, urea or glutamate and 20 mM glucose was used as the carbon source. Other media used in assays are described elsewhere. *E. coli* strains were routinely cultured in double yeast tryptone (dYT) at 37°C while shaking (250 rpm). *E. coli* XL-1 Blue was used as the cloning host and ST-18 for biparental mating where the medium was supplemented with 100 μg/ml 5-aminolevulinic acid (ALA). For plasmid selection in *E. coli* donor strains, 12.5 μg/ml gentamicin (Gm) was added to growth media. For plasmid selection in *P. aeruginosa* parent strains PA14 and LESB58, 50 μg/ml and 500 μg/ml Gm was added to growth media. Bacterial growth was monitored by measuring optical density (OD_600_) with a spectrophotometer (Eppendorf, Missisauga, ON, Canada).

**TABLE 1:** Bacterial strains and plasmids used in this study.

**Strain or plasmid**	**Relevant characteristics^a^**	**References**
***Escherichia coli***		
XL-1 Blue	*recA1 endA1 gyrA96 thi-* 1 *hsdR17* (rK- mK+) *supE44 relA1 lac* [*F’ proAB laclq ZΔM15Tn10*(Tc^r^)]	Stratagene
ST-18	*pro thi hsdR^+^* Tp^r^ Sm^r^; chromosome::RP4-2 Tc::Mu-Km::Tn7/λ*pir ΔhemA*	Thoma and Schobert, 2009
***Pseudomonas aeruginosa***		
PA14	WT *P. aeruginosa* UBCPP-PA14	Rahme et al., 1995
PA14 Δ*ntrB*	PA14 *ntrB* chromosomal deletion	This study
PA14 Δ*ntrC*	PA14 *ntrC* chromosomal deletion	This study
PA14 Δ*ntrBC*	PA14 *ntrBC* chromosomal deletion	This study
LESB58	WT *P. aeruginosa* Liverpool Epidemic Strain B58	Cheng et al., 1996
LESB58 Δ*ntrB*	LESB58 *ntrB* chromosomal deletion	This study
LESB58 Δ*ntrC*	LESB58 *ntrC* chromosomal deletion	This study
LESB58 Δ*ntrBC*	LESB58 *ntrBC* chromosomal deletion	This study
**Plasmids**		
pEX18Gm	Gene replacement vector, suicide plasmid carrying *sacB*, Gm^r^	Hoang et al., 1998
pEX18Gm.Δ*ntrB*	Cloned 0.94 kbp fusion fragment flanking *ntrB*, Gm^r^	This study
pEX18Gm.Δ*ntrC*	Cloned 1.01 kbp fusion fragment flanking *ntrC*, Gm^r^	This study
pEX18Gm.Δ*ntrBC*	Cloned 2.48 kbp fusion fragment flanking *ntrBC*, Gm^r^	This study
pBBR1MCS-5	Broad host-range cloning vector, Gm^r^	Kovach et al., 1994
pBBR-5.*ntrB*	Cloned 1.08 kbp *ntrB* gene, Gm^r^	This study
pBBR-5.*ntrC*	Cloned 1.44 kbp *ntrC* gene, Gm^r^	This study
pBBR-5.*ntrBC*	Cloned 2.51 kbp *ntrBC* gene, Gm^r^	This study

*^*a*^Antibiotics: gentamicin (Gm), tetracycline (Tc), trimethoprim (Tp), streptomycin (Sm).*

### General DNA Manipulations

Primers used in polymerase chain reaction (PCR) assays are listed in Supplementary Table S1. High-fidelity PCR was carried out using the Phusion DNA Polymerase (Thermo Fisher Scientific) in accordance with the manufacturer’s specifications and optimized annealing temperatures. Oligomer sequences were based on the genome of *P. aeruginosa* UBCPP-PA14 (GenBank: NC_008463.1) or LESB58 (GenBank: NC_002516.2) available from NCBI, referred to as PA14 and LESB58, respectively, in this manuscript. For PCR reactions performed with PA14 or LESB58, cells were boiled at 98°C with shaking (1,000 rpm) for 10 min and pelleted by centrifugation at 14,500 rpm for 3 min.

Restriction digests were performed using FastDigest restriction enzymes according to the manufacturer’s specifications (Thermo Fisher Scientific). All ligation reactions were carried out at room temperature using T4 DNA ligase (Invitrogen). DNA purifications were performed using the GeneJET PCR purification kit or the GeneJET Gel extraction kit following the manufacturer’s instructions (Thermo Scientific).

### Recombinant DNA Manipulations

Construction of the knockout vectors was based on the protocol described by Pletzer et al. (2014). Briefly, 500 bp regions flanking the 5′ and 3′ ends of PA14 and LESB58 *ntrB*, *ntrC*, and *ntrBC* coding regions were PCR-amplified using respective primer pairs (Supplementary Table S1). Since the nucleotide sequences for the regions of interest were 100% identical, one set of primers were sufficient for amplification from the chromosome of either strain. Reverse-complement sequences were added to primers to provide homology between flanking regions for continuous amplification in overlap-extension PCR. After each round of amplification, fragments were gel purified. The fusion product was ligated into the pEX18Gm vector and verified by sequencing (Eurofins, Toronto, ON, Canada).

Chromosomal deletions in PA14 and LESB58 (Δ*ntrB*, Δ*ntrC*, and Δ*ntrBC*, respectively) were obtained by conjugational transfer of the gene replacement vector into the appropriate parent strain. *E. coli* ST-18 was made electrocompetent by washing with 10% [vol/vol] glycerol on ice (4°C). Gene replacement vectors were introduced by electroporation (1.8 kV). Bacteria were scratched from the surface of an agar plate, resuspended in one ml of sterile water and adjusted to an OD_600_ = 0.1. 100 μl of *E. coli* ST-18 was mixed with 200 μl *P. aeruginosa* PA14 or LESB58 and spotted onto dYT agar plates supplemented with 100 μg/ml ALA for overnight growth. On the next day, spots were scratched from the surface of the plate, resuspended in one ml sterile water and diluted 1,000-fold. Hundred microliter of dilute suspension was spread on LB agar plates with appropriate antibiotic. On the next day, single colonies were picked on LB agar plates containing 10% [wt/vol] sucrose for counter-selection of mutants from single-crossovers. Gene deletion was confirmed by PCR and sequencing (Eurofins, Toronto, ON, Canada).

### Construction of Complementation Plasmids

Construction of complementation vectors was based on the protocol of Kovach et al. (1994). Briefly, the coding region of PA14 and LESB58 *ntrB*, *ntrC*, and *ntrBC* was PCR amplified using appropriate complementation primer pairs (Supplementary Table S1). PCR products were gel purified and digested with restriction enzymes *EcoRI* and *BamHI*. PCR products were subsequently cloned in *EcoRI*/*BamHI*-digested pBBR1MCS-5 (pBBR-5). *P. aeruginosa* PA14 or LESB58 were made electrocompetent by washing with 300 mM sucrose at room temperature (20°C) and plasmids introduced by electroporating (2.5 kV). Successful transformant were selected by picking on LB agar plates with the appropriate antibiotic and confirmed by plasmid isolation.

### Study Approval and Animals

Animal experiments were performed in accordance with the Canadian Council on Animal Care (CCAC) guidelines and were approved by the University of British Columbia Animal Care Committee protocol (A14-0253). Mice used in this study were outbred CD-1 mice (female). All animals were purchased from Charles River Laboratories, Inc. (Wilmington, MA, United States) and were 7–8 weeks of age at the time of experiments. Mice weighed 25 ± 2 g.

### Cutaneous (Abscess) Infection Model

We tested invasiveness of PA14 WT and mutants in infection and virulence of LESB58 WT and mutants in chronic infection using a nuanced subcutaneous abscess model as previously described (Pletzer et al., 2017). All strains were sub-cultured at 37°C with shaking (250 rpm) to an OD_600_ = 1.0 in LB. Cells were washed twice with sterile phosphate buffered saline (PBS) and resuspended to a final OD_600_ = 0.5 or 1.0 for PA14 or LESB58 strains, respectively. Both strains were used to form high-density abscess infections (inoculated with 5 ± 3 × 10^7^ CFU and containing > 10^8^ CFU at the experimental endpoint) to model invasive or chronic infections depending on the strain used (Pletzer and Hancock, 2018). Abscesses were formed by injection of 50 μl of bacteria on the left dorsum of mice for 24 or 72 h for invasive or chronic infection. When appropriate, disease progression was monitored daily. Abscess lesion size or visible dermonecrosis was measured using a caliper. Abscesses and/or organs distal to the site of infection (including the heart, liver, lungs, kidneys, and spleen) were harvested in PBS and homogenized using a Mini-Beadbeater (BioSpec Products, Bartlesville, OK, United States) for bacterial enumeration on LB. Three independent experiments containing three or four biological replicates each were performed.

### Biofilm Formation

We examined PA14 WT and mutants for biofilm formation using a high-throughput microtiter assay as described elsewhere (Fuente-Nunez et al., 2013). Overnight cultures were diluted to a starting OD_600_ = 0.1 in BM2 swarming medium with 20 mM glucose and added to polypropylene 96-well plates (Falcon). Following 18-24 h static incubation at 37°C, biomass was stained with 0.1% [wt/vol] CV and dissolved in 70% [vol/vol] ethanol. OD_595_ was read using a BioTek SynergyH1 microplate reader (BioTek, Winooski, VT, United States). Three independent experiments containing three biological replicates each were performed.

### Growth Curves

We investigated if weak growth in the presence of glucose as the sole carbon source was influenced by nitrogen source in BM2 supplemented with various aforementioned compounds and compared growth of PA14 *ntrBC* mutants to WT in these conditions (see bacterial growth subsection). We further investigated if we could improve growth by providing glucose in excess or substituting equimolar amounts of TCA cycle intermediates and, again, compared growth of PA14 *ntrBC* mutants to WT. PA14 strains were grown overnight in BM2 supplemented with ammonium sulfate, casamino acids, sodium nitrate or sodium nitrite as the nitrogen source. Growth of PA14 strains adjusted to a starting OD_600_ = 0.1 was measured in batch cultures at 37°C with shaking at 250 rpm. Absorbance was read in one or two h intervals for 10 h using a BioTek SynergyH1 microplate reader (Biotek, Winooski, VT, United States). Two independent experiments containing three biological replicates were performed for each growth condition of interest. Growth rates were calculated for each replicate by taking the slope of the curve in the exponential growth phase (Hall et al., 2013).

### Motility Experiments

Swarming was examined on BM2 swarm plates supplemented with various sources of carbon and nitrogen, as previously mentioned, and 0.5% [wt/vol] agar. Surfing was examined on modified sputum-containing cystic fibrosis medium (MSCFM) supplemented with 0.4% [wt/vol] agar and 0.4% [wt/vol] mucin as previously described (Sun et al., 2018). Swimming and twitching of *P. aeruginosa* PA14 WT and mutants were examined on BM2 or LB plates supplemented with 0.3% [wt/vol] or 1.0% [wt/vol] agar, respectively. Briefly, subcultures were adjusted to a starting OD_600_ = 0.1 in appropriate medium and grown to an OD_600_ = 0.4–0.6 for spot (swarming, swimming, surfing) or stab (twitching) inoculation. Plates were incubated for 18–24 h at 37°C and, in twitching assays, another 24 h at room temperature. Plates were imaged with a BioRad ChemiDoc (BioRad, Montreal, QC) and surface area coverage of the plate was measured in ImageJ software (v1.52, NIH).^1^ Three independent experiments containing three biological replicates each were performed.

### Rhamnolipid Precursor Production

To explore the cellular mechanism underlying motility phenotypes of mutants, rhamnolipid precursor production was analyzed by the agar plate method as previously described (Zhang and Rainey, 2008). *P. aeruginosa* PA14 WT and mutants were grown overnight and spot inoculated onto iron-limited salt medium (0.7 g/l KH_2_PO_4_, 0.9 g/l NaHPO_4_, 2.0 g/l NaNO_3_, 0.4 g/l MgSO_4_ H_2_O, 0.001 g/l CaCl_2_ H_2_O, 0.001 g/l FeSO_4_ 7H_2_O) supplemented with 20 mM glucose, 0.1% [wt/vol] CAA, 0.02% [wt/vol] cetyltrimethylammonium bromide (CTAB), 0.0005% methylene blue and 1.5% [wt/vol] agar. Plates were incubated for 24 h at 37°C and another 96 h at room temperature. Rhamnolipid precursor production was measured by diameter of the zone of clearance around the colony. Two independent experiments containing three biological replicates each were performed.

### RNA Isolation and RNA-Seq

To characterize the molecular mechanism underlying adaptive phenotypes observed, we studied the transcriptomes of PA14 WT and *ntrBC* mutants under swarming conditions. PA14 strains were sub-cultured to an OD_600_ = 0.4–0.6 and spot cultured on BM2 swarming plates for 18–24 h at 37°C. Actively swarming cells were harvested from the tips of tendrils in PBS and RNAProtect reagent (Qiagen). RNA extraction was performed using the RNeasy Mini Kit (Qiagen) according to the manufacturer’s specifications. Deoxyribonucleases were removed using the TURBO DNA-free kit (Thermo Fisher Scientific) and rRNA was depleted using the RiboZero Bacteria Kit (Illumina). Single-end cDNA libraries were constructed using a KAPA stranded Total RNA Kit (KAPA Biosystems) and libraries were sequenced on an Illumina HiSeq 2500 platform in rapid run mode with 100 bp reads, excluding adapter/index sequences. RNA-Seq processing included quality control using fastqc (v0.11.7) and multiqc (v1.6), alignment to the *P. aeruginosa* PA14 genome using STAR (v2.6.0a), and read counting using htseq-count (v0.10.0) (Dobin et al., 2014; Anders et al., 2015; Ewels et al., 2016; Wingett and Andrews, 2018). Genome assembly and gene annotations were taken from the *Pseudomonas* Genome Database (Winsor et al., 2016). Genes with fewer than 10 counts in at least three samples were removed to increase detection power for differential expression (DE) analysis. The DESeq2 R package (v1.22.2) was used to perform DE analysis of PA14 mutants versus WT (Love et al., 2014). We considered a gene DE if the absolute fold change value was greater than 1.5 and adjusted *P* < 0.05. Gene Ontology (GO) enrichment was assessed using the GOFuncR package using GO annotations for the *P. aeruginosa* reference strain PAO1 (Grote, 2019).

### RT-qPCR

Real-time quantitative PCR (RT-qPCR) was used to validate expression of select dysregulated genes identified in mutants by RNA-Seq. Reaction samples were prepared using qScript one-step SYBR green RT-qPCR Kit (QuantaBio) with 0.2 ng/μl RNA. Amplification was performed using a LightCycler 96 instrument (Roche, Indianapolis, IN). Gene expression was quantified by the ΔΔC_t_ method with normalization to *rpoD* expression (Schmittgen and Livak, 2001). Primers used for qRT-PCR are listed in Supplementary Table S1.

### Statistical Analysis

Statistics were performed using GraphPad Prism 8.0 (La Jolla, CA, United States). *P-*values were calculated using Kruskal Wallis nonparametric test followed by Dunn’s *post hoc* analysis, two-tailed Welch’s *t*-test or two-tailed Fisher’s Exact test as indicated. Statistical significance established when *P* < 0.05.

## Results

### NtrBC Was Required for Full Virulence of *P. aeruginosa* LESB58 *in vivo*

**FIGURE 1:**
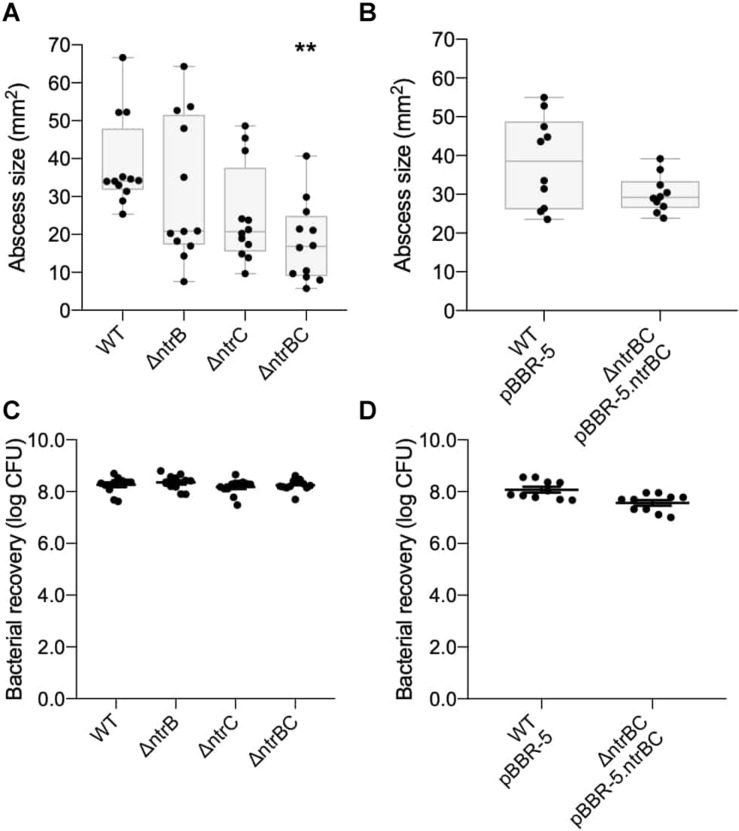
Virulence was reduced in an LESB58 mutant strain Δ*ntrBC* compared to the wild-type (WT) in a chronic model of CD-1 murine infection. Abscess size was significantly reduced in LESB58 Δ*ntrBC* compared to the WT **(A)**, but no different than WT control when transformed with plasmid carrying the *ntrBC* gene **(B)**. In contrast, bacterial recovery from abscesses formed by LESB58 mutants or WT were similar **(C,D)**. Briefly, mice were subcutaneously injected 5 ± 3 × 10^7^ planktonic cells and abscesses were formed for 72 h. At experimental endpoint, abscesses were measured and harvested in phosphate buffered saline (PBS), homogenized and plated on LB for bacterial enumeration. Box and whiskers delineate interquartile range with geometric error from four independent experiments containing 3–4 biological replicates each (*n* = 10–12) **(A,B)**. Otherwise, data reported as mean ± standard error of the mean (SEM) **(C,D)**. **P < 0.01 compared to WT according to Kruskal–Wallis nonparametric test followed by Dunn’s *post hoc* analysis.

The murine cutaneous abscess model of chronic infection (Pletzer et al., 2017) was used to examine if NtrBC had a role in the pathology associated with infections. *P. aeruginosa* LESB58 is a well-characterized cystic fibrosis isolate that causes chronic lung infection and disseminates less than PA14 from localized infection (abscess) sites, apparently due to less efficient flagella-mediated motility (Sousa and Pereira, 2014). This strain was used to test *in vivo* growth and virulence of the Δ*ntrB*, Δ*ntrC*, and Δ*ntrBC* mutants in long-term (72 h) infections. Compared to LESB58 WT, abscess size as measured by visible dermonecrosis resulting from Δ*ntrBC* infection was significantly (∼50%) reduced, but that of Δ*ntrB* and Δ*ntrC* was not significantly affected (Figure 1A). Complementation of Δ*ntrBC* by introduction of the deleted gene fragment on the cloning vector pBBR1MCS-5 restored the abscess size to that of WT (Figure 1B). In all cases, the number of bacteria recovered from abscesses was not different between LESB58 strains (Figures 1C,D).

### NtrBC Was Required for *P. aeruginosa* PA14 Dissemination *in vivo*

Prior transcriptomic characterization of *P. aeruginosa* PA14 swarming cells revealed upregulated expression of effectors that contribute to colonization and dissemination *in vivo* (Overhage et al., 2008). Forming high-density subcutaneous abscesses with mutants in the PA14 rather than LESB58 genetic background allowed us to examine this feature since this strain is capable of dissemination to distal organs, which is associated with significant mortality of mice within 36 h (Pletzer et al., 2017). Due to the deficiency in swarming motility in a strain PA14 *ntrC* mutant (Yeung et al., 2009), it was interesting to examine if this impacted on dissemination from a subcutaneous localized abscess to distal organs, including the heart, lungs, liver, spleen and kidneys of mice (Table 2 and Supplementary Figure S1).

**TABLE 2:** Invasiveness of PA14 *ntrBC* mutant strains was reduced in comparison to the wild-type (WT) in a CD-1 murine model of infection.

**Organ**	**Number of mice exhibiting bacteria in various**			
	**organs (bacterial counts; CFU)**			
	
	**WT**	**Δ*ntrB***	**Δ*ntrC***	**Δ*ntrBC***
Heart	8 (10^2^–10^6^)	2 (10^4^–10^5^)*	3 (10^2^–10^3^)*	0*
Lungs	9 (10^2^–10^6^)	7 (10^2^–10^5^)	3 (10^2^–10^4^)*	7 (10^2^–10^6^)
Liver	8 (10^2^–10^5^)	5 (10^2^–10^7^)	3 (10^3^–10^6^)*	1 (10^2^)*
Spleen	9 (10^2^–10^6^)	4 (10^2^–10^5^)*	4 (10^2^–10^5^)*	5 (10^3^–10^4^)*
Kidneys	7 (10^2^–10^7^)	2 (10^4^–10^5^)*	3 (10^3^–10^5^)	1 (10^3^)*

Briefly, mice were subcutaneously injected 10^5^–10^7^ planktonic cells and abscesses were formed for 24 h. At the experimental endpoint, organs were harvested in phosphate buffered saline (PBS), homogenized and plated on LB for bacterial enumeration. Dissemination of PA14 wild-type (WT) and ntrBC mutant strains from abscess to organs is shown as the frequency of bacterial recovery, and range of bacterial counts in instances of recovery, from four independent experiments each including 1–3 individual mice per bacterial strain (n = 9). Mutants were significantly reduced for dissemination to some organs compared to WT according to Fisher’s Exact Test (*P < 0.05).

Bacteria less frequently infiltrated the heart and spleen from abscesses formed by Δ*ntrB*, Δ*ntrC*, and Δ*ntrBC* mutants than from abscesses formed by the WT. In instances where mutant bacteria infiltrated the heart, fewer mutant bacteria were recovered (43- to 275-fold differences), similarly, 13 times less Δ*ntrC* bacteria were recovered from the spleen (Supplementary Figures S1A,D). Bacterial infiltration and numbers of bacteria in the lungs were significantly reduced (150-fold) in infections by the Δ*ntrC* mutant (Supplementary Figure S1B). Both Δ*ntrC* and Δ*ntrBC* demonstrated reduced infiltration of the liver, but the number of bacteria recovered was only reduced for Δ*ntrBC* abscesses (1440-fold) (Supplementary Figure S1C). Similarly, both Δ*ntrB* and Δ*ntrBC* demonstrated reduced infiltration of the kidneys, but the number of bacteria recovered was only reduced for the Δ*ntrBC* abscesses (17,500-fold) (Supplementary Figure S1E). Overall, invasiveness was most reduced in the PA14 Δ*ntrBC* double mutant relative to the WT.

### NtrBC Was Required for Complete Formation of Biofilms by *P. aeruginosa* PA14

**FIGURE 2:**
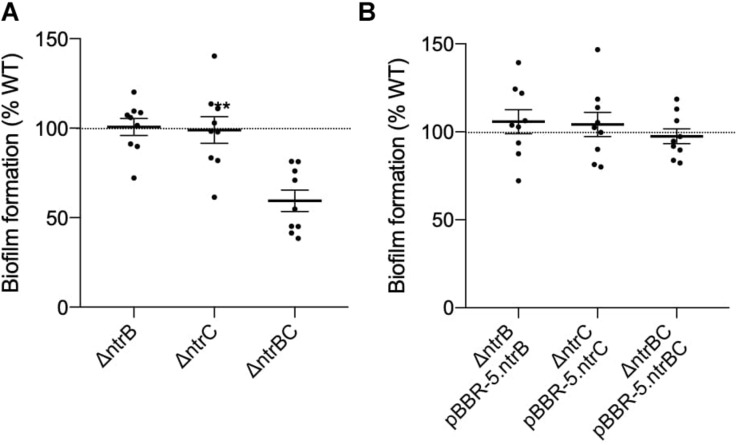
Biofilm formation was reduced in PA14 mutant strain Δ*ntrBC* compared to the wild-type (WT). **(A)** Biofilm formation was significantly reduced in PA14 Δ*ntrBC* compared to the WT. **(B)** Biofilm formation was similar in PA14 Δ*ntrBC* transformed with plasmid containing *ntrBC* and WT transformed with plasmid. Briefly, bacteria were seeded from overnight cultures into 96-well microtiter plates at low density (OD_600_ = 0.1) and incubated at 37°C for 24 h statically. Biomass formed in wells was washed and stained with 0.1% crystal violet (CV) prior to dissolution of aggregates with 70% ethanol. Biomass was measured (OD_595_) using a BioTek SynergyH1 microplate reader and taken relative to the WT. Data reported as mean ± standard error of the mean (SEM) from three independent experiments containing three biological replicates each (*n* = 9). **P < 0.01 according to Welch’s *t*-test.

*P. aeruginosa* biofilms represent a complex, adaptive sessile growth mode initiated by cell surface attachment to a substrate and switching of cellular physiological status (Boor, 2006; Fuente-Nunez et al., 2014; Haney et al., 2018). Klein et al. (2016) revealed that NtrBC had a role in regulating the production of biofilms by *E. coli* under nitrogen limiting conditions. Although the role of NtrBC in biofilm formation has not been tested in more closely related bacteria, NtrC activation in nitrogen limiting conditions in proteobacteria has been shown (Hervas et al., 2009). Biofilm formation of PA14 Δ*ntrB*, Δ*ntrC*, and Δ*ntrBC* mutants was assessed relative to the WT (Figure 2). Biofilm formation of the Δ*ntrBC* double mutant was significantly reduced (to ∼60% that of WT) whereas biofilm formation of Δ*ntrB* and Δ*ntrC* was similar to WT (Figure 2A). Biofilm formation was restored by complementation of Δ*ntrBC* with individual *ntrB* or *ntrC* (Figure 2B).

### NtrBC Influenced Growth of *P. aeruginosa* PA14 in Certain Nitrogen Sources

**FIGURE 3:**
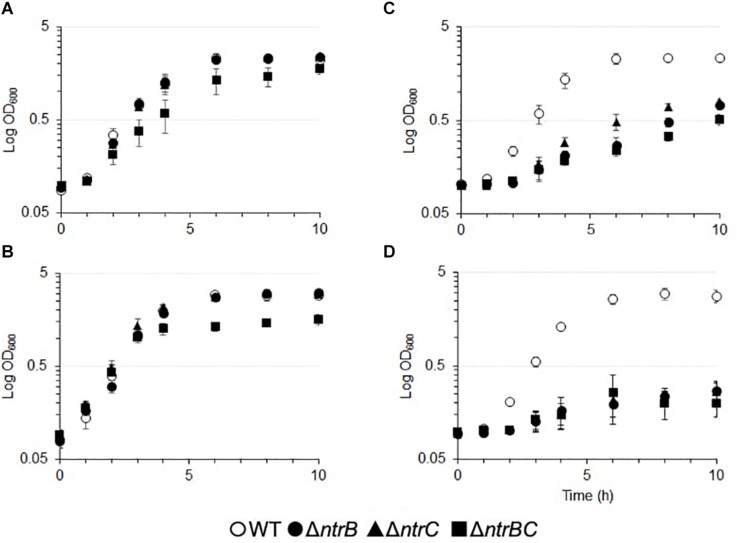
Growth of PA14 *ntrBC* mutant strains was influenced by nitrogen source and significantly reduced in the presence of nitrate or nitrite as well as casamino acids for the double mutant. Briefly, bacteria were seeded from overnight cultures into batch cultures at low density (OD_600_ = 0.1) and incubated at 37°C for 10 h with shaking in **(A)** basal medium (BM2) in which (NH_4_)_2_SO_4_ was replaced with **(B)** 0.1% casamino acids (CAA) **(C)** 14 mM NaNO_2_ or **(D)** 14 mM NaNO_3_. OD_600_ values were measured using an Eppendorf BioSpectrometer corrected for background absorbance. The mean logarithmic OD_600_ ± standard error of the mean (SEM) from three independent experiments is shown (*n* = 3). Complemented mutants were also tested and grew like the WT.

NtrBC has been described as a general nitrogen two component regulatory system that is responsive to intracellular glutamine levels in various bacterial species (Luque-Almagro et al., 2011; Bhagirath et al., 2019). We sought to determine the influence of NtrBC on growth using other nitrogenous compounds, such as NaNO_2_ and NaNO_3_, in the PA14 wild-type (WT) and Δ*ntrB*, Δ*ntrC*, and Δ*ntrBC* mutants (Figure 3 and Supplementary Table S2). There were no overall growth differences between single deletions grown in BM2 minimal medium that utilizes ammonium as a nitrogen source, although the kinetics of growth of the double deletion mutant was altered, with a slower growth rate (0.38/h versus 0.09/h) (Figure 3A). The double mutant also exhibited different growth kinetics and reduced overall growth in BM2 supplemented with 0.1% casamino acids (CAA) rather than (NH_4_)_2_SO_4_ (Figure 3B). No growth differences were observed between the WT and Δ*ntrB* or Δ*ntrC* under either of these conditions (approximately 0.37/h). In contrast, compared to the WT, each of Δ*ntrB*, Δ*ntrC*, and Δ*ntrBC* were reduced for overall growth in BM2 supplemented with equimolar NaNO_2_ or NaNO_3_ instead of (NH_4_)_2_SO_4_ (Figures 3C,D). The growth of mutants was reduced 3–10-fold in NaNO_2_ and 10–50-fold in NaNO_3_ compared to the WT. Complemented mutants were also tested and grew like the WT (not shown).

### NtrBC Was Required for Swarming Motility and Affected Surfing Motility

**FIGURE 4:**
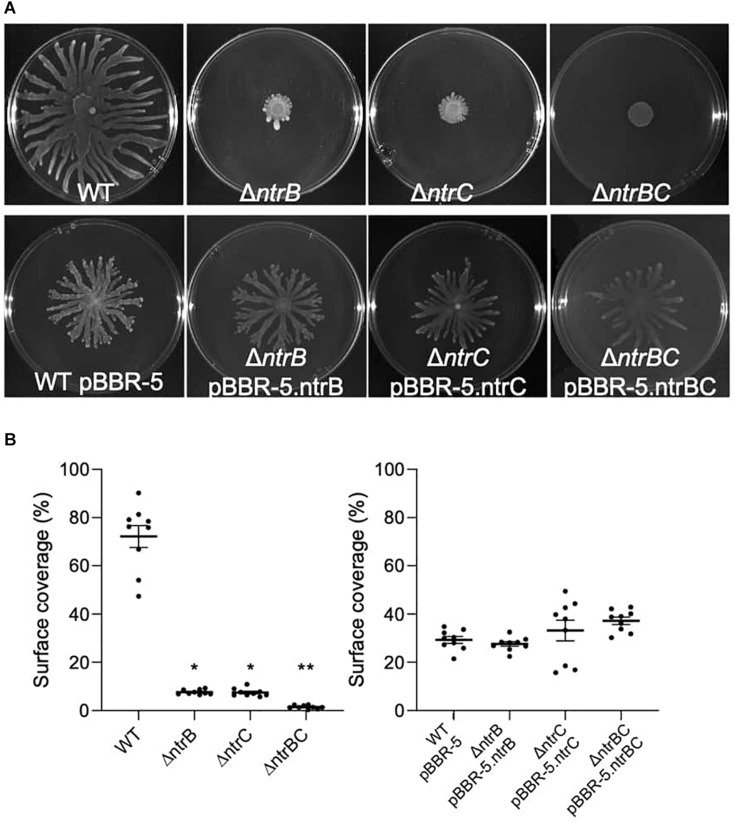
Swarming motility was dependent on both *ntrB* and *ntrC*. Shown are representative images of mutants and complemented strains. **(A)** Swarming motility was reduced or completely inhibited in PA14 mutant strains Δ*ntrB*,Δ*ntrC*, and Δ*ntrBC* compared to WT. Swarming motility in PA14 mutant strains transformed with plasmid containing *ntrB*, *ntrC*, or *ntrBC* genes was similar to WT transformed with plasmid. Swarm plates were inoculated with 5 μl of planktonic cells suspended at an OD_600_ = 0.4–0.6 in basal medium (BM2) supplemented with 0.1% casamino acids (CAA) and 0.4% glucose, then incubated for 18–24 h at 37°C. Images captured using a BioRad ChemiDoc. **(B)** Raw surface area coverage (%) of swarming colonies was assessed using ImageJ software. Data reported as mean ± standard error of the mean (SEM) from three independent experiments containing three biological replicates each (*n* = 9). *P < 0.05, **P < 0.01 according to Kruskal–Wallis nonparametric test followed by Dunn’s *post hoc* analysis.

**FIGURE 5:**
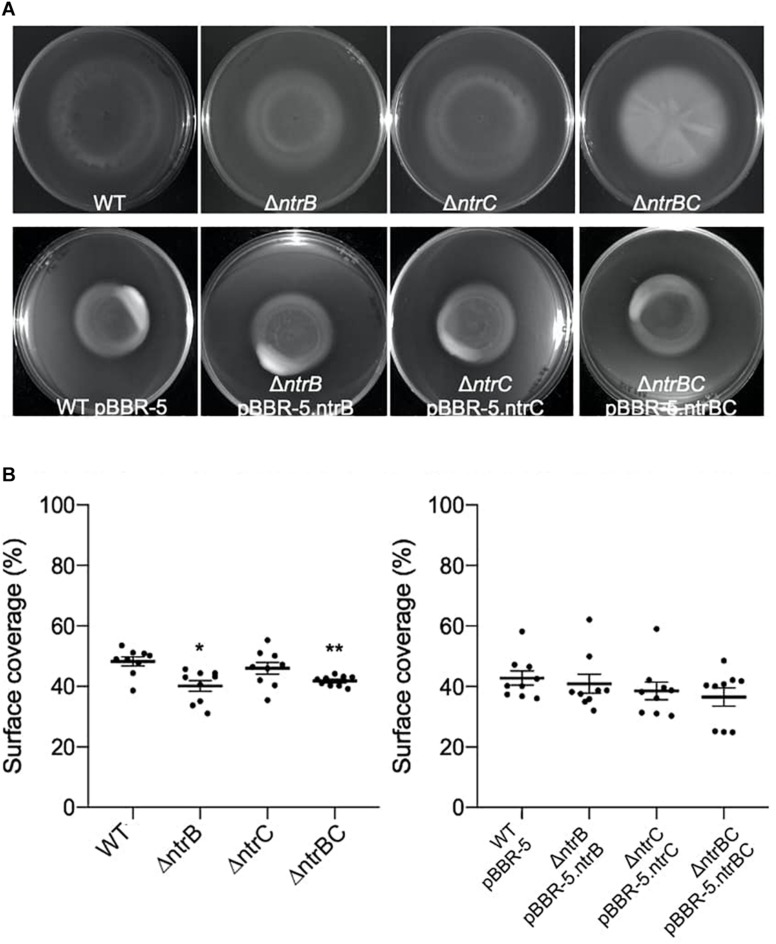
Surfing motility of PA14 was modestly reduced in mutants with *ntrB* deleted. **(A)** Surfing motility was reduced in PA14 mutant strains Δ*ntrB* and Δ*ntrBC* compared to WT. Surfing motility in PA14 mutant strains transformed with plasmid containing *ntrB*, *ntrC*, or *ntrBC* genes was similar to WT transformed with plasmid. Surf plates supplemented with 0.4% mucin were inoculated with 5 μl of planktonic cells suspended at an OD_600_ = 0.4–0.6 in MSCFM, then incubated for 18–24 h at 37°C. Images captured using a BioRad ChemiDoc. **(B)** Raw surface area coverage (%) of surfing colonies was assessed using ImageJ software. Data reported as mean ± standard error of the mean (SEM) from three independent experiments containing three biological replicates each (*n* = 9). *P < 0.05, **P < 0.01 according to Kruskal-Wallis nonparametric test followed by Dunn’s *post hoc* analysis.

Rapid surface motilities of *P. aeruginosa*, such as swarming and surfing, represent complex adaptive lifestyles that are regulated by multiple transcription factors and are dependent on the nutrients and viscosity of the media (Rodrigue et al., 2000; Brown et al., 2014; Sousa and Pereira, 2014). Since NtrBC contributes to *P. aeruginosa* responsiveness to nitrogen and has been implicated in swarming through transposon mutant screens (Yeung et al., 2009; Francis et al., 2018), we investigated the ability of PA14 WT, Δ*ntrB*, Δ*ntrC*, and Δ*ntrBC* to swarm under nitrogen-limiting conditions. Swarming of Δ*ntrB* and Δ*ntrC* was significantly reduced (∼8% surface coverage), whereas swarming of the double deletion Δ*ntrBC* was completely inhibited (∼1% surface coverage) relative to the WT (Figures 4A,B). Surfing motility, which is quite different from swarming and occurs in the presence of mucin that is added to mimic the cystic fibrosis lung environment (Yeung et al., 2012; Pletzer et al., 2020), was also investigated. It was found that surfing of PA14 Δ*ntrB* and Δ*ntrBC* mutants was significantly reduced compared to the WT, though the effect was considerably less (13.5–17.0% reductions) than that observed for swarming (Figures 5A,B). Additionally, the appearance of the Δ*ntrBC* mutant surfing colony was considerably different being thick throughout rather than just at the edge. Swimming and twitching motilities were unaffected by Δ*ntrB* or Δ*ntrC* mutations, with a modest but insignificant swimming effect observed only for the Δ*ntrBC* mutant (Table 3). Complementation of mutants by introduction of the respective deleted gene fragment restored swarming and surfing phenotypes to WT levels (Figures 4, 5).

**TABLE 3:** Swarming and surfing motilities of PA14 were reduced in *ntrBC* mutant strains compared to the wild-type (WT).

**Motility**	**WT**	**Δ*ntrB***	**Δ*ntrC***	**Δ*ntrBC***
Swarming	72.24.6%	7.660.33%*	7.490.54%**	1.450.24%**
Swimming	9.871.5%	7.850.94%	9.970.84%	9.241.4%
Twitching	6.510.42%	6.780.55%	6.640.47%	4.890.63%
Surfing	48.31.5%	40.11.8%*	46.02.0%	41.80.55%**

Briefly, swarm and swim plates were inoculated with 5 μl of planktonic cells at an OD_600_ = 0.4–0.6 in basal medium (BM2) supplemented with 0.1% casamino acids or 7 mM (NH_4_)_2_SO_4_ and 0.4% glucose, then incubated for 18–24 h at 37°C. Twitch plates were inoculated with bacteria suspended in Luria broth (LB) and surf plates were inoculated with bacteria suspended in modified sputum-containing cystic fibrosis medium (MSCFM), but otherwise treated similar. Motility of PA14 WT and ntrBC mutants shown as mean surface area coverage ± standard error of the mean from three independent experiments containing three biological replicates each (n = 9). Mutants were significantly reduced for swarming and, more modestly, surfing motilities compared to WT according to a Kruskal–Wallis nonparametric test followed by Dunn’s post hoc analysis (*P < 0.05, **P < 0.01).

Since it has been observed that NtrBC is important for growth on various nitrogen sources (Figure 3) and research has implied a role in carbon/nitrogen balance of *Pseudomonas* (Zhang and Rainey, 2008), we examined the influence of specific nitrogen and carbon growth substrates on the swarming phenotype of PA14 WT (Supplementary Figures S2, S3). Substitution of casamino acids (CAA) in BM2 swarming media with equimolar amounts of ammonium sulfate and urea, but not with glutamate or NaNO_2_, significantly reduced swarming motility of PA14 WT by 70.8–74.4% (Supplementary Figures S2A,B). Although substitution of CAA for NaNO_3_ caused a modest (23.3%) reduction in swarming, the effect was not statistically significant. Interestingly the swarming colony had quite different branching patterns on each of the permissive nitrogen sources suggesting that this feature might also be under nitrogen source control. Similarly, substitution of glucose in BM2 swarming media with equimolar amounts of malate and succinate, but not citrate, significantly reduced swarming motility of PA14 WT by 8.6–46.8% and also led to a change in the morphology of the swarming colony (Supplementary Figures S3A,B).

### NtrBC Influenced Production of Rhamnolipids by *P. aeruginosa* PA14

**FIGURE 6:**
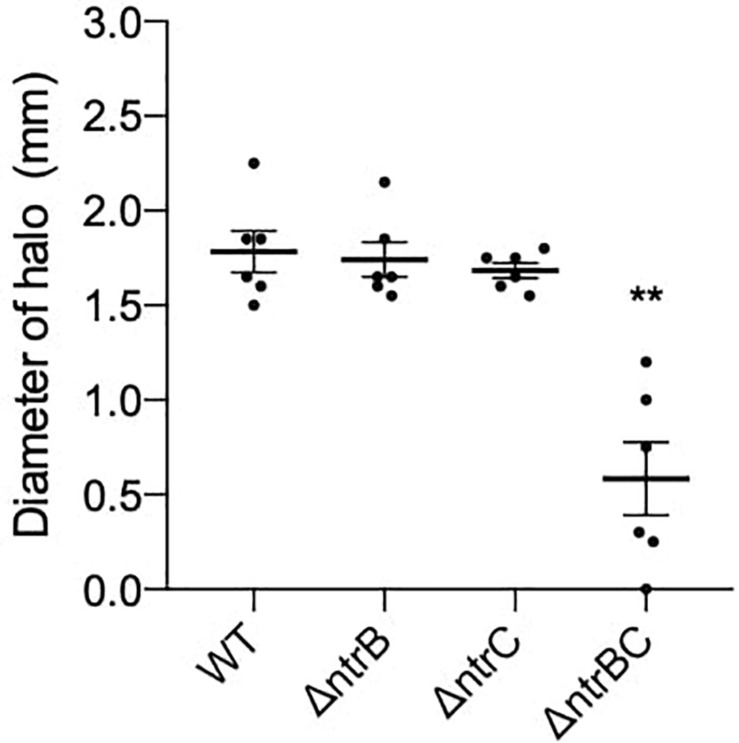
Rhamnolipid precursor production was significantly reduced in the Δ*ntrBC* double mutant when compared to the WT. Diameter of halo (mm) was measured following 120 h static incubation at room temperature (RT) on iron-limited salt medium. Data reported as mean ± standard error of the mean (SEM) from three independent experiments containing two biological replicates each (*n* = 6). **P < 0.05 according to Kruskal–Wallis nonparametric test followed by Dunn’s *post hoc* analysis.

Rhamnolipids produced by *P. aeruginosa* reduce the surface tension between bacterial cells and growth medium and are necessary for swarming but not surfing motility (Harshey, 2003; Caiazza et al., 2005). To determine whether rhamnolipid production was affected in Δ*ntrB*, Δ*ntrC* and Δ*ntrBC* mutants, we examined their ability to produce rhamnolipid precursors by a well-established agar plate method (Deziel et al., 1996). Rhamnolipid precursor production was reduced on average 67% in the Δ*ntrBC* double mutant as indicated by the smaller zone of clearance surrounding colonies, but precursor production in the Δ*ntrB* and Δ*ntrC* mutants was similar to WT levels (Figure 6A).

### NtrBC as a Global Regulator That Influenced Expression of Metabolism and Virulence Genes

**FIGURE 7:**
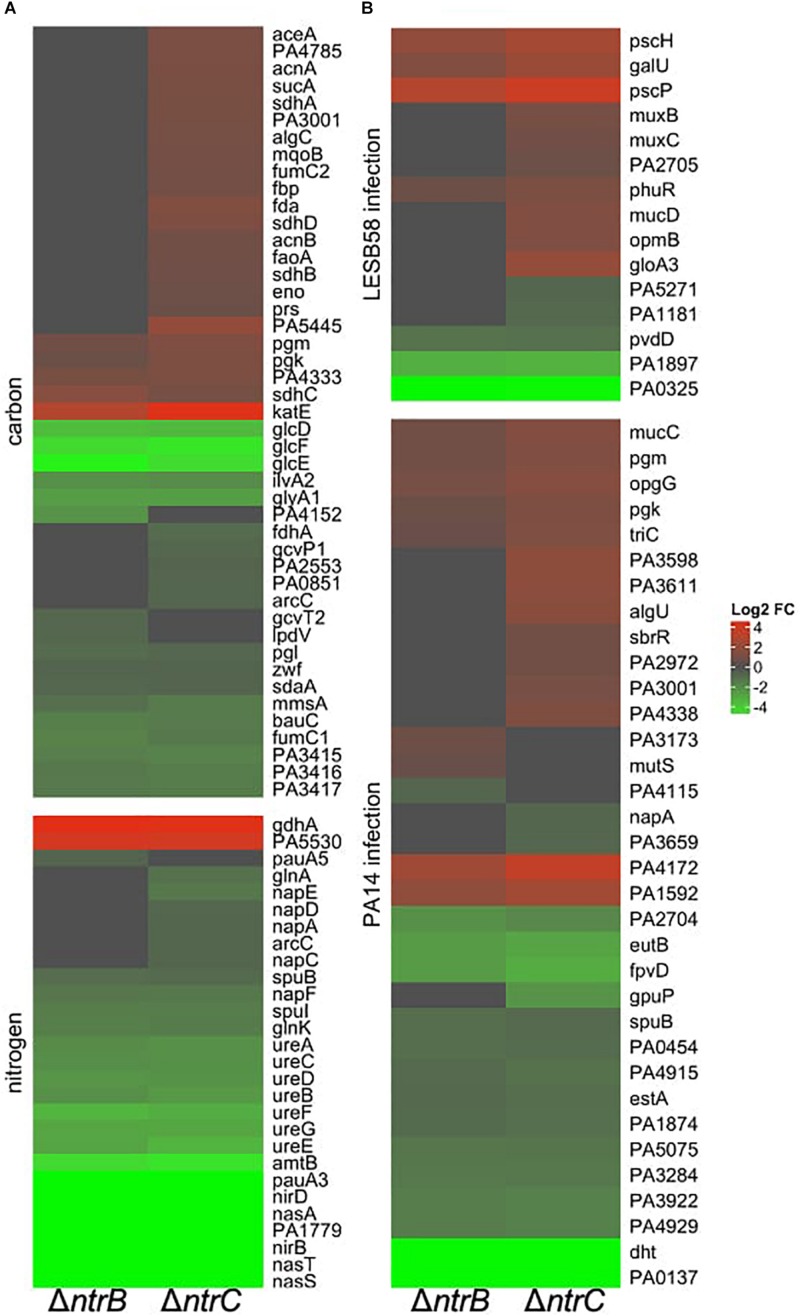
NtrBC was a global regulator that influenced expression of genes involved in physiological processes other than nitrogen metabolism. Heatmaps are shown for differentially expressed (DE) genes implicated in **(A)** carbon or nitrogen metabolism and **(B)** virulence in LESB58 or PA14 infection. Briefly, swarm plates were inoculated with 5 μl of planktonic cells suspended at an OD_600_ = 0.4–0.6 in basal medium (BM2) supplemented with 0.1% casamino acids (CAA) and 0.4% glucose, then incubated for 18–24 h at 37°C. Swarming cells were harvested from the tip of the swarm tendrils and RNA was isolated using Qiagen RNEasy MiniPrep kit.

To further characterize the molecular mechanisms by which NtrBC contributes to adaptive growth states, RNA-Seq was performed and compared the transcriptomes of PA14 Δ*ntrB* and Δ*ntrC* mutants to WT under swarming conditions. Differentially expressed (DE) genes were identified as those with absolute log_2_ fold-change (FC) greater than 1.5 and adjusted (for false discovery rates) *P* < 0.05. These mutations influenced the transcriptome of PA14, with 790 and 1184 genes dysregulated in Δ*ntrB* and Δ*ntrC*, respectively, of which 682 genes were commonly dysregulated. Since there were no growth differences between WT and mutants under these conditions, it was unlikely that expression profiles were influenced by fitness. The large number of commonly dysregulated genes strongly indicated that in many cases NtrB and NtrC acted as a cognate pair. Conversely, the differences observed, somewhat weighted toward the Δ*ntrC* mutant, were consistent with phenotypic differences between the two mutants, e.g., in surfing (Figure 7), and the observation that these individual components in the NtrBC two component regulatory system when deleted did not lead to the same phenotypes as the double mutant, indicating potential cross talk with other regulators.

Several dysregulated genes are involved in general nitrogen and carbon metabolic processes according to the Kyoto Encyclopedia of Genes and Genomes (KEGG)^2^ database (Figure 7A and Table 4). Regarding nitrogen metabolism, genes involved in ammonium uptake, nitrate and nitrite assimilation, glutamine or glutamate synthesis and metabolism, and urea detoxification and assimilation were downregulated by 1.5–650-fold, generally in both mutants (Table 4A). Significant downregulation was also observed for many important carbon metabolism genes (Table 4B) including cognate pyruvate dehydrogenase genes PA3415-PA3417 and other pyruvate dehydrogenase genes such as PA4152 and *aceEF*, which encode proteins that convert pyruvate into acetyl-CoA for induction of the TCA cycle (Janssen et al., 1980). Nearly all enzymes involved in the TCA cycle were dysregulated; for example, fumarase (*fumC1*) and succinate dehydrogenase (*sdhABCD*), which catalyze the reduction of nicotinamide adenine nucleotide (NAD^+^) for shuttling electrons to the electron transport chain (ETC) were upregulated, although other functionally redundant genes were downregulated (*fumC2*).

**TABLE 4:** Selected categories of genes were differentially expressed under swarming conditions in PA14 *ntrB* and/or *ntrC* mutant strains.

**PAO1 locus**	**Name**	**Annotation**	**FC Δ*ntrB***	**FC Δ*ntrC***
**A. Metabolic genes involved in nitrogen metabolism (KEGG)**				
PA0296	*spuI*	Glutamylpolyamine synthetase	−2.57	−2.56
PA0298	*spuB*	Glutamylpolyamine synthetase	−1.87	−1.73
PA3356	*pauA5*	Glutamine synthetase	−1.51	−1.47
PA1783	*nasA*	Nitrate transporter	−16	−650
PA1781	*nirB*	Assimilatory nitrite reductase large	−50	−137
PA1780	*nirD*	Assimilatory nitrite reductase small	−64.2	−340
PA1779	–	ASSIMILATORY nitrate reductase	−39.9	−90.0
PA1566	*pauA3*	Glutamylpolyamine synthetase	−42.6	−42.0
PA1172	*napC*	Cytochrome c-type protein NapC	−1.08	−1.59
PA1174	*napA*	Nitrate reductase catalytic subunit	−1.29	−1.60
PA1175	*napD*	NapD protein of periplasmic nitrate reductase	−1.13	−1.64
PA1176	*napF*	Ferredoxin component of nitrate reductase	−2.29	−2.22
PA1177	*napE*	Periplasmic nitrate reductase NapE	−1.62	−2.27
PA1785	*nasT*	Regulation of nitrate assimilation	−92.8	−83.4
PA1786	*nasS*	Nitrate binding ABC transport protein	−121	−91.2
PA4588	*gdhA*	Glutamate dehydrogenase	17.2	16.5
PA4864	*ureD*	Urease accessory protein	−3.91	−4.13
PA4865	*ureA*	Urease gamma subunit	−3.37	−3.72
PA4867	*ureB*	Urease beta subunit	−3.80	−4.46
PA4868	*ureC*	Urease alpha subunit	−3.60	−3.80
PA4891	*ureE*	Urease accessory protein UreE	−7.86	−5.75
PA4892	*ureF*	Urease accessory protein UreF	−6.70	−7.79
PA4893	*ureG*	Urease accessory protein UreG	−5.89	−5.97
PA5119	*glnA*	Glutamine synthetase	−1.92	−1.98
PA5173	*arcC*	Carbamate kinase	−1.25	−1.58
PA5287	*amtB*	Ammonium transporter AmtB	−19.2	−15.7
PA5288	*glnK*	Nitrogen regulatory protein P-II	−2.51	−2.68
PA5530	–	C5−dicarboxylate transporter	13.3	12.4
**B. Metabolic genes involved in central carbon metabolism (KEGG)**				
PA0130	*bauC*	Aldehyde dehydrogenase	−2.67	−2.45
PA0552	*pgk*	Phosphoglycerate kinase	1.57	2.19
PA0555	*fda*	Fructose-1,6-bisphosphate aldolase	2.02	2.43
PA0851	–	Hypothetical protein	−1.54	−1.56
PA0854	*fumC2*	Fumarate hydratase	1.52	1.85
PA1326	*ilvA2*	Threonine dehydratase	−3.84	−3.45
PA1562	*acnA*	Aconitate hydratase	1.39	2.07
PA1581	*sdhC*	Succinate dehydrogenase, cytochrome b556 subunit	2.71	1.91
PA1582	*sdhD*	Succinate dehydrogenase (D subunit)	2.20	2.23
PA1583	*sdhA*	Succinate dehydrogenase flavoprotein subunit	1.70	2.00
PA1584	*sdhB*	Succinate dehydrogenase iron−sulfur subunit	1.55	1.68
PA1585	*sucA*	2-oxoglutarate dehydrogenase E1	1.93	2.06
PA1787	*acnB*	Bifunctional aconitate hydratase	1.60	1.77
PA2147	*katE*	Hydroperoxidase II	6.56	16.2
PA2250	*lpdV*	Lipoamide dehydrogenase-Val	−1.62	−1.31
PA2442	*gcvT2*	Glycine cleavage system protein T2	−1.62	−1.47
PA2443	*sdaA*	L−serine dehydratase	−1.64	−1.52
PA2553	–	Acyl−CoA thiolase	−1.34	−1.53
PA2634	*aceA*	Isocitrate lyase	2.00	2.10
PA3001	*gapA*	Glyceraldehyde-3-phosphate dehydrogenase	1.75	1.96
PA3014	*faoA*	Multifunctional fatty acid oxidation complex subunit α	1.74	1.75
PA3182	*pgl*	6-phosphogluconolactonase	−1.80	−1.63
PA3183	*zwf*	Glucose-6-phosphate 1-dehydrogenase	−1.55	−1.52
PA3415	–	Probable dihydrolipoamide acetyltransferase	−2.61	−2.80
PA3416	*pdhB*	Prob. pyruvate dehydrogenase E1 component, β chain	−2.28	−2.55
PA3417	–	Pyruvate dehydrogenase E1 component subunit alpha	−2.39	−2.53
PA3570	*mmsA*	Methylmalonate-semialdehyde dehydrogenase	−1.92	−2.44
PA3635	*eno*	Phosphopyruvate hydratase	1.61	1.61
PA4152	–	Branched-chain α-keto acid dehydrogenase subunit E2	−3.96	−1.81
PA4333	*fumA*	Fumarase	2.00	2.13
PA4470	*fumC1*	Fumarate hydratase	−2.84	−2.31
PA4640	*mqoB*	Malate:quinone oxidoreductase	1.57	1.87
PA4670	*prs*	Ribose-phosphate pyrophosphokinase	1.62	1.57
PA4785	*yfcY*	Acetyl-CoA acetyltransferase	1.26	2.10
PA5110	*fbp*	Fructose-1,6-bisphosphatase	1.36	1.83
PA5131	*pgm*	Phosphoglyceromutase	1.75	2.31
PA5173	*arcC*	Carbamate kinase	1.75	−1.58
PA5192	*pckA*	Phosphoenolpyruvate carboxykinase	1.26	1.35
PA5213	*gcvP1*	Glycine dehydrogenase	−1.48	−1.61
PA5322	*algC*	Phosphomannomutase	1.58	1.90
PA5353	*glcF*	Glycolate oxidase subunit GlcF	−15.6	−20.0
PA5354	*glcE*	Glycolate oxidase subunit GlcE	−25.5	−16.5
PA5355	*glcD*	Glycolate oxidase subunit GlcD	−9.09	−8.42
PA5415	*glyA1*	serine hydroxymethyltransferase	−4.76	−4.96
PA5421	*fdhA*	Glutathione-independent formaldehyde dehydrogenase	−1.45	−1.79
PA5445	–	Coenzyme A transferase	1.87	3.38
**C. Pathogenicity genes required for PA14 virulence in rat chronic lung infection**				
PA0098	–	3−oxoacyl−ACP synthase	−112	−153
PA0158	*triC*	RND efflux transporter	1.51	2.22
PA0287	*gpuP*	Sodium:solute symporter	−2.41	−4.01
PA0298	*spuB*	Glutamine synthetase	−1.87	−1.73
PA0441	*dht*	Phenylhydantoinase	−38.2	−48.4
PA0454	–	Hypothetical protein	−1.83	−1.8
PA0552	*pgk*	Phosphoglycerate kinase	1.57	2.19
PA0762	*algU*	RNA polymerase sigma factor AlgU	1.45	2.78
PA0765	*mucC*	Positive regulator for alginate biosynthesis MucC	1.76	2.47
PA1174	*napA*	Nitrate reductase catalytic subunit napA	−1.28	−1.60
PA1596	*htpG*	Heat shock protein 90	3.14	4.47
PA1874	–	Hypothetical protein	−1.75	−1.89
PA2408	*fpvD*	ABC transporter ATP-binding protein	−4.64	−6.59
PA2704	–	AraC family transcriptional regulator	−3.85	−3.15
PA2895	*sbrR*	SbrR	−1.08	1.76
PA2972	–	Maf−like protein	1.53	1.75
PA3001	–	Glyceraldehyde-3-phosphate dehydrogenase	1.75	1.96
PA3173	–	Short chain dehydrogenase	1.65	1.34
PA3284	–	Hypothetical protein	−2.26	−2.27
PA3598	–	Hypothetical protein	1.44	2.98
PA3611	–	Hypothetical protein	2.17	2.93
PA3620	*mutS*	DNA mismatch repair protein MutS	1.50	1.39
PA3659	–	Succinyldiaminopimelate transaminase	−1.41	−1.61
PA3922	–	Hypothetical protein	−2.53	−2.75
PA4024	*eutB*	Ethanolamine ammonia-lyase large subunit	−4.66	−5.59
PA4172	–	Hypothetical protein	−1.58	8.93
PA4308	–	Exonuclease III	−1.03	1.04
PA4338	–	Hypothetical protein	1.49	2.26
PA4659	–	MerR family transcriptional regulator	2.72	1.96
PA4915	–	Methyl−accepting chemotaxis protein	−1.72	−2.04
PA4929	–	Hypothetical protein	−2.56	−2.77
PA5075	–	ABC transporter permease	−2.21	−2.18
PA5078	*opgG*	Glucan biosynthesis protein G	1.94	2.57
PA5112	*estA*	Esterase EstA	−1.7	−1.88
PA5131	*pgm*	Phosphoglyceromutase	1.75	2.31
**D. Pathogenicity genes required for lesb58 virulence in rat chronic lung infection**				
PA1695	*pscP*	Translocation protein in type III secretion	6.67	10.7
PA2399	*pvdD*	Pyoverdine synthetase D	−2.09	−2.00
PA2525	*opmB*	Outer membrane protein	1.49	2.32
PA2526	*muxC*	Efflux transporter	1.26	1.78
PA2527	*muxB*	RND efflux transporter	1.41	1.95
PA0325	–	ABC transporter permease	−36.4	−31.0
PA2705	–	Hypothetical protein	1.20	1.62
PA2023	*galU*	UTP-glucose-1-phosphate uridylyltransferase	2.36	3.86
PA1897	–	Hypothetical protein	−6.91	−7.42
PA1721	*pscH*	Type III export protein PscH	3.24	4.79
PA1181	–	Sensor protein	−1.62	−1.68
PA0766	*mucD*	Serine protease MucD	1.75	2.40
PA4710	*phuR*	Heme/hemoglobin uptake outer membrane receptor	1.60	2.16
PA5111	*hsiC3*	Lactoylglutathione lyase	2.15	−2.71
PA5271	–	Hypothetical protein	−1.04	−1.59

*Genes in four categories that were differentially expressed according to RNA−Seq in PA14 mutant strains. Gene expression for mutants is reported as fold-change (FC) relative to PA14 wild-type (WT). Briefly, swarm plates were inoculated with 5 μl of planktonic cells suspended at an OD_600_ = 0.4–0.6 in basal medium (BM2) supplemented with 0.1% casamino acids and 0.4% glucose, then incubated for 18–24 h at 37°C. Swarming cells were harvested from the tip of the swarm tendrils and RNA was isolated using Qiagen RNEasy MiniPrep kit.*

There was also differential expression of genes necessary for full virulence in rat models of PA14 or LESB58 lung infection (Figure 7B and Tables 4C,D) consistent with our *in vivo* studies that indicated a role for NtrBC in *P. aeruginosa* pathogenicity (Figure 1, Supplementary Figure S1, and Table 2; Potvin et al., 2003; Winstanley et al., 2009). Downregulated pathogenicity genes included a putative 3-oxoacyl-ACP synthase PA0098 (−112 to −153-fold downregulated), an ABC transport permease PA0325 (−36 to −31-fold), *dht* (−38 to −48-fold downregulated), ferri-pyoverdine transporter *fpvD* (−4.6 to −6.6-fold downregulated), pyoverdine synthase D (−2-fold downregulated) ethanol ammonia lyase *eutB* (−4.6 to −6.6-fold downregulated), hypothetical protein PA1897 (−6.9 to −7.4-fold downregulated) and 16 others. Genes that intersect virulence and metabolism were the most downregulated including PA0098 and *dht*, which are involved in catabolism of fatty and amino acids, respectively. Other downregulated genes that intersect metabolism and virulence included *gpuP*, *glnA*, *pgk*, and *hsiC3*. Other genes involved in virulence were more upregulated than downregulated in Δ*ntrB* and Δ*ntrC.* These genes included *muxABC* and *opmB*, components of a resistance-nodulation-cell division (RND)-type multidrug efflux pump, as well as *pscH, pscP*, and *phuR*, that encode extracellular membrane proteins involved in type-III secretion and heme uptake, respectively (Winsor et al., 2016). The DNA repair protein exonuclease III (PA4172) was the most highly upregulated (by 4.2–8.9-fold) of these virulence genes of interest.

## Discussion

In this study, the role of NtrBC in *in vivo* invasiveness and virulence was examined, as well as biofilm formation and bacterial growth and motility in the presence of different nitrogen- and carbon- containing compounds. Deletion mutants Δ*ntrB*, Δ*ntrC*, and Δ*ntrBC* exhibited significantly reduced growth in the presence of NaNO_3_ or NaNO_2_ and, for the double deletion only, moderately reduced growth in the presence of (NH_4_)_2_SO_4_ or CAA as the sole nitrogen source (Figure 3). Reduced growth in the presence of nitrate and nitrite was predicted since NtrC is known to activate the expression of several key genes involved in nitrate assimilation in other species of proteobacteria (Luque-Almagro et al., 2011; Bhagirath et al., 2019). Accordingly, the gene expression data presented here indicated that transcription of *nas* (nitrate assimilation), *nir* (assimilatory nitrite reductase) and PA1779 (assimilatory nitrate reductase) were downregulated by 92.8–650.6-fold in Δ*ntrB* and Δ*ntrC* mutants under swarming motility conditions that require weaker nitrogen sources (Figure 7 and Table 4).

These data reinforce the concept that many adaptive growth phenotypes of bacteria, including adaptation to the infection environment mice, are dependent on the nutritional status of the environment (Fuente-Nunez et al., 2013, 2014), indicating it is important to improve our understanding of the sophisticated mechanisms underlying *P. aeruginosa* carbon and nitrogen metabolism. Consistent with this, a targeted screen of 113 two-component system genes in *P. aeruginosa* PA14 revealed that 44 regulate swarming in a context-dependent fashion (Kollaran et al., 2019). Our experiments showed that swarming of PA14 WT was inhibited by the substitution of CAA in swarming media with equimolar urea or (NH_4_)_2_SO_4_ but not NaNO_3_, NaNO_2_ or glutamate, all of which supported swarming (Supplementary Figure S2). These results are interesting when considered in the context of infection, since *P. aeruginosa* preferentially uses nitrate for efficient growth in anoxic environments (Toyofuku and Yoon, 2018). Moreover, bacterial detoxification of nitrates and nitrites is essential for surviving host responses and contributes to redox homeostasis and fitness (Vasquez-Torres and Baumler, 2017).

Swarming of PA14 WT was also inhibited by substitution of glucose in swarming media for equimolar succinate or malate, but not citrate (Supplementary Figure S2). Expression of *sdh* (succinate dehydrogenase), *mdh* (malate dehydrogenase), and PA4333 (probable fumarase) genes was upregulated in mutants under nitrogen limiting conditions (Table 4). Succinate and malate are quickly converted to oxaloacetate or pyruvate through metabolic reactions in the oxidative portion of the TCA cycle (Zhang and Rainey, 2008; Rohmer et al., 2011). These metabolites positively regulate TCA cycle activity and prevent carbon flux through the glyoxylate shunt, a competing metabolic pathway with a role in mediating bacterial oxidative stress (Flynn et al., 2017; Crousilles et al., 2018). Reduced ability of *P. aeruginosa* to swarm in the presence of these nutrients, taken with previous observations that PA14 Δ*ntrBC* mutants are reduced for human bronchial epithelial cell directed cytotoxicity and adherence (Gellatly et al., 2018), clearly suggests an adaptive role for carbon and nitrogen metabolism in pathogenesis. This argument is further strengthened by motility experiments that revealed swarming and surfing defects of PA14 Δ*ntrBC* mutants (Figures 5, 6). Since swimming and twitching motilities were unaffected, it is unlikely that modifications of flagella or type IV pili (bacterial appendages required for swimming and twitching, respectively) were the cause of mutant swarming defects (Table 2). Motility defects of mutants might be partially explained by poorer production of rhamnolipids, which facilitate swarming motility by “lubrication” or reduction of surface tension between bacterial cells and the media (Figure 6; Overhage et al., 2008; Zhang and Rainey, 2008). Mutant phenotypes exhibited for surfing motility were less drastic than for swarming motility, in part because surfing does not depend on rhamnolipid production (although the lower apparent dependence of surfing on a poor nitrogen source might also play a role; Sun et al., 2018).

Since NtrC is annotated as an enhancer of RpoN, which influences expression of approximately a fifth of the *P. aeruginosa* genome (Thoma and Schobert, 2009) and induces cascading transcription of numerous regulatory genes, we anticipated a greater number of genes to be dysregulated in Δ*ntrC* than Δ*ntrB* compared to the WT. Indeed, deletion of *ntrC* caused dysregulated expression of 1,192 genes, whereas deletion of *ntrB* caused dysregulation of 791 genes, 686 of which were commonly dysregulated in Δ*ntrB* and Δ*ntrC.* This result suggests divergence in regulons of NtrB and NtrC, although further experiments are needed for validation. Nonetheless, the RNA-Seq results described support our hypothesis that NtrB and NtrC have direct overlapping but unique influences on adaptive lifestyles since genes dysregulated in their mutants diverged. The activity of NtrB and NtrC may be at least partly independent of RpoN since there are important differences in gene expression across mutants. For example, RpoN mutants directly and substantially down-regulated type VI secretion as well as quorum sensing, exhibiting dysregulated expression of the *pqs* (*Pseudomonas* quinolone signal) and *lasRI* genes, while upregulating rhamnolipids production (Liberati et al., 2006; Damron et al., 2012; Cai et al., 2015). This study showed that while a few type VI secretion genes such as *vgrG* and *clpV* were modestly downregulated ∼2-fold in *ntrC* mutants (Supplementary Table S3), *pqs* and other quorum sensing genes were not affected in Ntr mutants, while rhamnolipid production was suppressed in the double mutant.

## Conclusion

In conclusion, the data presented elucidates a major and multifactorial role for NtrBC in virulence. Moreover, we revealed an expanded role for NtrBC in metabolism of carbon- and nitrogen-containing compounds that was not previously characterized. Since we provide substantial data that this two-component system is important for optimal adaptation, we propose NtrBC should be considered as a global regulatory system that contributes to the physiological balance of *P. aeruginosa* particularly during infection and complex adaptive lifestyles. Although NtrBC deficiency does not decrease overall bacterial load in the abscess, we know that interefering with stress-response effector proteins provide a means of dismantling bacterial virulence for treating infectious disease in combination with conventional antibiotics (Alford et al., 2019). Further, we previously showed that a broad range of pathogens can be targeted and sensitized to conventional antibiotic therapy in our cutaneous model of high-density bacterial infection by attacking stringent stress response using novel synthetic peptides (Mansour et al., 2016; Pletzer et al., 2017; Pletzer and Hancock, 2018). Inhibitors of NtrBC might represent a novel class of compounds that can be used to treat recalcitrant and invasive nosocomial infections in combination with antibiotics.

## Data Availability Statement

The datasets generated for this study can be found in the Gene Expression Omnibus (GEO) GSE145591 (https://www.ncbi.nlm.nih.gov/geo/query/acc.cgi?acc=GSE145591).

## Ethics Statement

The animal study was reviewed and approved by University of British Columbia Animal Care Committee (A14-0253).

## Author Contributions

MA was responsible for investigation, validation and visualization of data, formal analysis, writing (drafting and editing), and project administration. AB was responsible for application of software, formal analysis and visualization of data, and writing (drafting and editing). AY was responsible for pilot experiments. DP provided technical guidance in many of the assays performed. RH was responsible for funding acquisition, provision of resources and supervision, project administration, and writing (editing). All authors approved the final version of the manuscript.

## Conflict of Interest

The authors declare that the research was conducted in the absence of any commercial or financial relationships that could be construed as a potential conflict of interest.

## Footnotes

1 https://imagej.nih.gov/ij/

2 ^2^https://www.genome.jp/kegg/
