# Supplementary material for: NtrBC Regulates Invasiveness and Virulence of Pseudomonas aeruginosa During High-Density Infection
Source: Front Microbiol. 2020 May 5;11:773. doi: 10.3389/fmicb.2020.00773 (PMC7214821; doi:10.3389/fmicb.2020.00773)
Supplement: Supplementary file 1 [file Data_Sheet_1.docx]

NtrBC regulates invasiveness and virulence of *Pseudomonas aeruginosa* during high-density infection

**
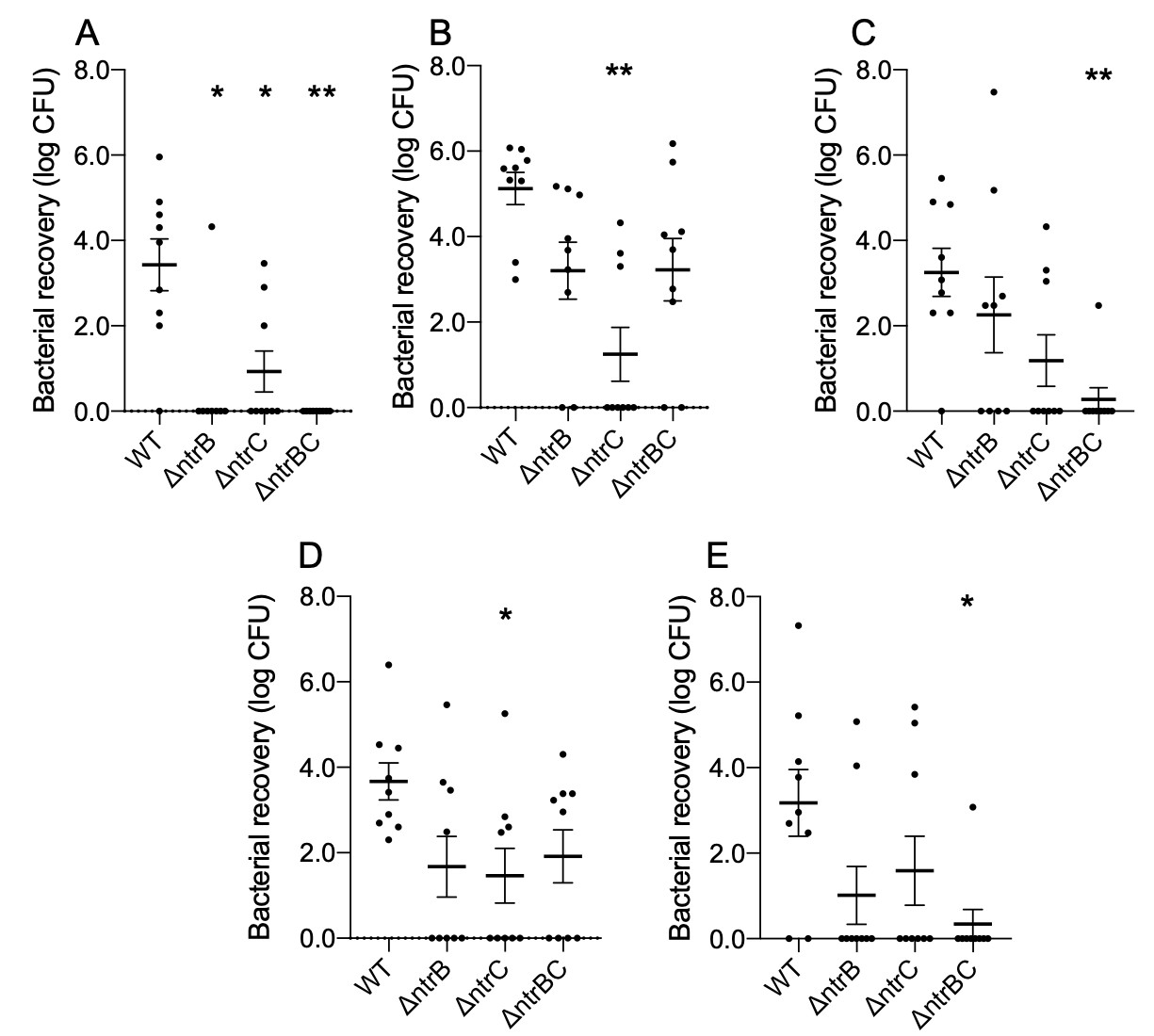
**

**Figure S1. Dissemination of mutants from abscesses to organs was reduced in mutants compared to PA14 wild-type (WT) in an acute model of CD-1 infection.** Briefly, mice were subcutaneously injected 5±3 x 10^7^  CFU planktonic cells and abscesses were formed for 24 h. At the experimental endpoint, organs were harvested in phosphate buffered saline (PBS), homogenized and plated on LB for bacterial enumeration. Organs included the heart (A), lungs (B), liver (C), spleen (D) and kidney (E). Data are presented as mean ± standard error of the mean (SEM) from four independent experiments each including 1-3 individual mice per bacterial strain *(n* = 9*).* * *P* < 0.05, ** *P* < 0.01 according to Kruskal-Wallis nonparametric test followed by Dunn’s post-hoc analysis.


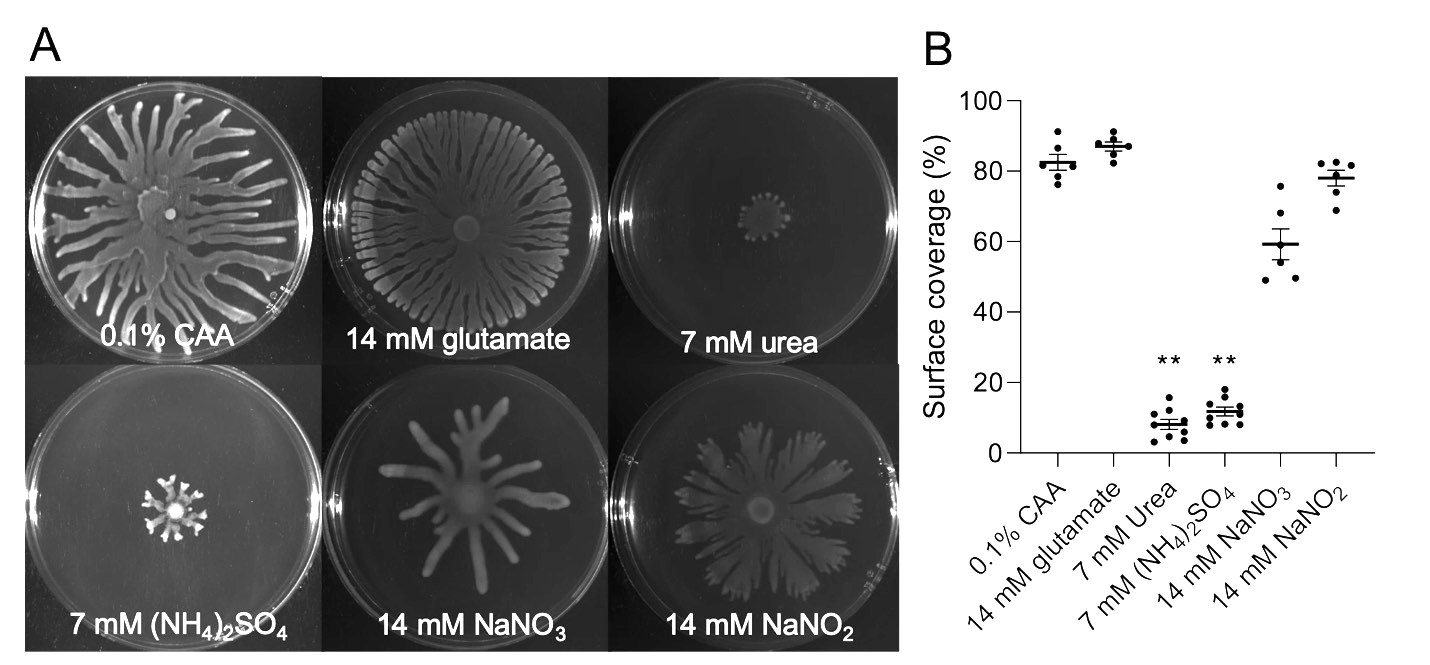


**Figure S2. Swarming motility of wild-type (WT) PA14 was influenced by nitrogen source.** (A) Representative images depict swarming motility of WT PA14 in the presence of different nitrogenous compounds. (B) Swarming motility was reduced by substituting casamino acids (CAA) for urea or ammonium sulfate ((NH_4_)_2_SO_4_) in swarm plates. Modified swarm plates were inoculated with 5 μl of planktonic cells suspended at an OD_600_ = 0.4-0.6 in basal medium (BM2) supplemented with nitrogen source as indicated and 0.4% glucose, then incubated for 18-24 h at 37ºC. Images captured using a BioRad ChemiDoc. Raw surface area coverage (%) of swarming colonies was assessed using ImageJ software. Data reported as mean ± standard error of the mean (SEM) from three independent experiments containing 2-3 biological replicates each (*n* = 6-9). ** *P* < 0.01 according to Kruskal-Wallis nonparametric test followed by Dunn’s post-hoc analysis.


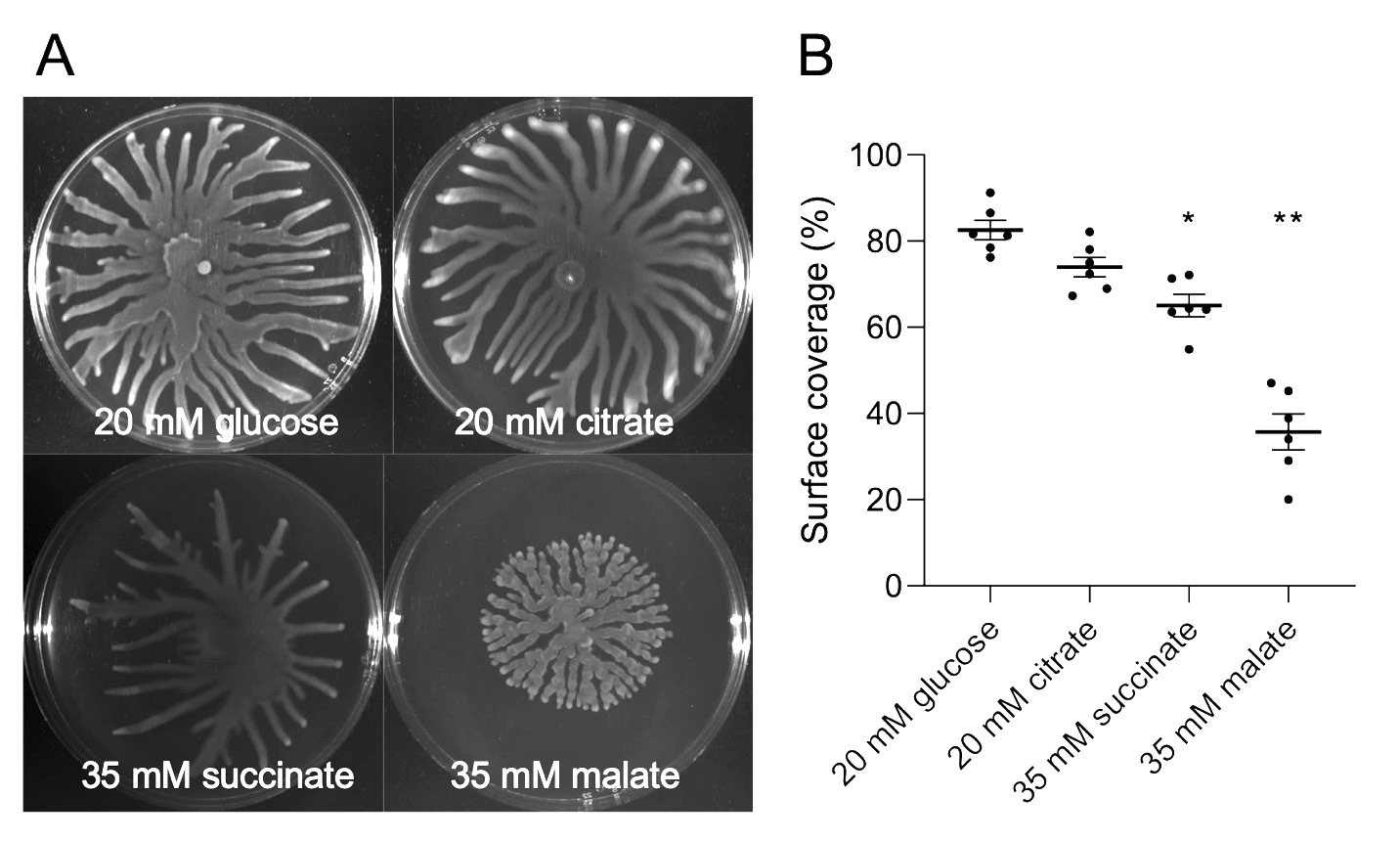


**Figure S3. Swarming motility of wild-type (WT) PA14 was influenced by carbon source.** (A) Representative images depict swarming motility of WT PA14 in the presence of different carbon-containing compounds. (B) Swarming motility was reduced by substituting glucose for succinate or malate in swarm plates. Modified swarm plates were inoculated with 5 μl of planktonic cells suspended at an OD_600_ = 0.4-0.6 in basal medium (BM2) supplemented with carbon source as indicated and 0.1% casamino acids (CAA), then incubated for 18-24 h at 37ºC. Images captured using a BioRad ChemiDoc. Raw surface area coverage (%) of swarming colonies was assessed using ImageJ software. Data reported as mean ± standard error of the mean (SEM) from three independent experiments containing 2 biological replicates each (*n* = 6). * *P* < 0.05, ** *P* < 0.01 according to Kruskal-Wallis nonparametric test followed by Dunn’s post-hoc analysis.

**Table S1. Primers used in this study.**

| **Primer** | **Sequence (5' - 3')** |
| --- | --- |
| **Knockout primers** | |
| *ntrB* outA | AGCTACTGCGCATCCTCTTC |
| *ntrB* up_fwd(BamHI) | AGAGGATCCTGCTCGACGGCAAGCCTG |
| *ntrB* up_rev | CGGTCTCTGATCGGCTCATGGGGCGGGCAGCTGTTCCAA |
| *ntrB* down_fwd | TTGGAACAGCTGCCCGCCCCATGAGCCGATCAGAGACCG |
| *ntrB* down_rev(HindIII) | TGCAAGCTTCGCCTCGCCGATGATCTC |
| *ntrB* outB | TCCATCAGGTCCTTGGGGAT |
| *ntrC* outA | ACGAGATCAAGAACCCGCTC |
| *ntrC* up_fwd(BamHI) | AGAGGATCCGAACCTGGCGCCGACCAACAT |
| *ntrC* up_rev | TCGCTCATGGGAGCAGTGGCGCTGGTCAATGCACTCCTTGTTCCAGGGGC |
| *ntrC* down_fwd | GCCCCTGGAACAAGGAGTGCATTGACCAGCGCCACTGCTCCCATGAGCGA |
| *ntrC* down_rev(HindIII) | TGCAAGCTTGGGCGAGCTGGTGATGAATGCCTC |
| *ntrC* outB | AAGGGGGAATTCTCAGCCTG |
| *ntrBC* up_rev | TCGCTCATGGGAGCAGTGGCGCTGGCGGGCAGCTGTTCCAA |
| *ntrBC* down_fwd | TTGGAACAGCTGCCCGCCAGCGCCACTGCTCCCATGAGCGA |
| **Complementation** | |
| *ntrB* fwd(EcoRI) | GCAGGAATTCATGCCGACCGATACCCTGCA |
| *ntrB* rev(BamHI) | GGGGGATCCTCAATGCACTCCTTGTTCCAGGGGC |
| *ntrC* fwd(EcoRI) | GAATTCGATAATGAGCCGATCAGAGACCGT |
| *ntrC* rev(BamHI) | GGGGGATCCTCAGTCGTCGCCTTCGT |
| **qRT-PCR** | |
| *ntrB* fwd | GATACCCTGCACCGACTGTT |
| *ntrB* rev | TGCGCCTGGAGTACATGA |
| *ntrC* fwd | ATCAGAGACCGTCTGGATCG |
| *ntrC* rev | TGCAGCAGGAAGGCATGA |
| *algR* fwd | TTCGGTCGCGAGGCTTTCTT |
| *algR* rev | TGACGAATTCGCCTGGAAG |
| *lasR* fwd | AGCTGGAACGCTCAAGTGGA |
| *lasR* rev | TGGCTGTCCTTAGGCAACAG |
| *rhlA* fwd | GGGTTGATCACCAAGGACGA |
| *rhlA* rev | AATCACCTGGTCTCCGCGT |
| *rhlB* fwd | CCTGTCGGCGTTTCATGGAA |
| *rhlB* rev | GAACGTTGTCATAGGGAGGG |

**Table S2. Growth rates of PA14 ntrBC mutant strains was influenced by nitrogen source and significantly reduced in the presence of nitrate or nitrite as well as casamino acids for the double mutant.** Briefly, bacteria were seeded from overnight cultures into batch cultures at low density (OD_600_ = 0.1) and incubated at 37ºC for 10 h with shaking in (A) basal medium (BM2) in which (NH_4_)_2_SO_4_ was replaced with (B) 0.1% casamino acids (CAA) (C) 14 mM NaNO_2_ or (D) 14 mM NaNO_3_. Growth rates were calculated by taking the slope of the curve during exponential growth. Mean growth rate ± standard error of the mean (SEM) from three independent experiments is shown (*n* = 3). * *P* < 0.05, ** *P* < 0.01 different from WT according to Welch’s t-test.

| **Medium** | **WT** | **Δ*ntrB*** | **Δ*ntrC*** | **Δ*ntrBC*** |
| --- | --- | --- | --- | --- |
| BM2 | 0.38 ± 0.06 | 0.36 ± 0.02 | 0.34 ± 0.08 | 0.09 ± 0.01* |
| BM2 (0.1% CAA) | 0.37 ± 0.08 | 0.39 ± 0.05 | 0.35 ± 0.07 | 0.09 ± 0.02* |
| BM2 (14 mM NaNO_2_) | 0.34 ± 0.05 | 0.07 ± 0.01** | 0.11 ± 0.01** | 0.04 ± 0.01** |
| BM2 (14 mM NaNO_3_) | 0.48 ± 0.08 | 0.03 ± 0.01** | 0.04 ± 0.02** | 0.01 ± 0.007** |

**Table S3. All genes differentially expressed under swarming conditions in PA14 *ntrB* and *ntrC* mutant strains.** Gene expression for mutants is expressed as fold-change (FC) relative to PA14 wild-type (WT). Briefly, swarm plates were inoculated with 5 µl of planktonic cells suspended at OD600 = 0.4-0.6 in basal medium (BM2) supplemented with 0.1% casamino acids and 0.4% glucose, then incubated for 18-24 h at 37°C. Swarming cells were harvested from the tip of the swarm tendrils and RNA was isolated using Qiagen RNEasy MiniPrep kit.

| **PA14 Locus** | **PAO1 Locus** | **Name** | **Annotation** | **FC Δ*ntrB*** | **FC Δ*ntrC*** |
| --- | --- | --- | --- | --- | --- |
| PA14_00080 | PA0007 | PA0007 | hypothetical protein | -1.95 | -2.36 |
| PA14_00100 | PA0009 | glyQ | glycyl-tRNA synthetase alpha chain | 1.66 |  |
| PA14_00470 | PA0038 | PA0038 | hypothetical protein | 1.72 | 1.83 |
| PA14_00560 | PA0044 | exoT | exoenzyme T | 4.92 | 9.39 |
| PA14_00600 | PA0048 | PA0048 | probable transcriptional regulator | -4.06 | -4.04 |
| PA14_00620 | PA0049 | PA0049 | hypothetical protein | -3.44 | -5.58 |
| PA14_00650 | PA0052 | PA0052 | hypothetical protein | -1.89 | -2.25 |
| PA14_00670 | PA0055 | PA0055 | hypothetical protein | 1.90 | 1.76 |
| PA14_00710 | PA0059 | osmC | osmotically inducible protein OsmC | 3.21 | 5.92 |
| PA14_00720 | PA0060 | PA0060 | conserved hypothetical protein | 2.45 | 4.19 |
| PA14_00740 | PA0062 | PA0062 | hypothetical protein | 2.99 | 5.07 |
| PA14_01250 | PA0103 | PA0103 | probable sulfate transporter | -2.80 | -2.69 |
| PA14_01270 | PA0104 | PA0104 | hypothetical protein | -1.95 | -1.67 |
| PA14_01290 | PA0105 | coxB | cytochrome c oxidase, subunit II | -1.75 | -2.03 |
| PA14_01330 | PA0109 | PA0109 | hypothetical protein | -1.72 | -1.91 |
| PA14_01340 | PA0110 | PA0110 | hypothetical protein | -2.10 | -2.30 |
| PA14_01360 | PA0112 | PA0112 | hypothetical protein | -2.05 | -2.61 |
| PA14_01410 | PA0116 | PA0116 | hypothetical protein | -1.76 | -1.94 |
| PA14_01580 | PA0129 | bauD | Amino acid permease | -2.82 | -2.58 |
| PA14_01600 | PA0130 | bauC | 3-Oxopropanoate dehydrogenase | -2.67 | -2.45 |
| PA14_01610 | PA0131 | bauB | BauB | -2.57 | -1.87 |
| PA14_01620 | PA0132 | bauA | Beta-alanine:pyruvate transaminase | -5.60 | -4.65 |
| PA14_01670 | PA0136 | PA0136 | probable ATP-binding component of ABC transporter | -36.6 | -37.6 |
| PA14_01680 | PA0137 | PA0137 | probable permease of ABC transporter | -112 | -154 |
| PA14_01690 | PA0138 | PA0138 | probable permease of ABC transporter | -22.2 | -29.7 |
| PA14_01750 | PA0142 | PA0142 | hypothetical protein | 1.74 |  |
| PA14_01770 | - | - | - | -11.3 | -11.5 |
| PA14_01780 | PA0144 | PA0144 | hypothetical protein | -9.12 | -9.86 |
| PA14_01800 | PA0146 | PA0146 | conserved hypothetical protein | -33.1 | -31.3 |
| PA14_01810 | PA0147 | PA0147 | probable oxidoreductase | -5.47 | -5.55 |
| PA14_01830 | PA0148 | PA0148 | adenine deaminase | -1.84 | -2.05 |
| PA14_01970 | PA0158 | triC | Resistance-Nodulation-Cell Division (RND) triclosan efflux transporter, TriC | 1.51 | 2.22 |
| PA14_02060 | PA0165 | PA0165 | hypothetical protein | -8.22 | -10.72 |
| PA14_02070 | PA0166 | PA0166 | probable transporter | -41.9 | -244 |
| PA14_02090 | PA0167 | PA0167 | probable transcriptional regulator | -1.57 | -1.64 |
| PA14_02100 | PA0168 | PA0168 | conserved hypothetical protein | -1.79 | -1.60 |
| PA14_02180 | PA0173 | PA0173 | probable methylesterase | -2.88 | -3.40 |
| PA14_02190 | PA0174 | PA0174 | conserved hypothetical protein | -2.43 | -2.67 |
| PA14_02200 | PA0175 | PA0175 | probable chemotaxis protein methyltransferase | -2.15 | -2.71 |
| PA14_02220 | PA0176 | aer2 | aerotaxis transducer Aer2 | -2.38 | -2.62 |
| PA14_02230 | PA0177 | PA0177 | probable purine-binding chemotaxis protein | -2.43 | -2.72 |
| PA14_02250 | PA0178 | PA0178 | probable two-component sensor | -2.69 | -3.02 |
| PA14_02260 | PA0179 | PA0179 | probable two-component response regulator | -2.03 | -2.55 |
| PA14_02270 | PA0180 | cttP | chemotactic transducer for trichloroethylene [positive chemotaxis], CttP | -1.70 | -1.80 |
| PA14_02450 | PA0195 | pntAA | putative NAD(P) transhydrogenase, subunit alpha part 1 | -1.87 | -2.88 |
| PA14_02450 | PA0195 | pntAB | putative NAD(P) transhydrogenase, subunit alpha part 2 | -1.87 | -2.88 |
| PA14_02470 | PA0196 | pntB | pyridine nucleotide transhydrogenase, beta subunit | -1.64 | -2.31 |
| PA14_02550 | PA0208 | mdcA | malonate decarboxylase alpha subunit | -10.3 | -9.26 |
| PA14_02560 | PA0209 | PA0209 | conserved hypothetical protein | -19.2 | -21.9 |
| PA14_02570 | PA0210 | mdcC | malonate decarboxylase delta subunit | -14.8 | -29.7 |
| PA14_02580 | PA0211 | mdcD | malonate decarboxylase beta subunit | -32.0 | -27.1 |
| PA14_02590 | PA0212 | mdcE | malonate decarboxylase gamma subunit | -14.0 | -9.63 |
| PA14_02610 | PA0213 | PA0213 | hypothetical protein | -13.7 | -9.25 |
| PA14_02620 | PA0214 | PA0214 | probable acyl transferase | -11.3 | -10.8 |
| PA14_02630 | PA0215 | PA0215 | malonate transporter MadL | -3.64 | -4.13 |
| PA14_02640 | PA0216 | PA0216 | malonate transporter MadM | -3.72 | -2.48 |
| PA14_02660 | PA0218 | PA0218 | probable transcriptional regulator | -2.71 | -2.51 |
| PA14_02680 | PA0219 | PA0219 | probable aldehyde dehydrogenase | -3.24 | -3.66 |
| PA14_02690 | PA0220 | PA0220 | amino acid APC family transporter | -7.50 | -6.26 |
| PA14_02700 | PA0221 | PA0221 | probable aminotransferase | -7.29 | -11.6 |
| PA14_02720 | PA0222 | PA0222 | hypothetical protein | -9.53 | -6.41 |
| PA14_03560 | PA0274 | PA0274 | hypothetical protein | -2.50 | -2.79 |
| PA14_03710 | PA0284 | PA0284 | hypothetical protein | 1.81 |  |
| PA14_03770 | PA0288 | gpuA | 3-guanidinopropionase | -2.70 | -3.28 |
| PA14_03800 | PA0291 | oprE | Anaerobically-induced outer membrane porin OprE precursor | 1.85 | 1.81 |
| PA14_03860 | PA0296 | spuI | Glutamylpolyamine synthetase | -2.57 | -2.56 |
| PA14_03870 | PA0297 | spuA | probable glutamine amidotransferase | -2.23 | -2.95 |
| PA14_03880 | PA0298 | spuB | Glutamylpolyamine synthetase | -1.87 | -1.73 |
| PA14_03900 | PA0299 | spuC | Polyamine:pyruvate transaminase | -3.33 | -3.69 |
| PA14_03920 | PA0300 | spuD | polyamine transport protein | -3.14 | -3.33 |
| PA14_03930 | PA0301 | spuE | polyamine transport protein | -3.30 | -3.27 |
| PA14_03940 | PA0302 | spuF | polyamine transport protein PotG | -3.46 | -3.49 |
| PA14_03950 | PA0303 | spuG | polyamine transport protein PotH | -3.23 | -3.45 |
| PA14_03960 | PA0304 | spuH | polyamine transport protein PotI | -4.04 | -3.47 |
| PA14_04010 | - | - | - | 1.63 |  |
| PA14_04190 | PA0321 | PA0321 | acetylpolyamine amidohydrolase | -3.45 | -3.43 |
| PA14_04210 | PA0322 | PA0322 | probable transporter | -3.04 | -3.18 |
| PA14_04220 | PA0323 | PA0323 | probable binding protein component of ABC transporter | -17.7 | -19.2 |
| PA14_04230 | PA0324 | PA0324 | probable permease of ABC transporter | -95.4 | -189 |
| PA14_04240 | PA0325 | PA0325 | probable permease of ABC transporter | -36.4 | -31.0 |
| PA14_04250 | PA0326 | PA0326 | probable ATP-binding component of ABC transporter | -15.6 | -25.6 |
| PA14_04290 | PA0328 | aaaA | arginine-specific autotransporter of Pseudomonas aeruginosa, AaaA | -3.14 | -3.13 |
| PA14_04300 | PA0329 | PA0329 | conserved hypothetical protein | 1.71 |  |
| PA14_04760 | PA0363 | coaD | phosphopantetheine adenylyltransferase | 1.61 | 1.59 |
| PA14_05430 | PA0417 | chpE | probable chemotaxis protein | -4.47 | -4.09 |
| PA14_05630 | PA0433 | PA0433 | hypothetical protein | 2.75 | 7.68 |
| PA14_05640 | PA0434 | PA0434 | hypothetical protein | 6.63 | 21.9 |
| PA14_05690 | PA0437 | codA | cytosine deaminase | -3.75 | -3.94 |
| PA14_05700 | PA0438 | codB | cytosine permease | -3.75 | -5.28 |
| PA14_05740 | PA0439 | PA0439 | probable oxidoreductase | -28.2 | -40.6 |
| PA14_05750 | PA0440 | PA0440 | probable oxidoreductase | -33.1 | -25.9 |
| PA14_05770 | PA0441 | dht | dihydropyrimidinase | -38.2 | -48.4 |
| PA14_05790 | PA0443 | PA0443 | probable transporter | -36.7 | -42.0 |
| PA14_05810 | PA0444 | PA0444 | N-carbamoyl-beta-alanine amidohydrolase | -27.8 | -23.5 |
| PA14_05920 | PA0454 | PA0454 | conserved hypothetical protein | -1.83 | -1.80 |
| PA14_06230 | PA0476 | PA0476 | probable permease | -14.0 | -13.5 |
| PA14_06310 | PA0484 | PA0484 | conserved hypothetical protein | -1.57 | -1.92 |
| PA14_06960 | PA0534 | pauB1 | FAD-dependent oxidoreductase | -2.83 | -2.29 |
| PA14_07040 | PA0542 | PA0542 | conserved hypothetical protein | 1.60 |  |
| PA14_07090 | PA0546 | metK | methionine adenosyltransferase | 2.23 | 2.15 |
| PA14_07170 | PA0551 | epd | D-erythrose 4-phosphate dehydrogenase | 1.78 | 2.01 |
| PA14_07190 | PA0552 | pgk | phosphoglycerate kinase | 1.57 | 2.19 |
| PA14_07260 | PA0558 | PA0558 | conserved hypothetical protein | 1.70 | 1.71 |
| PA14_07370 | PA0567 | PA0567 | conserved hypothetical protein | 2.50 | 3.12 |
| PA14_07770 | PA0595 | lptD | LPS-assembly protein LptD | 1.70 | 1.62 |
| PA14_07850 | PA0602 | PA0602 | probable binding protein component of ABC transporter | -1.98 | -1.95 |
| PA14_07860 | PA0603 | agtA | AgtA | 1.80 | 1.84 |
| PA14_08450 | PA0659 | PA0659 | hypothetical protein | 1.53 |  |
| PA14_08540 | PA0667 | PA0667 | conserved hypothetical protein | 1.83 | 1.87 |
| PA14_09220 | PA4230 | pchB | salicylate biosynthesis protein PchB | 1.84 | 1.90 |
| PA14_09260 | PA4227 | pchR | transcriptional regulator PchR | 1.72 | 1.70 |
| PA14_09440 | PA1903 | phzE2 | phenazine biosynthesis protein PhzE | 1.89 | 1.65 |
| PA14_09440 | PA4214 | phzE1 | phenazine biosynthesis protein PhzE | 1.89 | 1.65 |
| PA14_09550 | PA4204 | ppgL | periplasmic gluconolactonase, PpgL | 2.13 | 3.76 |
| PA14_09880 | PA4177 | PA4177 | hypothetical protein | -3.00 | -3.58 |
| PA14_09900 | PA4175 | piv | protease IV | -2.68 | -3.75 |
| PA14_09920 | PA4173 | PA4173 | conserved hypothetical protein | -6.18 | -4.84 |
| PA14_09930 | PA4172 | PA4172 | probable nuclease | 4.24 | 8.94 |
| PA14_09940 | PA4171 | PA4171 | probable protease | 4.64 | 9.02 |
| PA14_10180 | PA4158 | fepC | ferric enterobactin transport protein FepC | -5.65 |  |
| PA14_10220 | PA4154 | PA4154 | conserved hypothetical protein | 2.91 | 4.45 |
| PA14_10240 | PA4152 | PA4152 | probable hydrolase | -3.96 |  |
| PA14_10280 | PA4148 | PA4148 | probable short-chain dehydrogenase | -58.2 | -16.6 |
| PA14_10290 | PA4147 | acoR | transcriptional regulator AcoR | -2.15 | -2.46 |
| PA14_10340 | PA4143 | PA4143 | probable toxin transporter | 1.72 | 1.52 |
| PA14_10360 | PA4141 | PA4141 | hypothetical protein | 2.38 | 1.82 |
| PA14_10370 | PA4140 | PA4140 | hypothetical protein | 4.46 | 4.29 |
| PA14_10380 | PA4139 | PA4139 | hypothetical protein | 6.30 | 5.50 |
| PA14_10480 | PA4135 | PA4135 | probable transcriptional regulator | 1.66 |  |
| PA14_10730 | PA4115 | PA4115 | conserved hypothetical protein | -1.58 |  |
| PA14_10750 | PA4113 | PA4113 | probable major facilitator superfamily (MFS) transporter | 1.89 | 1.74 |
| PA14_10820 | PA4108 | PA4108 | cyclic di-GMP phosphodiesterase | -1.67 | -1.86 |
| PA14_11240 | PA4070 | PA4070 | probable transcriptional regulator | -3.74 | -3.21 |
| PA14_11310 | PA4064 | PA4064 | probable ATP-binding component of ABC transporter | 2.09 |  |
| PA14_11490 | PA4048 | PA4048 | hypothetical protein | -1.57 | -1.82 |
| PA14_11580 | PA4041 | PA4041 | hypothetical protein | -1.67 | -1.89 |
| PA14_11690 | PA4031 | ppa | inorganic pyrophosphatase | 1.59 |  |
| PA14_11730 | - | - | - | -29.1 | -28.4 |
| PA14_11740 | PA4027 | PA4027 | hypothetical protein | -1.81 | -2.02 |
| PA14_11750 | PA4026 | PA4026 | probable acetyltransferase | -2.12 | -2.43 |
| PA14_11760 | PA4025 | PA4025 | probable ethanolamine ammonia-lyase light chain | -5.97 | -6.32 |
| PA14_11770 | PA4024 | eutB | ethanolamine ammonia-lyase large subunit | -4.66 | -5.59 |
| PA14_11790 | PA4023 | PA4023 | probable transport protein | -11.9 | -16.5 |
| PA14_11810 | PA4022 | hdhA | hydrazone dehydrogenase, HdhA | -2.76 | -3.64 |
| PA14_11940 | PA4012 | PA4012 | hypothetical protein | -1.96 | -1.81 |
| PA14_12180 | PA3990 | PA3990 | conserved hypothetical protein | 1.78 |  |
| PA14_12260 | PA3986 | PA3986 | hypothetical protein | -1.70 | -2.15 |
| PA14_12270 | PA3985 | PA3985 | conserved hypothetical protein | -1.87 | -2.07 |
| PA14_12620 | PA3962 | PA3962 | hypothetical protein | 2.11 | 2.98 |
| PA14_12650 | PA3959 | PA3959 | hypothetical protein | 2.00 |  |
| PA14_12680 | PA3957 | PA3957 | probable short-chain dehydrogenase | -2.55 | -2.89 |
| PA14_13110 | PA3924 | PA3924 | probable medium-chain acyl-CoA ligase | -2.22 | -2.77 |
| PA14_13130 | PA3923 | PA3923 | hypothetical protein | -2.36 | -2.65 |
| PA14_13140 | PA3922 | PA3922 | conserved hypothetical protein | -2.53 | -2.75 |
| PA14_13170 | PA3920 | PA3920 | probable metal transporting P-type ATPase | -2.40 | -4.34 |
| PA14_13490 | PA3897 | PA3897 | hypothetical protein | -2.41 | -2.83 |
| PA14_14040 | PA3861 | rhl | ATP-dependent RNA helicase RhlB | 1.50 |  |
| PA14_14330 | PA3842 | spcS | specific Pseudomonas chaperone for ExoS, SpcS | 9.55 | 13.3 |
| PA14_14660 | PA3819 | PA3819 | conserved hypothetical protein | 1.76 | 3.13 |
| PA14_14680 | PA3818 | PA3818 | extragenic suppressor protein SuhB | 1.50 | 1.55 |
| PA14_14810 | PA3808 | PA3808 | conserved hypothetical protein | -1.58 | -1.56 |
| PA14_14820 | PA3807 | ndk | nucleoside diphosphate kinase | 1.62 |  |
| PA14_14990 | PA3795 | PA3795 | probable oxidoreductase | 2.66 | 4.14 |
| PA14_15070 | PA3790 | oprC | Putative copper transport outer membrane porin OprC precursor | 11.5 | 65.4 |
| PA14_15120 | PA3785 | PA3785 | conserved hypothetical protein | 2.96 | 4.42 |
| PA14_15130 | PA3784 | PA3784 | hypothetical protein | 1.80 | 3.39 |
| PA14_15290 | PA3771 | PA3771 | probable transcriptional regulator | -3.63 | -3.09 |
| PA14_15790 | PA3760 | PA3760 | N-Acetyl-D-Glucosamine phosphotransferase system transporter | -1.64 |  |
| PA14_15970 | PA3745 | rpsP | 30S ribosomal protein S16 | 1.74 |  |
| PA14_16110 | - | - | - | 1.80 | 2.48 |
| PA14_16390 | PA3710 | PA3710 | probable GMC-type oxidoreductase | -2.18 | -2.78 |
| PA14_16410 | PA3709 | PA3709 | probable major facilitator superfamily (MFS) transporter | -3.50 | -4.45 |
| PA14_16830 | PA3675 | PA3675 | hypothetical protein | 1.76 | 1.67 |
| PA14_16860 | PA3673 | plsB | glycerol-3-phosphate acyltransferase | 1.78 | 1.83 |
| PA14_16920 | PA3668 | PA3668 | conserved hypothetical protein | -1.94 | -1.91 |
| PA14_16970 | PA3664 | PA3664 | conserved hypothetical protein | 2.19 |  |
| PA14_17080 | PA3654 | pyrH | uridylate kinase | 1.61 |  |
| PA14_17150 | PA3648 | opr86 | outer membrane protein Opr86 | 1.81 | 1.68 |
| PA14_17170 | PA3647 | PA3647 | probable outer membrane protein precursor | 1.76 | 1.57 |
| PA14_17290 | PA3637 | pyrG | CTP synthase | 1.76 |  |
| PA14_17500 | PA3620 | mutS | DNA mismatch repair protein MutS | 1.50 |  |
| PA14_17930 | PA3584 | glpD | glycerol-3-phosphate dehydrogenase | -1.88 | -3.02 |
| PA14_17940 | PA3583 | glpR | glycerol-3-phosphate regulon repressor | -1.68 | -1.89 |
| PA14_17960 | PA3582 | glpK | glycerol kinase | -2.00 | -3.05 |
| PA14_17980 | PA3581 | glpF | glycerol uptake facilitator protein | -1.84 | -2.49 |
| PA14_18050 | PA3576 | PA3576 | hypothetical protein | -2.18 | -1.74 |
| PA14_18070 | - | - | - | -2.17 | -5.34 |
| PA14_18120 | PA3570 | mmsA | methylmalonate-semialdehyde dehydrogenase | -1.92 | -2.44 |
| PA14_18140 | PA3569 | mmsB | 3-hydroxyisobutyrate dehydrogenase | -2.54 | -2.94 |
| PA14_18150 | PA3568 | PA3568 | probable acetyl-coa synthetase | -3.14 | -2.07 |
| PA14_18250 | PA3562 | fruI | phosphotransferase system transporter enzyme I, FruI | -2.19 | -1.62 |
| PA14_18580 | PA3540 | algD | GDP-mannose 6-dehydrogenase AlgD | -6.52 | -4.38 |
| PA14_18690 | PA3529 | PA3529 | alkylhydroperoxide reductase C | 2.17 | 2.03 |
| PA14_18720 | PA3526 | motY | MotY | -2.04 | -1.62 |
| PA14_18760 | PA3523 | mexP | MexP | -5.51 | -5.27 |
| PA14_18780 | PA3522 | mexQ | MexQ | -2.73 | -3.34 |
| PA14_18790 | PA3521 | opmE | OpmE | -5.18 | -3.45 |
| PA14_18810 | PA3519 | PA3519 | hypothetical protein | -3.33 | -6.63 |
| PA14_18820 | PA3518 | PA3518 | hypothetical protein | -2.90 | -4.97 |
| PA14_18830 | PA3517 | PA3517 | probable lyase | -2.73 | -2.80 |
| PA14_18860 | PA3515 | PA3515 | hypothetical protein | -1.89 |  |
| PA14_18880 | PA3495 | nth | endonuclease III | 1.88 |  |
| PA14_19110 | PA3478 | rhlB | rhamnosyltransferase chain B | -2.18 | -1.98 |
| PA14_19170 | PA3472 | PA3472 | hypothetical protein | 1.79 | 1.84 |
| PA14_19490 | PA3450 | lsfA | 1-Cys peroxiredoxin LsfA | 2.10 | 2.29 |
| PA14_19650 | PA3436 | PA3436 | hypothetical protein | -144 | -294 |
| PA14_19660 | PA3435 | PA3435 | conserved hypothetical protein | 1.61 | 1.55 |
| PA14_19700 | PA3430 | PA3430 | probable aldolase | -1.79 | -1.56 |
| PA14_19710 | PA3429 | PA3429 | probable epoxide hydrolase | -2.44 | -1.96 |
| PA14_19850 | PA3420 | PA3420 | probable transcriptional regulator | -2.37 | -2.24 |
| PA14_19870 | PA3418 | ldh | leucine dehydrogenase | -1.54 | -2.16 |
| PA14_19900 | PA3417 | PA3417 | probable pyruvate dehydrogenase E1 component, alpha subunit | -2.39 | -2.53 |
| PA14_19910 | PA3416 | PA3416 | probable pyruvate dehydrogenase E1 component, beta chain | -2.28 | -2.55 |
| PA14_19920 | PA3415 | PA3415 | probable dihydrolipoamide acetyltransferase | -2.61 | -2.80 |
| PA14_20000 | PA3409 | hasS | HasS | -6.74 | -7.74 |
| PA14_20030 | PA3406 | hasD | transport protein HasD | -30.34 | -22.23 |
| PA14_20040 | PA3405 | hasE | metalloprotease secretion protein | -23.30 | -8.47 |
| PA14_20060 | - | - | - | -5.94 | -3.57 |
| PA14_20070 | PA3403 | PA3403 | hypothetical protein | -25.83 | -25.04 |
| PA14_20080 | PA3402 | PA3402 | hypothetical protein | -2.06 | -2.25 |
| PA14_20270 | PA3387 | rhlG | beta-ketoacyl reductase | -2.42 |  |
| PA14_20570 | PA3365 | PA3365 | probable chaperone | -3.32 | -3.07 |
| PA14_20580 | PA3364 | amiC | aliphatic amidase expression-regulating protein | -2.64 | -1.92 |
| PA14_20590 | PA3363 | amiR | aliphatic amidase regulator | -2.56 | -1.91 |
| PA14_20620 | PA3360 | PA3360 | probable secretion protein | -2.74 | -2.56 |
| PA14_20670 | PA3356 | pauA5 | Glutamylpolyamine synthetase | -1.51 |  |
| PA14_20690 | PA3354 | PA3354 | hypothetical protein | -1.92 | -1.69 |
| PA14_20740 | PA3350 | PA3350 | hypothetical protein | -2.11 | -2.23 |
| PA14_20760 | PA3348 | PA3348 | probable chemotaxis protein methyltransferase | -1.51 |  |
| PA14_20780 | PA3346 | hsbR | HptB-dependent secretion and biofilm regulator HsbR | -1.74 | -1.78 |
| PA14_20860 | PA3340 | PA3340 | hypothetical protein | -1.71 | -1.86 |
| PA14_20960 | PA3332 | PA3332 | conserved hypothetical protein | 1.92 |  |
| PA14_20970 | PA3331 | PA3331 | cytochrome P450 | 1.73 | 1.52 |
| PA14_21000 | PA3329 | PA3329 | hypothetical protein | 1.70 | - |
| PA14_21120 | PA3318 | PA3318 | hypothetical protein | 8.02 | 6.75 |
| PA14_21440 | PA3295 | PA3295 | probable HIT family protein | 1.78 |  |
| PA14_21550 | PA3285 | PA3285 | probable sigma-70 factor, ECF subfamily | -1.57 |  |
| PA14_21570 | PA3284 | PA3284 | hypothetical protein | -2.26 | -2.27 |
| PA14_21580 | PA3283 | PA3283 | conserved hypothetical protein | -3.60 | -2.77 |
| PA14_21680 | PA3273 | PA3273 | hypothetical protein | 1.62 | 3.39 |
| PA14_21820 | PA3262 | PA3262 | probable peptidyl-prolyl cis-trans isomerase, FkbP-type | 1.91 | 1.68 |
| PA14_21910 | PA3254 | PA3254 | probable ATP-binding component of ABC transporter | -2.17 | -2.60 |
| PA14_21920 | PA3253 | PA3253 | probable permease of ABC transporter | -2.33 | -3.66 |
| PA14_21930 | PA3252 | PA3252 | probable permease of ABC transporter | -2.60 | -3.23 |
| PA14_21940 | PA3251 | PA3251 | hypothetical protein | -2.64 | -3.29 |
| PA14_21960 | PA3250 | PA3250 | hypothetical protein | -2.22 | -3.11 |
| PA14_22010 | PA3245 | minE | cell division topological specificity factor MinE | 1.55 | 1.64 |
| PA14_22330 | PA3236 | betX | BetX | -2.96 | -2.77 |
| PA14_22480 | PA3224 | PA3224 | hypothetical protein | 1.62 |  |
| PA14_22570 | PA3221 | csaA | CsaA protein | 2.42 |  |
| PA14_22940 | PA3192 | gltR | two-component response regulator GltR | -1.59 |  |
| PA14_23060 | PA3184 | PA3184 | probable transcriptional regulator | -1.53 |  |
| PA14_23070 | PA3183 | zwf | glucose-6-phosphate 1-dehydrogenase | -1.55 | -1.52 |
| PA14_23080 | PA3182 | pgl | 6-phosphogluconolactonase | -1.80 | -1.63 |
| PA14_23200 | PA3173 | PA3173 | probable short-chain dehydrogenase | 1.65 |  |
| PA14_23500 | PA3139 | PA3139 | probable amino acid aminotransferase | 1.82 | 1.60 |
| PA14_24210 | PA3089 | PA3089 | hypothetical protein | -1.65 | -1.95 |
| PA14_24480 | PA3064 | pelA | PelA | 1.89 |  |
| PA14_24490 | PA3063 | pelB | PelB | 1.69 |  |
| PA14_24780 | PA3039 | PA3039 | probable transporter | -6.56 | -7.71 |
| PA14_24880 | PA3031 | PA3031 | hypothetical protein | 1.63 | 1.63 |
| PA14_25040 | PA3017 | PA3017 | conserved hypothetical protein | -3.70 | -3.69 |
| PA14_25090 | PA3013 | faoB | fatty-acid oxidation complex beta-subunit | 1.89 | 1.96 |
| PA14_25180 | PA3006 | psrA | transcriptional regulator PsrA | -2.63 | -2.82 |
| PA14_25430 | PA2988 | lolE | lipoprotein localization protein LolE | 1.66 | 1.92 |
| PA14_25440 | PA2987 | lolD | lipoprotein localization protein LolD | 1.63 | 2.12 |
| PA14_25450 | PA2986 | lolC | lipoprotein localization protein LolC | 2.34 |  |
| PA14_25520 | PA2980 | PA2980 | conserved hypothetical protein | 1.75 |  |
| PA14_26280 | PA2920 | PA2920 | probable chemotaxis transducer | -2.35 | -2.25 |
| PA14_26910 | PA2875 | PA2875 | conserved hypothetical protein | -1.61 | -1.65 |
| PA14_26940 | PA2872 | PA2872 | hypothetical protein | -1.83 | -1.62 |
| PA14_27100 | PA2862 | lipA | lactonizing lipase precursor | -2.06 |  |
| PA14_27120 | PA2860 | PA2860 | hypothetical protein | 1.72 |  |
| PA14_27210 | PA2851 | efp | translation elongation factor P | 1.60 |  |
| PA14_27630 | - | - | - | 9.78 | 8.53 |
| PA14_27640 | - | - | - | 10.5 | 10.8 |
| PA14_27650 | - | - | - | 7.08 | 4.91 |
| PA14_27660 | - | - | - | 6.64 | 4.93 |
| PA14_27675 | - | - | - | 3.31 | 3.67 |
| PA14_27680 | - | - | - | 4.06 | 4.58 |
| PA14_27690 | - | - | - | 6.92 |  |
| PA14_27710 | PA2817 | PA2817 | hypothetical protein | 1.56 | 1.75 |
| PA14_27830 | PA2808 | ptrA | Pseudomonas type III repressor A | -3.60 | -8.97 |
| PA14_27840 | PA2807 | PA2807 | hypothetical protein | -4.94 | -14.4 |
| PA14_28140 | PA2779 | PA2779 | hypothetical protein | -1.81 | -2.16 |
| PA14_28150 | PA2778 | PA2778 | hypothetical protein | -3.34 | -2.70 |
| PA14_28180 | PA2776 | pauB3 | FAD-dependent oxidoreductase | -1.86 | -1.77 |
| PA14_28220 | PA2773 | PA2773 | hypothetical protein | 2.52 | 4.51 |
| PA14_28250 | - | - | - | 1.82 | 1.51 |
| PA14_28280 | PA2770 | PA2770 | hypothetical protein | -1.62 | -1.57 |
| PA14_28490 | PA2754 | PA2754 | conserved hypothetical protein | 1.83 | 3.08 |
| PA14_28650 | PA2744 | thrS | threonyl-tRNA synthetase | 1.75 |  |
| PA14_29180 | PA2704 | PA2704 | probable transcriptional regulator | -3.85 | -3.15 |
| PA14_29240 | PA2698 | PA2698 | probable hydrolase | 1.75 | 1.60 |
| PA14_29420 | PA2682 | PA2682 | conserved hypothetical protein | -2.24 | -2.72 |
| PA14_29640 | PA2664 | fhp | flavohemoprotein | -6.23 | -5.77 |
| PA14_29650 | PA2663 | ppyR | psl and pyoverdine operon regulator, PpyR | -4.13 | -4.80 |
| PA14_29660 | PA2662 | PA2662 | conserved hypothetical protein | -4.98 | -4.39 |
| PA14_29710 | PA2659 | PA2659 | hypothetical protein | 2.73 | 1.87 |
| PA14_29760 | PA2654 | PA2654 | probable chemotaxis transducer | -2.20 | -1.93 |
| PA14_30240 | PA2619 | infA | initiation factor | 1.94 | 1.51 |
| PA14_30980 | - | - | - | 2.71 | 2.23 |
| PA14_31070 | - | - | - | -2.54 | -2.22 |
| PA14_31430 | - | - | - | 1.95 | 2.68 |
| PA14_31450 | - | - | - | -2.53 | -3.09 |
| PA14_32140 | PA2514 | antC | anthranilate dioxygenase reductase | -45.8 | -30.8 |
| PA14_32150 | PA2513 | antB | anthranilate dioxygenase small subunit | -94.5 | -100 |
| PA14_32160 | PA2512 | antA | anthranilate dioxygenase large subunit | -10.4 | -13.4 |
| PA14_32190 | PA2511 | antR | AntR | -7.68 | -8.12 |
| PA14_32220 | PA2509 | catB | muconate cycloisomerase I | -10.4 | -10.5 |
| PA14_32230 | PA2508 | catC | muconolactone delta-isomerase | -3.88 | -3.93 |
| PA14_32240 | PA2507 | catA | catechol 1,2-dioxygenase | -7.54 | -7.35 |
| PA14_32280 | PA2504 | PA2504 | hypothetical protein | -1.87 | -1.90 |
| PA14_32490 | PA2485 | PA2485 | hypothetical protein | 1.88 | 2.92 |
| PA14_32530 | PA2482 | PA2482 | probable cytochrome c | -2.44 | -2.60 |
| PA14_32540 | PA2481 | PA2481 | hypothetical protein | -3.21 | -2.31 |
| PA14_33030 | PA2443 | sdaA | L-serine dehydratase | -1.64 | -1.52 |
| PA14_33040 | PA2442 | gcvT2 | glycine cleavage system protein T2 | -1.62 |  |
| PA14_33050 | PA2441 | PA2441 | hypothetical protein | 2.78 | 1.68 |
| PA14_33060 | PA2440 | PA2440 | hypothetical protein | 1.95 | 1.66 |
| PA14_33160 | PA2433 | PA2433 | hypothetical protein | 2.80 | 5.81 |
| PA14_33250 | PA2427 | PA2427 | hypothetical protein | -4.68 | -3.77 |
| PA14_33270 | PA2425 | pvdG | PvdG | -4.02 | -7.59 |
| PA14_33280 | PA2424 | pvdL | PvdL | -4.29 | -6.11 |
| PA14_33450 | PA2416 | treA | periplasmic trehalase precursor | 2.67 | 5.83 |
| PA14_33460 | PA2415 | PA2415 | hypothetical protein | 3.05 | 4.55 |
| PA14_33530 | PA2410 | fpvF | FpvF | -2.49 | -4.03 |
| PA14_33540 | PA2409 | fpvE | FpvE | -8.13 | -6.50 |
| PA14_33550 | PA2408 | fpvD | FpvD | -4.64 | -6.59 |
| PA14_33560 | PA2407 | fpvC | FpvC | -3.41 | -5.75 |
| PA14_33570 | PA2406 | fpvK | FpvK | -6.44 | -8.01 |
| PA14_33590 | PA2404 | fpvH | FpvH | -5.47 | -5.86 |
| PA14_33600 | PA2403 | fpvG | FpvG | -2.65 | -4.23 |
| PA14_33610 | PA2402 | PA2402 | probable non-ribosomal peptide synthetase | -2.99 | -3.04 |
| PA14_33630 | PA2400 | pvdJ | PvdJ | -2.42 | -2.35 |
| PA14_33650 | PA2399 | pvdD | pyoverdine synthetase D | -2.09 | -2.00 |
| PA14_33680 | PA2398 | fpvA | ferripyoverdine receptor | -1.92 | -2.83 |
| PA14_33710 | PA2395 | pvdO | PvdO | -5.12 | -3.66 |
| PA14_33810 | PA2386 | pvdA | L-ornithine N5-oxygenase | -2.54 | -3.49 |
| PA14_33820 | PA2385 | pvdQ | 3-oxo-C12-homoserine lactone acylase PvdQ | -4.08 | -5.90 |
| PA14_33830 | PA2384 | PA2384 | hypothetical protein | -3.73 | -4.05 |
| PA14_33980 | - | - | - | -2.02 | -4.54 |
| PA14_34190 | PA2356 | msuD | methanesulfonate sulfonatase MsuD | -6.16 | -5.84 |
| PA14_34210 | PA2354 | PA2354 | probable transcriptional regulator | -3.33 | -3.14 |
| PA14_34540 | PA2326 | PA2326 | hypothetical protein | -3.53 | -2.46 |
| PA14_34820 | PA2304 | ambC | AmbC | 1.72 | 1.63 |
| PA14_34830 | PA2303 | ambD | AmbD | 1.89 | 1.73 |
| PA14_34870 | PA2300 | chiC | chitinase | 1.69 |  |
| PA14_35240 | PA2268 | PA2268 | hypothetical protein | -2.17 |  |
| PA14_35270 | PA2266 | PA2266 | probable cytochrome c precursor | -1.73 | -1.66 |
| PA14_35490 | PA2250 | lpdV | lipoamide dehydrogenase-Val | -1.62 |  |
| PA14_35840 | - | - | - | 1.55 |  |
| PA14_35880 | - | - | - | -1.79 |  |
| PA14_35940 | - | - | - | -4.37 | -2.89 |
| PA14_35950 | - | - | - | -4.48 | -2.45 |
| PA14_35970 | - | - | - | -4.39 | -2.78 |
| PA14_35990 | - | - | - | -4.02 | -2.70 |
| PA14_36200 | PA2204 | PA2204 | probable binding protein component of ABC transporter | 2.44 |  |
| PA14_36330 | PA2193 | hcnA | hydrogen cyanide synthase HcnA | 1.82 |  |
| PA14_36345 | PA2191 | exoY | adenylate cyclase ExoY | 3.03 | 4.52 |
| PA14_36360 | PA2187 | PA2187 | hypothetical protein | 2.29 | 5.32 |
| PA14_36375 | PA2180 | PA2180 | hypothetical protein | 2.76 | 6.46 |
| PA14_36480 | - | - | - | 3.95 | 9.15 |
| PA14_36490 | PA2173 | PA2173 | hypothetical protein | 3.04 | 10.2 |
| PA14_36530 | PA2169 | PA2169 | hypothetical protein | 3.06 | 5.16 |
| PA14_36570 | PA2165 | PA2165 | probable glycogen synthase | 2.39 | 5.33 |
| PA14_36670 | PA2157 | PA2157 | hypothetical protein | 2.09 | 5.71 |
| PA14_36690 | PA2155 | PA2155 | probable phospholipase | 1.72 | 4.98 |
| PA14_36710 | PA2153 | glgB | 1,4-alpha-glucan branching enzyme | 2.67 | 7.25 |
| PA14_36730 | PA2152 | PA2152 | probable trehalose synthase | 2.79 | 7.63 |
| PA14_36740 | PA2151 | PA2151 | conserved hypothetical protein | 2.71 | 6.66 |
| PA14_36790 | - | - | - | 3.46 | 7.68 |
| PA14_36810 | PA2147 | katE | catalase HPII | 6.56 | 16.2 |
| PA14_36820 | PA2146 | PA2146 | conserved hypothetical protein | 2.89 | 2.78 |
| PA14_36980 | PA2134 | PA2134 | hypothetical protein | 3.66 | 7.39 |
| PA14_37210 | PA2116 | PA2116 | conserved hypothetical protein | -2.91 | -3.39 |
| PA14_37220 | PA2115 | PA2115 | probable transcriptional regulator | -3.79 | -4.04 |
| PA14_37250 | PA2114 | PA2114 | probable major facilitator superfamily (MFS) transporter | -3.46 | -4.82 |
| PA14_37260 | PA2113 | opdO | pyroglutamate porin OpdO | -6.21 | -6.41 |
| PA14_37270 | PA2112 | PA2112 | conserved hypothetical protein | -6.17 | -6.24 |
| PA14_37290 | PA2111 | PA2111 | hypothetical protein | -4.61 | -5.95 |
| PA14_37310 | PA2110 | PA2110 | hypothetical protein | -8.42 | -6.73 |
| PA14_37350 | PA2107 | PA2107 | hypothetical protein | 2.29 | 4.99 |
| PA14_37660 | PA2076 | PA2076 | probable transcriptional regulator | -1.63 | -1.59 |
| PA14_37790 | PA2065 | pcoA | copper resistance protein A precursor | -2.13 | -3.96 |
| PA14_37810 | PA2064 | pcoB | copper resistance protein B precursor | -3.45 | -6.64 |
| PA14_37830 | PA2062 | PA2062 | probable pyridoxal-phosphate dependent enzyme | 1.67 | 1.79 |
| PA14_38050 | PA2046 | PA2046 | hypothetical protein | 2.09 | 3.63 |
| PA14_38160 | PA2039 | PA2039 | hypothetical protein | -1.99 | -2.43 |
| PA14_38200 | PA2035 | PA2035 | probable decarboxylase | -1.50 | -1.51 |
| PA14_38210 | PA2034 | PA2034 | hypothetical protein | -2.49 | - |
| PA14_38260 | PA2031 | PA2031 | hypothetical protein | -2.81 | -3.08 |
| PA14_38270 | PA2030 | PA2030 | hypothetical protein | -3.63 | -2.96 |
| PA14_38310 | PA2027 | PA2027 | hypothetical protein | -4.60 | -4.61 |
| PA14_38340 | PA2024 | PA2024 | probable ring-cleaving dioxygenase | -1.66 | -2.01 |
| PA14_38350 | PA2023 | galU | UTP--glucose-1-phosphate uridylyltransferase | 2.36 | 3.86 |
| PA14_38690 | PA1997 | PA1997 | probable AMP-binding enzyme | -1.84 | -1.61 |
| PA14_38740 | PA1992 | ercS | ErcS | -1.66 |  |
| PA14_38825 | PA1985 | pqqA | pyrroloquinoline quinone biosynthesis protein A | 2.17 | 1.66 |
| PA14_39070 | PA1969 | PA1969 | hypothetical protein | 1.74 | 2.11 |
| PA14_39090 | PA1967 | PA1967 | hypothetical protein | -2.34 | -2.75 |
| PA14_39240 | PA1954 | fapC | FapC | -2.75 | -3.66 |
| PA14_39280 | PA1950 | rbsK | ribokinase | -2.74 | -2.52 |
| PA14_39300 | PA1949 | rbsR | ribose operon repressor RbsR | -2.28 | -1.95 |
| PA14_39320 | PA1948 | rbsC | membrane protein component of ABC ribose transporter | -2.38 | -1.95 |
| PA14_39330 | PA1947 | rbsA | ribose transport protein RbsA | -11.01 | -11.11 |
| PA14_39520 | PA1933 | PA1933 | probable hydroxylase large subunit | 2.28 | 5.60 |
| PA14_39530 | PA1932 | PA1932 | probable hydroxylase molybdopterin-containing subunit | 2.07 | 4.87 |
| PA14_39660 | PA1921 | PA1921 | hypothetical protein | 3.90 | 7.38 |
| PA14_39880 | - | - | - | 2.09 | 1.67 |
| PA14_39945 | PA1901 | phzC2 | phenazine biosynthesis protein PhzC | 1.89 | 1.60 |
| PA14_39945 | PA4212 | phzC1 | phenazine biosynthesis protein PhzC | 1.89 | 1.60 |
| PA14_39960 | PA1900 | phzB2 | probable phenazine biosynthesis protein | 2.05 | 1.67 |
| PA14_39970 | PA1899 | phzA2 | probable phenazine biosynthesis protein | 2.19 | 2.00 |
| PA14_39980 | PA1898 | qscR | quorum-sensing control repressor | -3.43 | -3.40 |
| PA14_39990 | PA1897 | PA1897 | hypothetical protein | -6.91 | -7.42 |
| PA14_40010 | PA1896 | PA1896 | hypothetical protein | -7.13 | -6.78 |
| PA14_40020 | PA1895 | PA1895 | hypothetical protein | -6.30 | -5.31 |
| PA14_40030 | PA1894 | PA1894 | hypothetical protein | -4.49 | -5.42 |
| PA14_40040 | PA1893 | PA1893 | hypothetical protein | -5.86 | -5.78 |
| PA14_40050 | PA1892 | PA1892 | hypothetical protein | -5.56 | -5.11 |
| PA14_40060 | PA1891 | PA1891 | hypothetical protein | -7.63 | -6.31 |
| PA14_40080 | PA1889 | PA1889 | hypothetical protein | 1.90 | 2.71 |
| PA14_40110 | PA1887 | PA1887 | hypothetical protein | -2.19 | -2.33 |
| PA14_40250 | PA1875 | PA1875 | probable outer membrane protein precursor | -1.74 | -1.88 |
| PA14_40260 | PA1874 | PA1874 | hypothetical protein | -1.75 | -1.89 |
| PA14_40290 | PA1871 | lasA | LasA protease precursor | 2.22 | 2.45 |
| PA14_40300 | PA1870 | PA1870 | hypothetical protein | 1.92 | 3.38 |
| PA14_40770 | PA1838 | cysI | sulfite reductase | 1.72 | 1.54 |
| PA14_40820 | - | - | - | -2.04 |  |
| PA14_40850 | PA1831 | PA1831 | hypothetical protein | 1.65 |  |
| PA14_41090 | PA1812 | mltD | membrane-bound lytic murein transglycosylase D precursor | 2.00 | 1.90 |
| PA14_41110 | PA1811 | nppA1 | NppA1 | 1.70 |  |
| PA14_41480 | PA1786 | nasS | NasS | -91.2 | -121 |
| PA14_41490 | PA1785 | nasT | NasT | -83.4 | -92.8 |
| PA14_41500 | PA1784 | PA1784 | hypothetical protein | -2.38 | -3.35 |
| PA14_41510 | PA1783 | nasA | nitrate transporter | -162 | -651 |
| PA14_41520 | PA1782 | PA1782 | probable serine/threonine-protein kinase | -96.4 | -151 |
| PA14_41530 | PA1781 | nirB | assimilatory nitrite reductase large subunit | -50.4 | -137 |
| PA14_41540 | PA1780 | nirD | assimilatory nitrite reductase small subunit | -64.2 | -340 |
| PA14_41560 | PA1779 | PA1779 | assimilatory nitrate reductase | -39.9 | -90.0 |
| PA14_41563 | PA1778 | cobA | uroporphyrin-III C-methyltransferase | -25.4 | -36.8 |
| PA14_41690 | PA1768 | PA1768 | hypothetical protein | 2.15 |  |
| PA14_41730 | PA1766 | PA1766 | hypothetical protein | 1.54 | 1.55 |
| PA14_41780 | PA1762 | PA1762 | hypothetical protein | -2.06 | -2.14 |
| PA14_41790 | PA1761 | PA1761 | hypothetical protein | -1.93 | -1.97 |
| PA14_41800 | PA1760 | PA1760 | probable transcriptional regulator | -2.07 | -1.96 |
| PA14_41840 | PA1756 | cysH | 3'-phosphoadenosine-5'-phosphosulfate reductase | 1.72 | 1.59 |
| PA14_41920 | PA1750 | PA1750 | phospho-2-dehydro-3-deoxyheptonate aldolase | 1.59 |  |
| PA14_42100 | PA1735 | PA1735 | hypothetical protein | -3.82 | -2.85 |
| PA14_42130 | PA1733 | PA1733 | conserved hypothetical protein | -3.11 | -2.30 |
| PA14_42140 | PA1732 | PA1732 | conserved hypothetical protein | -4.20 | -2.38 |
| PA14_42150 | PA1731 | PA1731 | conserved hypothetical protein | -4.76 | -2.83 |
| PA14_42160 | PA1730 | PA1730 | conserved hypothetical protein | -5.29 | -3.25 |
| PA14_42180 | PA1729 | PA1729 | conserved hypothetical protein | -1.69 | -1.87 |
| PA14_42200 | PA1728 | PA1728 | hypothetical protein | -1.92 | -2.11 |
| PA14_42270 | PA1723 | pscJ | type III export protein PscJ | 2.51 | 4.39 |
| PA14_42280 | PA1722 | pscI | type III export protein PscI | 2.98 | 4.17 |
| PA14_42290 | PA1721 | pscH | type III export protein PscH | 3.24 | 4.79 |
| PA14_42300 | PA1720 | pscG | type III export protein PscG | 3.57 | 5.10 |
| PA14_42310 | PA1719 | pscF | type III export protein PscF | 5.39 | 5.91 |
| PA14_42320 | PA1718 | pscE | type III export protein PscE | 5.19 | 7.50 |
| PA14_42350 | PA1716 | pscC | Type III secretion outer membrane protein PscC precursor | 3.03 | 4.88 |
| PA14_42380 | PA1714 | exsD | ExsD | 2.54 | 4.04 |
| PA14_42390 | PA1713 | exsA | transcriptional regulator ExsA | 2.15 | 2.76 |
| PA14_42400 | PA1712 | exsB | exoenzyme S synthesis protein B | 2.10 | 2.37 |
| PA14_42440 | PA1709 | popD | Translocator outer membrane protein PopD precursor | 7.22 | 11.7 |
| PA14_42450 | PA1708 | popB | translocator protein PopB | 7.54 | 12.8 |
| PA14_42460 | PA1707 | pcrH | regulatory protein PcrH | 6.95 | 9.44 |
| PA14_42470 | PA1706 | pcrV | type III secretion protein PcrV | 4.48 | 7.72 |
| PA14_42480 | PA1705 | pcrG | regulator in type III secretion | 4.60 | 8.69 |
| PA14_42500 | PA1703 | pcrD | type III secretory apparatus protein PcrD | 2.70 | 3.75 |
| PA14_42510 | PA1702 | pcr4 | Pcr4 | 7.36 | 14.8 |
| PA14_42520 | PA1701 | pcr3 | Pcr3 | 6.22 | 11.7 |
| PA14_42550 | PA1698 | popN | Type III secretion outer membrane protein PopN precursor | 5.38 | 9.13 |
| PA14_42570 | PA1697 | PA1697 | ATP synthase in type III secretion system | 4.50 | 7.93 |
| PA14_42580 | PA1696 | pscO | translocation protein in type III secretion | 7.97 | 16.0 |
| PA14_42600 | PA1695 | pscP | translocation protein in type III secretion | 6.67 | 10.7 |
| PA14_42610 | PA1694 | pscQ | translocation protein in type III secretion | 7.22 | 12.0 |
| PA14_42620 | PA1693 | pscR | translocation protein in type III secretion | 12.58 | 20.71 |
| PA14_42640 | PA1691 | pscT | translocation protein in type III secretion | 5.68 | 9.16 |
| PA14_42660 | PA1690 | pscU | translocation protein in type III secretion | 3.45 | 6.19 |
| PA14_43110 | PA1655 | PA1655 | probable glutathione S-transferase | 1.69 |  |
| PA14_43130 | PA1654 | PA1654 | probable aminotransferase | 1.51 |  |
| PA14_43160 | PA1651 | PA1651 | probable transporter | -1.89 | -2.12 |
| PA14_43220 | PA1646 | PA1646 | probable chemotaxis transducer | -2.79 | -2.19 |
| PA14_43420 | PA1631 | PA1631 | probable acyl-CoA dehydrogenase | -1.68 | -1.54 |
| PA14_43570 | PA1620 | PA1620 | hypothetical protein | -2.42 | -3.17 |
| PA14_43580 | PA1619 | PA1619 | probable transcriptional regulator | -2.18 | -2.41 |
| PA14_43900 | - | - | - | 3.14 | 4.47 |
| PA14_44060 | PA1581 | sdhC | succinate dehydrogenase (C subunit) | 2.71 | 1.91 |
| PA14_44240 | PA1566 | pauA3 | Glutamylpolyamine synthetase | -42.6 | -42.0 |
| PA14_44260 | PA1565 | pauB2 | FAD-dependent oxidoreductase | -23.4 | -24.3 |
| PA14_44620 | PA1533 | PA1533 | conserved hypothetical protein | 1.91 | 2.19 |
| PA14_44710 | PA1524 | xdhA | xanthine dehydrogenase | -1.94 | -2.48 |
| PA14_44740 | PA1523 | xdhB | xanthine dehydrogenase | -1.73 | -2.01 |
| PA14_44760 | PA1522 | PA1522 | hypothetical protein | -1.76 | -1.87 |
| PA14_44850 | PA1515 | alc | allantoicase | -2.10 | -1.70 |
| PA14_44960 | PA1506 | PA1506 | hypothetical protein | -1.86 |  |
| PA14_45120 | PA1492 | PA1492 | hypothetical protein | -2.81 | -2.11 |
| PA14_45130 | PA1491 | PA1491 | probable transporter | -2.52 | -2.70 |
| PA14_45400 | PA1474 | PA1474 | hypothetical protein | -1.67 | -1.65 |
| PA14_45460 | PA1467 | PA1467 | hypothetical protein | -2.27 | -1.96 |
| PA14_45480 | PA1465 | PA1465 | hypothetical protein | -2.18 |  |
| PA14_45510 | PA1463 | PA1463 | hypothetical protein | -1.58 |  |
| PA14_45560 | PA1460 | motC | MotC | -1.92 | -1.69 |
| PA14_45580 | PA1459 | PA1459 | probable methyltransferase | -1.69 | -1.56 |
| PA14_45660 | PA1453 | flhF | flagellar biosynthesis protein FlhF | -2.00 | -1.58 |
| PA14_45780 | PA1445 | fliO | flagellar protein FliO | -1.70 | -1.58 |
| PA14_45830 | PA1441 | PA1441 | putative flagellar hook-length control protein FliK | -2.69 | -2.20 |
| PA14_46100 | PA1419 | PA1419 | probable transporter | -2.13 | -2.95 |
| PA14_46120 | PA1417 | PA1417 | probable decarboxylase | -2.32 | -2.44 |
| PA14_46140 | PA1416 | PA1416 | conserved hypothetical protein | -2.22 |  |
| PA14_46250 | PA1407 | PA1407 | hypothetical protein | 1.96 | 2.55 |
| PA14_46280 | PA1404 | PA1404 | hypothetical protein | 2.99 | 3.52 |
| PA14_46290 | PA1403 | PA1403 | probable transcriptional regulator | -1.90 |  |
| PA14_46750 | PA1356 | PA1356 | hypothetical protein | -1.76 | -2.14 |
| PA14_46810 | PA1351 | PA1351 | probable sigma-70 factor, ECF subfamily | -1.73 | -1.95 |
| PA14_46910 | PA1342 | aatJ | AatJ | -2.00 | -2.40 |
| PA14_46920 | PA1341 | aatQ | AatQ | -2.42 | -2.57 |
| PA14_46930 | PA1340 | aatM | AatM | -2.43 | -2.66 |
| PA14_46950 | PA1339 | aatP | AatP | -2.49 | -2.76 |
| PA14_46960 | PA1338 | ggt | gamma-glutamyltranspeptidase precursor | -3.05 | -3.18 |
| PA14_46970 | PA1337 | ansB | glutaminase-asparaginase | -2.21 | -2.95 |
| PA14_46980 | PA1336 | aauS | AauS | -1.78 | -1.82 |
| PA14_46990 | PA1335 | aauR | AauR | -2.20 | -1.97 |
| PA14_47090 | PA1327 | PA1327 | probable protease | -1.57 | -1.95 |
| PA14_47100 | PA1326 | ilvA2 | threonine dehydratase, biosynthetic | -3.84 | -3.45 |
| PA14_47150 | PA1321 | cyoE | cytochrome o ubiquinol oxidase protein CyoE | 2.92 | 3.96 |
| PA14_47160 | PA1320 | cyoD | cytochrome o ubiquinol oxidase subunit IV | 2.83 | 3.79 |
| PA14_47180 | PA1319 | cyoC | cytochrome o ubiquinol oxidase subunit III | 4.06 | 5.70 |
| PA14_47190 | PA1318 | cyoB | cytochrome o ubiquinol oxidase subunit I | 4.77 | 5.88 |
| PA14_47210 | PA1317 | cyoA | cytochrome o ubiquinol oxidase subunit II | 5.79 | 6.54 |
| PA14_47920 | PA1260 | lhpP | ABC transporter periplasmic-binding protein, LhpP | -8.56 | -9.82 |
| PA14_47930 | PA1259 | lhpH | LhpH | -16.9 | -9.67 |
| PA14_47970 | PA1255 | lhpK | D-hydroxyproline epimerase, LhpK | -3.90 | -2.74 |
| PA14_48000 | PA1254 | lhpC | delta1-pyrroline-4-hydroxy-2-carboxylate deaminase, LphC | -4.63 | -3.92 |
| PA14_48020 | PA1252 | dpkA | DpkA | -2.79 | -1.79 |
| PA14_48530 | PA1221 | PA1221 | hypothetical protein | 2.06 | 1.96 |
| PA14_48540 | PA1220 | PA1220 | hypothetical protein | 1.63 | 1.70 |
| PA14_48550 | PA1219 | PA1219 | hypothetical protein | 2.13 | 1.81 |
| PA14_48560 | PA1218 | PA1218 | hypothetical protein | 2.65 | 2.24 |
| PA14_48570 | PA1217 | PA1217 | probable 2-isopropylmalate synthase | 2.72 | 2.61 |
| PA14_48590 | PA1216 | PA1216 | hypothetical protein | 2.55 | 2.14 |
| PA14_48600 | PA1215 | PA1215 | hypothetical protein | 2.00 | 1.75 |
| PA14_48630 | PA1212 | PA1212 | probable major facilitator superfamily (MFS) transporter | 1.95 | 1.62 |
| PA14_48840 | PA1195 | ddaH | dimethylarginine dimethylaminohydrolase DdaH | -3.62 | -4.40 |
| PA14_48860 | PA1193 | PA1193 | hypothetical protein | 1.53 | 2.03 |
| PA14_48940 | PA0723 | coaB | coat protein B of bacteriophage Pf1 | -1.74 |  |
| PA14_48950 | PA0722 | PA0722 | hypothetical protein of bacteriophage Pf1 | 2.33 | 2.34 |
| PA14_49220 | PA1176 | napF | ferredoxin protein NapF | -2.22 | -2.29 |
| PA14_49300 | PA1169 | PA1169 | probable lipoxygenase | 3.91 | 3.47 |
| PA14_49310 | PA1168 | PA1168 | hypothetical protein | 4.39 | 2.98 |
| PA14_50080 | PA1105 | fliJ | flagellar protein FliJ | -1.79 |  |
| PA14_50100 | PA1104 | fliI | flagellum-specific ATP synthase FliI | -1.74 | -1.61 |
| PA14_50130 | PA1102 | fliG | flagellar motor switch protein FliG | -1.57 |  |
| PA14_50140 | PA1101 | fliF | Flagella M-ring outer membrane protein precursor | -2.55 | -1.97 |
| PA14_50250 | PA1095 | PA1095 | hypothetical protein | -1.70 |  |
| PA14_50300 | PA1091 | fgtA | flagellar glycosyl transferase, FgtA | -1.74 | -1.67 |
| PA14_50320 | PA1089 | PA1089 | conserved hypothetical protein | -1.87 | -1.77 |
| PA14_50330 | PA1088 | PA1088 | hypothetical protein | -2.11 | -1.87 |
| PA14_50340 | PA1087 | flgL | flagellar hook-associated protein type 3 FlgL | -1.84 | -1.66 |
| PA14_50360 | PA1086 | flgK | flagellar hook-associated protein 1 FlgK | -2.30 | -2.05 |
| PA14_50380 | PA1085 | flgJ | flagellar protein FlgJ | -2.45 | -2.08 |
| PA14_50410 | PA1084 | flgI | flagellar P-ring protein precursor FlgI | -2.99 | -2.26 |
| PA14_50420 | PA1083 | flgH | flagellar L-ring protein precursor FlgH | -2.35 | -1.94 |
| PA14_50430 | PA1082 | flgG | flagellar basal-body rod protein FlgG | -1.91 | -1.90 |
| PA14_50440 | PA1081 | flgF | flagellar basal-body rod protein FlgF | -2.71 | -2.05 |
| PA14_50520 | PA1074 | braC | branched-chain amino acid transport protein BraC | -2.17 | -2.32 |
| PA14_50530 | PA1073 | braD | branched-chain amino acid transport protein BraD | -2.92 | -2.94 |
| PA14_50540 | PA1072 | braE | branched-chain amino acid transport protein BraE | -3.02 | -3.40 |
| PA14_50550 | PA1071 | braF | branched-chain amino acid transport protein BraF | -3.34 | -3.58 |
| PA14_50560 | PA1070 | braG | branched-chain amino acid transport protein BraG | -3.73 | -3.74 |
| PA14_50640 | PA1063 | PA1063 | hypothetical protein | -2.25 |  |
| PA14_50670 | PA1060 | PA1060 | hypothetical protein | -2.14 | -1.97 |
| PA14_50860 | PA1043 | PA1043 | hypothetical protein | -1.51 | -1.64 |
| PA14_50880 | PA1041 | PA1041 | probable outer membrane protein precursor | -2.00 | -2.56 |
| PA14_50950 | PA1034 | PA1034 | hypothetical protein | 2.04 |  |
| PA14_51040 | PA1028 | amaA | L-Pipecolate oxidase | -2.57 | -2.70 |
| PA14_51170 | PA1017 | pauA | pimeloyl-CoA synthetase | -2.30 |  |
| PA14_51520 | - | - | - | 2.09 | 2.91 |
| PA14_51530 | - | - | - | 4.15 | 6.06 |
| PA14_51720 | PA0972 | tolB | TolB protein | 2.35 | 2.11 |
| PA14_51730 | PA0971 | tolA | TolA protein | 1.61 | 2.58 |
| PA14_51830 | PA0962 | dps | DNA-binding protein from starved cells, Dps | 2.19 | 2.58 |
| PA14_51840 | PA0961 | PA0961 | probable cold-shock protein | 1.85 | 1.78 |
| PA14_51850 | PA0960 | PA0960 | hypothetical protein | -1.65 | -1.66 |
| PA14_51860 | PA0959 | PA0959 | hypothetical protein | -1.74 | -1.97 |
| PA14_51880 | PA0958 | oprD | Basic amino acid, basic peptide and imipenem outer membrane porin OprD precursor | -4.06 | -5.67 |
| PA14_52050 | PA0944 | purN | phosphoribosylaminoimidazole synthetase | 1.72 |  |
| PA14_52120 | - | - | - | 2.11 | 2.25 |
| PA14_52290 | PA0925 | PA0925 | hypothetical protein | 1.97 | 1.88 |
| PA14_52350 | PA0920 | PA0920 | alanyl-phosphatidylglycerol synthase | 1.81 | 2.54 |
| PA14_52580 | PA0904 | lysC | aspartate kinase alpha and beta chain | 1.54 |  |
| PA14_52660 | PA0899 | aruB | N2-Succinylarginine dihydrolase | -1.54 | -1.57 |
| PA14_52840 | PA0884 | PA0884 | probable C4-dicarboxylate-binding periplasmic protein | -3.25 | -1.91 |
| PA14_52850 | PA0883 | PA0883 | probable acyl-CoA lyase beta chain | -14.9 | -5.11 |
| PA14_52870 | PA0882 | PA0882 | hypothetical protein | -6.94 | -4.42 |
| PA14_52880 | PA0881 | PA0881 | hypothetical protein | -8.73 | -3.06 |
| PA14_52900 | PA0879 | PA0879 | probable acyl-CoA dehydrogenase | -4.33 | -4.01 |
| PA14_53140 | PA0861 | rbdA | RbDA | -1.60 | -1.80 |
| PA14_53160 | PA0859 | PA0859 | hypothetical protein | -1.86 | -1.51 |
| PA14_53250 | PA0852 | cbpD | chitin-binding protein CbpD precursor | 2.27 | 1.89 |
| PA14_53250 | PA0852 | PA0852 | Uncharacterized protein | 2.27 | 1.89 |
| PA14_53400 | PA0840 | PA0840 | probable oxidoreductase | -1.98 |  |
| PA14_53770 | PA0812 | PA0812 | hypothetical protein | -2.02 | -2.04 |
| PA14_53790 | PA0810 | PA0810 | probable haloacid dehalogenase | -1.60 | -1.67 |
| PA14_54080 | PA0788 | PA0788 | hypothetical protein | -1.76 | -2.00 |
| PA14_54400 | PA0765 | mucC | positive regulator for alginate biosynthesis MucC | 1.76 | 2.47 |
| PA14_54630 | PA0746 | PA0746 | probable acyl-CoA dehydrogenase | -1.61 | -1.62 |
| PA14_54670 | PA0743 | PA0743 | probable 3-hydroxyisobutyrate dehydrogenase | -1.51 | -1.72 |
| PA14_54820 | PA0731 | PA0731 | hypothetical protein | 1.58 |  |
| PA14_54830 | PA0730 | PA0730 | probable transferase | -6.41 | -7.66 |
| PA14_55660 | PA4283 | recD | exodeoxyribonuclease V alpha chain | -1.70 |  |
| PA14_55760 | PA4291 | PA4291 | hypothetical protein | 1.72 |  |
| PA14_55790 | PA4294 | PA4294 | hypothetical protein | -1.71 | -1.98 |
| PA14_55840 | PA4298 | PA4298 | hypothetical protein | -2.59 | -2.97 |
| PA14_55850 | PA4299 | tadD | TadD | -2.22 | -2.38 |
| PA14_55880 | PA4301 | tadB | TadB | -2.66 | -2.54 |
| PA14_55890 | PA4302 | tadA | TadA ATPase | -2.35 | -2.45 |
| PA14_55900 | PA4303 | tadZ | TadZ | -2.48 | -2.95 |
| PA14_55920 | PA4304 | rcpA | RcpA | -2.36 | -2.71 |
| PA14_55930 | PA4305 | rcpC | RcpC | -2.58 | -2.65 |
| PA14_55960 | PA4307 | pctC | chemotactic transducer PctC | -1.63 | -1.95 |
| PA14_56000 | PA4309 | pctA | chemotactic transducer PctA | -1.61 |  |
| PA14_56300 | PA4333 | PA4333 | probable fumarase | 2.00 | 2.13 |
| PA14_56430 | PA4341 | PA4341 | probable transcriptional regulator | -10.8 | -8.71 |
| PA14_56450 | PA4342 | PA4342 | probable amidase | -1.85 |  |
| PA14_56470 | PA4343 | PA4343 | probable major facilitator superfamily (MFS) transporter | -3.35 |  |
| PA14_56770 | PA4365 | lysE | Lysine efflux permease | -5.08 | -3.99 |
| PA14_57060 | PA4390 | PA4390 | hypothetical protein | 2.51 | 4.35 |
| PA14_57110 | PA4394 | PA4394 | conserved hypothetical protein | 1.75 | 3.61 |
| PA14_57720 | PA4443 | cysD | ATP sulfurylase small subunit | 1.77 | 1.72 |
| PA14_57730 | PA4444 | mltB1 | soluble and membrane-bound lytic transglycosylases | 1.55 |  |
| PA14_58030 | PA4470 | fumC1 | fumarate hydratase | -2.84 | -2.31 |
| PA14_58150 | PA4481 | mreB | rod shape-determining protein MreB | 1.58 | 1.51 |
| PA14_58220 | PA4486 | PA4486 | conserved hypothetical protein | 1.86 | 1.81 |
| PA14_58350 | PA4496 | dppA1 | probable binding protein component of ABC transporter | -2.68 | -3.35 |
| PA14_58360 | PA4497 | dppA2 | probable binding protein component of ABC transporter | -2.87 | -3.65 |
| PA14_58410 | PA4501 | opdD | Glycine-glutamate dipeptide porin OpdP | -12.66 | -11.76 |
| PA14_58420 | PA4502 | dppA4 | probable binding protein component of ABC transporter | -6.69 | -6.87 |
| PA14_58440 | PA4503 | dppB | dipeptide ABC transporter permease DppB | -6.76 | -6.82 |
| PA14_58450 | PA4504 | dppC | dipeptide ABC transporter permease DppC | -6.22 | -6.49 |
| PA14_58470 | PA4505 | dppD | dipeptide ABC transporter ATP-binding protein DppD | -7.04 | -7.53 |
| PA14_58490 | PA4506 | dppF | dipeptide ABC transporter ATP-binding protein DppF | -7.64 | -7.67 |
| PA14_58500 | PA4507 | PA4507 | hypothetical protein | -2.76 | -2.17 |
| PA14_58510 | PA4508 | PA4508 | probable transcriptional regulator | -3.62 | -3.37 |
| PA14_58690 | PA4523 | PA4523 | hypothetical protein | -1.52 |  |
| PA14_59470 | - | - | - | 1.93 | 1.90 |
| PA14_60230 | PA4545 | comL | competence protein ComL | 1.69 | 1.60 |
| PA14_60400 | PA4563 | rpsT | 30S ribosomal protein S20 | 1.67 |  |
| PA14_60500 | PA4572 | fklB | peptidyl-prolyl cis-trans isomerase FklB | -1.96 | -2.11 |
| PA14_60570 | PA4578 | PA4578 | hypothetical protein | 1.63 |  |
| PA14_60630 | PA4582 | PA4582 | conserved hypothetical protein | -2.35 | -2.04 |
| PA14_60650 | PA4583 | PA4583 | conserved hypothetical protein | -2.00 | -1.82 |
| PA14_60710 | PA4588 | gdhA | glutamate dehydrogenase | 17.23 | 16.54 |
| PA14_60730 | PA4589 | PA4589 | probable outer membrane protein precursor | 1.85 | 1.69 |
| PA14_60750 | PA4590 | pra | protein activator | 2.52 | 2.01 |
| PA14_61290 | PA4632 | PA4632 | hypothetical protein | 1.56 |  |
| PA14_61300 | PA4633 | PA4633 | probable chemotaxis transducer | -1.97 | -1.84 |
| PA14_61390 | PA4639 | PA4639 | hypothetical protein | 1.93 | 2.30 |
| PA14_61880 | PA4678 | rimI | peptide n-acetyltransferase RimI | 1.68 | 1.57 |
| PA14_62240 | PA4702 | PA4702 | hypothetical protein | -2.00 | -2.04 |
| PA14_62250 | PA4703 | PA4703 | hypothetical protein | -1.55 | -1.82 |
| PA14_62300 | PA4708 | phuT | Heme-transport protein, PhuT | -1.77 |  |
| PA14_62350 | PA4710 | phuR | Heme/Hemoglobin uptake outer membrane receptor PhuR precursor | 1.60 | 2.16 |
| PA14_62600 | PA4731 | panD | aspartate 1-decarboxylase precursor | 1.97 | 1.67 |
| PA14_62930 | PA4758 | carA | carbamoyl-phosphate synthase small chain | 1.77 | - |
| PA14_63190 | PA4779 | PA4779 | hypothetical protein | -2.58 | -2.29 |
| PA14_63200 | PA4780 | PA4780 | conserved hypothetical protein | -2.81 | -2.89 |
| PA14_63210 | PA4781 | PA4781 | cyclic di-GMP phosphodiesterase | -1.84 | -1.57 |
| PA14_64270 | PA4858 | PA4858 | conserved hypothetical protein | -118 | -211 |
| PA14_64280 | PA4859 | PA4859 | probable permease of ABC transporter | -80.2 | -215 |
| PA14_64290 | PA4860 | PA4860 | probable permease of ABC transporter | -137 | -327 |
| PA14_64300 | PA4861 | PA4861 | probable ATP-binding component of ABC transporter | -78.2 | -248 |
| PA14_64310 | PA4862 | PA4862 | probable ATP-binding component of ABC transporter | -42.5 | -83.3 |
| PA14_64320 | PA4863 | PA4863 | hypothetical protein | -8.11 | -7.59 |
| PA14_64335 | PA4864 | ureD | urease accessory protein | -4.13 | -3.91 |
| PA14_64350 | PA4865 | ureA | urease gamma subunit | -3.37 | -3.72 |
| PA14_64360 | PA4866 | PA4866 | conserved hypothetical protein | -4.15 | -4.48 |
| PA14_64370 | PA4867 | ureB | urease beta subunit | -3.80 | -4.46 |
| PA14_64390 | PA4868 | ureC | urease alpha subunit | -3.60 | -3.80 |
| PA14_64480 | PA4876 | osmE | osmotically inducible lipoprotein OsmE | 1.92 | 3.69 |
| PA14_64520 | PA4880 | PA4880 | probable bacterioferritin | 2.96 | 6.44 |
| PA14_64650 | PA4891 | ureE | urease accessory protein UreE | -5.75 | -7.86 |
| PA14_64660 | PA4892 | ureF | urease accessory protein UreF | -7.79 | -6.70 |
| PA14_64670 | PA4893 | ureG | urease accessory protein UreG | -5.97 | -5.89 |
| PA14_64680 | PA4894 | PA4894 | hypothetical protein | -4.44 | -5.38 |
| PA14_64770 | PA4901 | mdlC | benzoylformate decarboxylase | -2.68 |  |
| PA14_64850 | PA4908 | PA4908 | hypothetical protein | -24.3 | -26.9 |
| PA14_64860 | PA4909 | PA4909 | probable ATP-binding component of ABC transporter | -20.3 | -20.1 |
| PA14_64870 | PA4910 | PA4910 | branched chain amino acid ABC transporter ATP binding protein | -27.3 | -28.5 |
| PA14_64880 | PA4911 | PA4911 | probable permease of ABC branched-chain amino acid transporter | -26.2 | -30.2 |
| PA14_64890 | PA4912 | PA4912 | branched chain amino acid ABC transporter membrane protein | -23.3 | -35.0 |
| PA14_64900 | PA4913 | PA4913 | probable binding protein component of ABC transporter | -9.74 | -10.6 |
| PA14_64910 | PA4914 | amaR | transcriptional regulator, AmaR | -1.85 | -2.12 |
| PA14_64920 | PA4915 | PA4915 | probable chemotaxis transducer | -1.72 | -2.04 |
| PA14_64930 | PA4916 | nrtR | Nudix-related transcriptional regulator NrtR | -1.66 |  |
| PA14_64950 | PA4918 | pcnA | nicotinamidase, PcnA | -2.99 | -2.74 |
| PA14_64990 | PA4921 | choE | cholinesterase, ChoE | -2.61 | -2.02 |
| PA14_65000 | PA4922 | azu | azurin precursor | 2.16 | 2.08 |
| PA14_65050 | PA4926 | PA4926 | conserved hypothetical protein | -3.12 | -3.10 |
| PA14_65060 | PA4927 | PA4927 | conserved hypothetical protein | -6.52 | -6.45 |
| PA14_65090 | PA4929 | PA4929 | hypothetical protein | -2.55 | -2.77 |
| PA14_65750 | PA4974 | PA4974 | probable outer membrane protein precursor | 1.53 |  |
| PA14_66340 | PA5019 | PA5019 | conserved hypothetical protein | -1.82 | -1.61 |
| PA14_66400 | PA5022 | PA5022 | conserved hypothetical protein | 1.79 | 1.67 |
| PA14_66510 | PA5030 | PA5030 | probable major facilitator superfamily (MFS) transporter | 5.88 | 5.91 |
| PA14_66580 | PA5037 | PA5037 | hypothetical protein | 1.54 | 1.54 |
| PA14_66690 | PA5047 | PA5047 | hypothetical protein | -1.53 |  |
| PA14_66820 | PA5056 | phaC1 | poly(3-hydroxyalkanoic acid) synthase 1 | -1.74 | -1.84 |
| PA14_66830 | PA5057 | phaD | poly(3-hydroxyalkanoic acid) depolymerase | -1.75 | -1.95 |
| PA14_66840 | PA5058 | phaC2 | poly(3-hydroxyalkanoic acid) synthase 2 | -2.06 | -2.61 |
| PA14_66850 | PA5059 | PA5059 | probable transcriptional regulator | -2.08 | -2.61 |
| PA14_67040 | PA5075 | PA5075 | probable permease of ABC transporter | -2.21 | -2.18 |
| PA14_67050 | PA5076 | PA5076 | probable binding protein component of ABC transporter | -1.94 | -2.07 |
| PA14_67065 | PA5077 | opgH | OpgH | 1.64 | 2.37 |
| PA14_67090 | PA5078 | opgG | OpgG | 1.95 | 2.57 |
| PA14_67240 | PA5091 | hutG | N-formylglutamate amidohydrolase | -2.33 | -2.66 |
| PA14_67250 | PA5092 | hutI | imidazolone-5-propionate hydrolase HutI | -2.90 | -2.77 |
| PA14_67260 | PA5093 | PA5093 | probable histidine/phenylalanine ammonia-lyase | -3.25 | -3.28 |
| PA14_67270 | PA5094 | PA5094 | probable ATP-binding component of ABC transporter | -2.69 | -2.62 |
| PA14_67280 | PA5095 | PA5095 | probable permease of ABC transporter | -4.34 | -4.63 |
| PA14_67300 | PA5096 | PA5096 | probable binding protein component of ABC transporter | -7.77 | -7.27 |
| PA14_67310 | PA5097 | PA5097 | probable amino acid permease | -6.02 | -6.21 |
| PA14_67320 | PA5098 | hutH | histidine ammonia-lyase | -6.38 | -7.01 |
| PA14_67340 | PA5099 | PA5099 | probable transporter | -5.05 | -4.06 |
| PA14_67350 | PA5100 | hutU | urocanase | -3.24 | -2.83 |
| PA14_67370 | PA5101 | PA5101 | hypothetical protein | -1.96 | -2.12 |
| PA14_67380 | PA5102 | PA5102 | hypothetical protein | -2.49 | -2.13 |
| PA14_67400 | PA5103 | puuR | PuuR | -1.72 | -2.28 |
| PA14_67510 | PA5112 | estA | esterase EstA | -1.70 | -1.88 |
| PA14_67670 | PA5124 | ntrB | two-component sensor NtrB | -254 |  |
| PA14_67720 | PA5128 | secB | secretion protein SecB | 2.03 | 1.62 |
| PA14_67770 | PA5131 | pgm | phosphoglycerate mutase | 1.75 | 2.31 |
| PA14_67840 | PA5137 | PA5137 | hypothetical protein | -2.67 | -2.82 |
| PA14_67850 | PA5138 | PA5138 | hypothetical protein | -2.96 | -3.25 |
| PA14_68060 | PA5152 | PA5152 | probable ATP-binding component of ABC transporter | -2.61 | -3.05 |
| PA14_68070 | PA5153 | PA5153 | amino acid (lysine/arginine/ornithine/histidine/octopine) ABC transporter periplasmic binding protein | -5.82 | -5.96 |
| PA14_68080 | PA5154 | PA5154 | probable permease of ABC transporter | -6.74 | -7.18 |
| PA14_68090 | PA5155 | PA5155 | amino acid (lysine/arginine/ornithine/histidine/octopine) ABC transporter membrane protein | -6.78 | -4.87 |
| PA14_68140 | PA5160 | PA5160 | drug efflux transporter | 1.83 | 1.91 |
| PA14_68280 | PA5168 | dctQ | DctQ | -2.85 | -2.05 |
| PA14_68290 | PA5169 | dctM | DctM | -2.18 | -2.54 |
| PA14_68400 | PA5178 | PA5178 | conserved hypothetical protein | 2.03 | 1.92 |
| PA14_68440 | PA5181 | PA5181 | probable oxidoreductase | -1.76 | -1.94 |
| PA14_68510 | PA5187 | PA5187 | probable acyl-CoA dehydrogenase | -2.08 | -2.22 |
| PA14_68530 | PA5188 | PA5188 | probable 3-hydroxyacyl-CoA dehydrogenase | -2.61 |  |
| PA14_68750 | PA5205 | PA5205 | conserved hypothetical protein | 1.65 |  |
| PA14_69330 | PA5251 | PA5251 | hypothetical protein | 1.69 | 1.77 |
| PA14_69660 | PA5276 | lppL | Lipopeptide LppL precursor | 3.57 |  |
| PA14_69795 | PA5287 | amtB | ammonium transporter AmtB | -15.7 | -19.2 |
| PA14_69810 | PA5288 | glnK | nitrogen regulatory protein P-II 2 | -2.68 | -2.51 |
| PA14_70100 | PA5309 | pauB4 | FAD-dependent oxidoreductase | -1.67 | -1.83 |
| PA14_70120 | PA5311 | PA5311 | probable major facilitator superfamily (MFS) transporter | -2.39 | -2.19 |
| PA14_70160 | PA5313 | gabT2 | Transaminase | -1.88 | -1.75 |
| PA14_70190 | PA5316 | rpmB | 50S ribosomal protein L28 | 1.81 |  |
| PA14_70450 | PA5337 | rpoZ | RNA polymerase omega subunit | 1.90 |  |
| PA14_70650 | PA5352 | PA5352 | conserved hypothetical protein | -29.0 | -14.1 |
| PA14_70670 | PA5353 | glcF | glycolate oxidase subunit GlcF | -15.6 | -20.0 |
| PA14_70680 | PA5354 | glcE | glycolate oxidase subunit GlcE | -25.5 | -16.5 |
| PA14_70690 | PA5355 | glcD | glycolate oxidase subunit GlcD | -9.09 | -8.42 |
| PA14_71000 | PA5376 | cbcV | CbcV | -1.66 | -1.66 |
| PA14_71070 | PA5380 | gbdR | GbdR | -9.34 | -9.10 |
| PA14_71180 | PA5390 | PA5390 | probable peptidic bond hydrolase | -1.94 |  |
| PA14_71240 | PA5396 | PA5396 | hypothetical protein | -2.57 | -2.70 |
| PA14_71250 | PA5397 | PA5397 | hypothetical protein | -6.10 | -6.03 |
| PA14_71260 | PA5398 | dgcA | DgcA, Dimethylglycine catabolism | -4.22 | -4.21 |
| PA14_71280 | PA5399 | dgcB | DgcB, Dimethylglycine catabolism | -2.66 | -2.96 |
| PA14_71300 | PA5400 | PA5400 | probable electron transfer flavoprotein alpha subunit | -4.16 | -2.66 |
| PA14_71410 | PA5410 | gbcA | GbcA | -6.64 | -6.33 |
| PA14_71420 | PA5411 | gbcB | GbcB | -2.77 | -2.74 |
| PA14_71460 | PA5415 | glyA1 | serine hydroxymethyltransferase | -4.76 | -4.96 |
| PA14_71470 | PA5416 | soxB | sarcosine oxidase beta subunit | -3.55 | -3.57 |
| PA14_71500 | PA5418 | soxA | sarcosine oxidase alpha subunit | -3.21 | -2.52 |
| PA14_71530 | PA5420 | purU2 | formyltetrahydrofolate deformylase | -4.55 | -4.11 |
| PA14_71880 | PA5444 | PA5444 | conserved hypothetical protein | -1.64 | -1.53 |
| PA14_71940 | PA5450 | wzt | ABC subunit of A-band LPS efflux transporter | -1.71 | -1.66 |
| PA14_71970 | PA5452 | wbpW | phosphomannose isomerase/GDP-mannose WbpW | -2.19 | -1.73 |
| PA14_71990 | PA5453 | gmd | GDP-mannose 4,6-dehydratase | -1.64 |  |
| PA14_72390 | PA5484 | kinB | KinB | 1.69 | 3.56 |
| PA14_72600 | PA5502 | PA5502 | hypothetical protein | 1.52 |  |
| PA14_72780 | PA5516 | pdxY | pyridoxamine kinase | 1.51 |  |
| PA14_72870 | PA5523 | PA5523 | probable aminotransferase | -2.81 | -1.97 |
| PA14_72890 | PA5525 | PA5525 | probable transcriptional regulator | -1.96 | -1.85 |
| PA14_72960 | PA5530 | PA5530 | C5-dicarboxylate transporter | 13.3 | 12.4 |
| PA14_73090 | PA5542 | PA5542 | Pseudomonas imipenem beta-lactamase PIB-1 | -2.22 | -1.90 |
| PA14_73100 | PA5543 | PA5543 | hypothetical protein | -3.39 | -2.21 |
| PA14_73110 | PA5544 | PA5544 | conserved hypothetical protein | -2.52 | -2.63 |
| PA14_73170 | PA5549 | glmS | glucosamine--fructose-6-phosphate aminotransferase | 4.51 | 2.59 |
| PA14_73190 | PA5550 | glmR | GlmR transcriptional regulator | 3.79 | 2.25 |
| PA14_00150 | PA0014 | PA0014 | hypothetical protein |  | 2.37 |
| PA14_00430 | PA0034 | PA0034 | probable two-component response regulator |  | 1.86 |
| PA14_00480 | PA0039 | PA0039 | hypothetical protein |  | 1.68 |
| PA14_01300 | PA0106 | coxA | cytochrome c oxidase, subunit I |  | -2.12 |
| PA14_01310 | PA0107 | PA0107 | conserved hypothetical protein |  | -2.30 |
| PA14_01320 | PA0108 | coIII | cytochrome c oxidase, subunit III |  | -2.11 |
| PA14_01350 | PA0111 | PA0111 | hypothetical protein |  | -2.58 |
| PA14_01380 | PA0113 | PA0113 | probable cytochrome c oxidase assembly factor |  | -2.56 |
| PA14_01840 | PA0149 | PA0149 | probable sigma-70 factor, ECF subfamily |  | -3.30 |
| PA14_01900 | PA0153 | pcaH | protocatechuate 3,4-dioxygenase, beta subunit |  | 1.85 |
| PA14_01940 | PA0156 | triA | Resistance-Nodulation-Cell Division (RND) triclosan efflux membrane fusion protein, TriA |  | 1.94 |
| PA14_01960 | PA0157 | triB | Resistance-Nodulation-Cell Division (RND) triclosan efflux membrane fusion protein, TriB |  | 1.85 |
| PA14_01980 | PA0159 | PA0159 | probable transcriptional regulator |  | 1.77 |
| PA14_01990 | PA0160 | PA0160 | hypothetical protein |  | 2.00 |
| PA14_02460 | PA0195.1 | - | - |  | -2.81 |
| PA14_02810 | PA0229 | pcaT | dicarboxylic acid transporter PcaT |  | 2.53 |
| PA14_02830 | PA0230 | pcaB | 3-carboxy-cis,cis-muconate cycloisomerase |  | 1.52 |
| PA14_02840 | PA0231 | pcaD | beta-ketoadipate enol-lactone hydrolase |  | 2.02 |
| PA14_02850 | PA0232 | pcaC | gamma-carboxymuconolactone decarboxylase |  | 2.20 |
| PA14_03090 | PA0250 | PA0250 | conserved hypothetical protein |  | -1.50 |
| PA14_03160 | PA0256 | PA0256 | hypothetical protein |  | -1.52 |
| PA14_03210 | PA0261 | PA0261 | hypothetical protein |  | -2.18 |
| PA14_03240 | PA0263 | hcpC | secreted protein Hcp |  | -2.16 |
| PA14_03240 | PA1512 | hcpA | secreted protein Hcp |  | -2.16 |
| PA14_03240 | PA5267 | hcpB | secreted protein Hcp |  | -2.16 |
| PA14_03670 | PA0281 | cysW | sulfate transport protein CysW |  | 1.93 |
| PA14_03700 | PA0283 | sbp | sulfate-binding protein precursor |  | 1.57 |
| PA14_03760 | PA0287 | gpuP | 3-guanidinopropionate transport protein |  | -4.00 |
| PA14_03855 | PA0295 | PA0295 | probable periplasmic polyamine binding protein |  | -1.61 |
| PA14_04040 | PA0309 | PA0309 | hypothetical protein |  | 2.07 |
| PA14_04330 | PA0332 | PA0332 | hypothetical protein |  | -1.59 |
| PA14_04340 | PA0333 | PA0333 | hypothetical protein |  | -1.83 |
| PA14_04510 | PA0344 | PA0344 | hypothetical protein |  | 1.72 |
| PA14_04640 | PA0354 | PA0354 | conserved hypothetical protein |  | 3.07 |
| PA14_04650 | PA0355 | pfpI | protease PfpI |  | 3.31 |
| PA14_04930 | PA0376 | rpoH | sigma factor RpoH |  | 1.61 |
| PA14_05020 | PA0384 | PA0384 | hypothetical protein |  | -1.50 |
| PA14_05050 | PA0387 | PA0387 | conserved hypothetical protein |  | -1.54 |
| PA14_05190 | PA0396 | pilU | twitching motility protein PilU |  | -1.55 |
| PA14_05600 | PA0431 | PA0431 | hypothetical protein |  | 1.55 |
| PA14_05620 | PA0432 | sahH | S-adenosyl-L-homocysteine hydrolase |  | 1.84 |
| PA14_05650 | PA0435 | PA0435 | hypothetical protein |  | 2.83 |
| PA14_05890 | PA0452 | PA0452 | probable stomatin-like protein |  | -1.73 |
| PA14_06010 | PA0460 | PA0460 | hypothetical protein |  | 1.74 |
| PA14_06060 | PA0463 | creB | two-component response regulator CreB |  | 1.55 |
| PA14_06300 | PA0483 | PA0483 | probable acetyltransferase |  | -1.55 |
| PA14_06390 | PA0490 | PA0490 | hypothetical protein |  | 1.75 |
| PA14_06460 | PA0495 | PA0495 | hypothetical protein |  | -1.64 |
| PA14_06530 | PA0502 | PA0502 | probable biotin biosynthesis protein bioH |  | -1.54 |
| PA14_06980 | PA0536 | PA0536 | hypothetical protein |  | 1.84 |
| PA14_06990 | PA0537 | PA0537 | conserved hypothetical protein |  | 1.77 |
| PA14_07110 | PA0547 | PA0547 | probable transcriptional regulator |  | 1.77 |
| PA14_07210 | PA0554 | PA0554 | hypothetical protein |  | 1.92 |
| PA14_07230 | PA0555 | fda | fructose-1,6-bisphosphate aldolase |  | 2.43 |
| PA14_07340 | PA0564 | PA0564 | probable transcriptional regulator |  | 1.74 |
| PA14_07480 | - | - | - |  | 1.70 |
| PA14_07500 | PA0575 | PA0575 | conserved hypothetical protein |  | -1.65 |
| PA14_07630 | PA0585 | PA0585 | hypothetical protein |  | -1.84 |
| PA14_07950 | PA0610 | prtN | transcriptional regulator PrtN |  | 1.64 |
| PA14_08380 | PA0653 | PA0653 | conserved hypothetical protein |  | 1.62 |
| PA14_08420 | PA0656 | PA0656 | probable HIT family protein |  | -1.52 |
| PA14_08695 | PA4276 | secE | secretion protein SecE |  | 1.66 |
| PA14_09160 | PA4235 | ftnA | bacterial ferritin |  | 1.62 |
| PA14_09180 | PA4234 | uvrA | excinuclease ABC subunit A |  | 1.51 |
| PA14_09200 | PA4232 | ssb | single-stranded DNA-binding protein |  | 1.52 |
| PA14_09210 | PA4231 | pchA | salicylate biosynthesis isochorismate synthase |  | 1.63 |
| PA14_09230 | PA4229 | pchC | pyochelin biosynthetic protein PchC |  | 1.68 |
| PA14_09240 | PA4228 | pchD | pyochelin biosynthesis protein PchD |  | 1.87 |
| PA14_09660 | PA4198 | PA4198 | probable AMP-binding enzyme |  | 1.88 |
| PA14_10490 | PA4134 | PA4134 | hypothetical protein |  | -5.84 |
| PA14_10500 | PA4133 | PA4133 | cytochrome c oxidase subunit (cbb3-type) |  | -5.50 |
| PA14_10560 | PA4129 | PA4129 | hypothetical protein |  | -1.53 |
| PA14_10770 | PA4112 | PA4112 | probable sensor/response regulator hybrid |  | -1.82 |
| PA14_11120 | PA4080 | PA4080 | probable response regulator |  | -1.51 |
| PA14_11900 | PA4016 | PA4016 | hypothetical protein |  | 1.86 |
| PA14_12080 | PA4001 | sltB1 | soluble lytic transglycosylase B |  | 1.64 |
| PA14_12160 | PA3992 | sltB3 | SltB3 |  | 1.63 |
| PA14_12610 | PA3963 | PA3963 | probable transporter |  | 2.86 |
| PA14_12710 | PA3954 | PA3954 | hypothetical protein |  | -1.66 |
| PA14_12840 | PA3945 | PA3945 | conserved hypothetical protein |  | -1.56 |
| PA14_13200 | - | - | - |  | -1.96 |
| PA14_13210 | - | - | - |  | -1.96 |
| PA14_13410 | PA3903 | prfC | peptide chain release factor 3 |  | 1.50 |
| PA14_13420 | PA3902 | PA3902 | hypothetical protein |  | 2.43 |
| PA14_13580 | PA3891 | opuCA | OpuC ABC transporter, ATP-binding protein, OpuCA |  | 3.81 |
| PA14_13590 | PA3890 | opuCB | OpuC ABC transporter, permease protein, OpuCB |  | 4.41 |
| PA14_13600 | PA3889 | opuCC | OpuC ABC transporter, periplasmic substrate-binding protein, OpuCC |  | 3.12 |
| PA14_13610 | PA3888 | opuCD | OpuC ABC transporter, permease protein, OpuCD |  | 2.84 |
| PA14_13620 | PA3887 | nhaP | Na+/H+ antiporter NhaP |  | -1.83 |
| PA14_13630 | - | - | - |  | 2.12 |
| PA14_14100 | PA3858 | PA3858 | probable amino acid-binding protein |  | -1.61 |
| PA14_14230 | PA3848 | PA3848 | hypothetical protein |  | -1.51 |
| PA14_14270 | PA3846 | PA3846 | hypothetical protein |  | -1.79 |
| PA14_15050 | PA3791 | PA3791 | hypothetical protein |  | 3.44 |
| PA14_15080 | PA3789 | PA3789 | hypothetical protein |  | 10.41 |
| PA14_15350 | - | - | - |  | 1.52 |
| PA14_15430 | PA3867 | PA3867 | probable DNA invertase |  | 1.59 |
| PA14_15770 | PA3762 | PA3762 | hypothetical protein |  | 1.64 |
| PA14_16020 | PA3740 | PA3740 | hypothetical protein |  | -1.62 |
| PA14_16350 | PA3714 | PA3714 | probable two-component response regulator |  | -1.52 |
| PA14_16630 | PA3692 | lptF | Lipotoxon F, LptF |  | 3.83 |
| PA14_16640 | PA3691 | PA3691 | hypothetical protein |  | 3.53 |
| PA14_16680 | PA3688 | PA3688 | hypothetical protein |  | -1.78 |
| PA14_17030 | PA3659 | PA3659 | probable aminotransferase |  | -1.61 |
| PA14_17320 | PA3635 | eno | enolase |  | 1.61 |
| PA14_17590 | PA3612 | PA3612 | conserved hypothetical protein |  | 2.17 |
| PA14_17600 | PA3611 | PA3611 | hypothetical protein |  | 2.93 |
| PA14_17730 | PA3598 | PA3598 | conserved hypothetical protein |  | 2.98 |
| PA14_18020 | PA3578 | PA3578 | conserved hypothetical protein |  | -1.68 |
| PA14_18510 | PA3544 | algE | Alginate production outer membrane protein AlgE precursor |  | -2.42 |
| PA14_18800 | PA3520 | PA3520 | hypothetical protein |  | -4.74 |
| PA14_19100 | PA3479 | rhlA | rhamnosyltransferase chain A |  | -1.73 |
| PA14_19205 | PA3470 | PA3470 | hypothetical protein |  | 1.51 |
| PA14_19350 | PA3461 | PA3461 | conserved hypothetical protein |  | 5.68 |
| PA14_19360 | PA3460 | PA3460 | probable acetyltransferase |  | 5.08 |
| PA14_19370 | PA3459 | PA3459 | probable glutamine amidotransferase |  | 3.42 |
| PA14_19740 | PA3426 | PA3426 | probable enoyl CoA-hydratase/isomerase |  | -1.61 |
| PA14_20020 | PA3407 | hasAp | heme acquisition protein HasAp |  | -3.30 |
| PA14_20280 | PA3386 | PA3386 | conserved hypothetical protein |  | -1.79 |
| PA14_20320 | PA3383 | PA3383 | binding protein component of ABC phosphonate transporter |  | 2.78 |
| PA14_20370 | PA3379 | PA3379 | conserved hypothetical protein |  | 3.77 |
| PA14_20560 | PA3366 | amiE | aliphatic amidase |  | -1.59 |
| PA14_21190 | PA3311 | nbdA | NbdA |  | -1.99 |
| PA14_21510 | PA3289 | PA3289 | hypothetical protein |  | -1.79 |
| PA14_21670 | PA3274 | PA3274 | hypothetical protein |  | 3.62 |
| PA14_21690 | PA3272 | PA3272 | probable ATP-dependent DNA helicase |  | 1.64 |
| PA14_21830 | - | - | - |  | 3.44 |
| PA14_21970 | PA3249 | PA3249 | probable transcriptional regulator |  | -2.07 |
| PA14_22020 | PA3244 | minD | cell division inhibitor MinD |  | 1.57 |
| PA14_22400 | PA3231 | PA3231 | hypothetical protein |  | 2.45 |
| PA14_22410 | PA3230 | PA3230 | conserved hypothetical protein |  | 1.99 |
| PA14_22450 | PA3227 | ppiA | peptidyl-prolyl cis-trans isomerase A |  | 1.91 |
| PA14_22880 | - | - | - |  | 2.29 |
| PA14_23360 | PA3160 | wzz | O-antigen chain length regulator |  | 2.13 |
| PA14_23370 | PA3148 | wbpI | UDP-N-acetylglucosamine 2-epimerase WbpI |  | 2.05 |
| PA14_23400 | - | - | - |  | 1.72 |
| PA14_23420 | - | - | - |  | 1.71 |
| PA14_23430 | - | - | - |  | 2.00 |
| PA14_23440 | - | - | - |  | 1.86 |
| PA14_23450 | PA3146 | wbpK | probable NAD-dependent epimerase/dehydratase WbpK |  | 1.95 |
| PA14_24140 | PA3094 | PA3094 | probable transcriptional regulator |  | 1.51 |
| PA14_24440 | PA3069 | PA3069 | hypothetical protein |  | 2.46 |
| PA14_24640 | PA3050 | pyrD | dihydroorotate dehydrogenase |  | 1.55 |
| PA14_24730 | PA3043 | PA3043 | conserved hypothetical protein |  | 1.92 |
| PA14_24740 | PA3042 | PA3042 | hypothetical protein |  | 2.44 |
| PA14_24760 | PA3041 | PA3041 | hypothetical protein |  | 2.38 |
| PA14_24770 | PA3040 | PA3040 | conserved hypothetical protein |  | 2.11 |
| PA14_24970 | PA3023 | PA3023 | conserved hypothetical protein |  | 1.83 |
| PA14_25080 | PA3014 | faoA | fatty-acid oxidation complex alpha-subunit |  | 1.75 |
| PA14_25250 | PA3001 | PA3001 | probable glyceraldehyde-3-phosphate dehydrogenase |  | 1.96 |
| PA14_25390 | PA2991 | sth | soluble pyridine nucleotide transhydrogenase |  | 1.54 |
| PA14_25610 | PA2972 | PA2972 | conserved hypothetical protein |  | 1.75 |
| PA14_25820 | PA2955 | PA2955 | hypothetical protein |  | -1.60 |
| PA14_25880 | PA2951 | etfA | electron transfer flavoprotein alpha-subunit |  | 1.67 |
| PA14_25920 | PA2948 | cobM | precorrin-3 methylase |  | 1.74 |
| PA14_25960 | PA2945 | PA2945 | conserved hypothetical protein |  | 1.54 |
| PA14_26070 | PA2936 | PA2936 | hypothetical protein |  | -3.10 |
| PA14_26080 | PA2935 | PA2935 | hypothetical protein |  | -3.04 |
| PA14_26600 | PA2896 | sbrI | SbrI |  | 2.14 |
| PA14_26610 | PA2895 | sbrR | SbrR |  | 1.76 |
| PA14_26770 | PA2884 | PA2884 | hypothetical protein |  | 1.63 |
| PA14_26780 | PA2883 | PA2883 | hypothetical protein |  | 1.51 |
| PA14_27220 | PA2850 | ohr | organic hydroperoxide resistance protein |  | -1.78 |
| PA14_27230 | PA2849 | ohrR | OhrR |  | -1.57 |
| PA14_27720 | PA2816 | PA2816 | hypothetical protein |  | 1.93 |
| PA14_27730 | PA2815 | PA2815 | probable acyl-CoA dehydrogenase |  | 3.61 |
| PA14_27810 | PA2809 | copR | two-component response regulator, CopR |  | -1.79 |
| PA14_27850 | PA2806 | PA2806 | conserved hypothetical protein |  | -1.85 |
| PA14_27930 | PA2799 | PA2799 | hypothetical protein |  | -1.60 |
| PA14_28030 | PA2790 | PA2790 | hypothetical protein |  | -1.64 |
| PA14_28060 | PA2787 | cpg2 | carboxypeptidase G2 precursor |  | -1.60 |
| PA14_28110 | PA2782 | bamI | biofilm-associated metzincin Inhibitor, BamI |  | -1.91 |
| PA14_28170 | PA2777 | PA2777 | conserved hypothetical protein |  | 1.55 |
| PA14_28240 | - | - | - |  | 3.28 |
| PA14_28260 | - | - | - |  | -1.79 |
| PA14_28370 | PA2763 | PA2763 | hypothetical protein |  | -1.87 |
| PA14_28380 | PA2762 | PA2762 | hypothetical protein |  | -1.69 |
| PA14_28450 | PA2755 | eco | ecotin precursor |  | 1.71 |
| PA14_28520 | - | - | - |  | 3.00 |
| PA14_28530 | PA2751 | PA2751 | conserved hypothetical protein |  | 2.57 |
| PA14_28750 | - | - | - |  | 2.71 |
| PA14_29120 | PA2708 | PA2708 | hypothetical protein |  | 2.42 |
| PA14_29150 | PA2706 | PA2706 | hypothetical protein |  | 1.85 |
| PA14_29160 | PA2705 | PA2705 | hypothetical protein |  | 1.62 |
| PA14_29280 | PA2694 | trx2 | Trx2 |  | 2.35 |
| PA14_29330 | - | - | - |  | -2.07 |
| PA14_29820 | PA2651 | PA2651 | conserved hypothetical protein |  | 2.12 |
| PA14_30050 | PA2634 | aceA | isocitrate lyase AceA |  | 2.10 |
| PA14_30820 | PA2573 | PA2573 | probable chemotaxis transducer |  | -1.91 |
| PA14_30830 | PA2572 | PA2572 | probable two-component response regulator |  | -1.74 |
| PA14_30840 | PA2571 | PA2571 | probable two-component sensor |  | -1.95 |
| PA14_30900 | - | - | - |  | 3.98 |
| PA14_30910 | - | - | - |  | 3.29 |
| PA14_31290 | PA2570 | lecA | LecA |  | -1.79 |
| PA14_31400 | PA2561 | ctpH | CtpH |  | 1.71 |
| PA14_31530 | PA2553 | PA2553 | probable acyl-CoA thiolase |  | -1.53 |
| PA14_31610 | PA2549 | PA2549 | conserved hypothetical protein |  | 1.72 |
| PA14_31760 | PA2536 | PA2536 | probable phosphatidate cytidylyltransferase |  | -1.61 |
| PA14_31870 | PA2528 | muxA | MuxA |  | 2.19 |
| PA14_31890 | PA2527 | muxB | MuxB |  | 1.95 |
| PA14_31900 | PA2526 | muxC | MuxC |  | 1.78 |
| PA14_31920 | PA2525 | opmB | OpmB |  | 2.32 |
| PA14_32480 | PA2486 | ptrC | Pseudomonas type III repressor gene C, PtrC |  | 2.97 |
| PA14_32630 | PA2475 | PA2475 | probable cytochrome P450 |  | -1.88 |
| PA14_33480 | PA2414 | PA2414 | L-sorbosone dehydrogenase |  | 4.55 |
| PA14_33500 | PA2413 | pvdH | L-2,4-diaminobutyrate:2-ketoglutarate 4-aminotransferase, PvdH |  | 2.69 |
| PA14_33580 | PA2405 | fpvJ | FpvJ |  | -9.38 |
| PA14_33690 | PA2397 | pvdE | pyoverdine biosynthesis protein PvdE |  | -3.35 |
| PA14_33720 | PA2394 | pvdN | PvdN |  | -3.97 |
| PA14_33730 | PA2393 | PA2393 | putative dipeptidase |  | -2.85 |
| PA14_33740 | PA2392 | pvdP | PvdP |  | -2.38 |
| PA14_33930 | PA2375 | PA2375 | hypothetical protein |  | -1.53 |
| PA14_33940 | PA2374 | tseF | TseF |  | -1.94 |
| PA14_33960 | PA2373 | vgrG3 | VgrG3 |  | -2.19 |
| PA14_33970 | - | - | - |  | -2.41 |
| PA14_33990 | PA2371 | clpV3 | ClpV3 |  | -2.73 |
| PA14_34000 | PA2370 | hsiH3 | HsiH3 |  | -2.64 |
| PA14_34010 | PA2369 | hsiG3 | HsiG3 |  | -2.80 |
| PA14_34020 | PA2368 | hsiF3 | HsiF3 |  | -2.48 |
| PA14_34030 | PA2367 | hcp3 | Hcp3 |  | -2.08 |
| PA14_34050 | PA2366 | hsiC3 | HsiC3 |  | -2.71 |
| PA14_34070 | PA2365 | hsiB3 | HsiB3 |  | -2.77 |
| PA14_34080 | PA2364 | lip3 | Lip3 |  | -2.24 |
| PA14_34100 | PA2363 | hsiJ3 | HsiJ3 |  | -1.95 |
| PA14_34110 | PA2362 | dotU3 | DotU3 |  | -2.60 |
| PA14_34130 | PA2361 | icmF3 | IcmF3 |  | -2.48 |
| PA14_34140 | PA2360 | hsiA3 | hypothetical protein |  | -2.22 |
| PA14_34180 | PA2357 | msuE | NADH-dependent FMN reductase MsuE |  | -5.45 |
| PA14_34200 | PA2355 | PA2355 | probable FMNH2-dependent monooxygenase |  | -3.01 |
| PA14_34610 | - | - | - |  | 1.66 |
| PA14_34960 | PA2291 | PA2291 | probable glucose-sensitive porin |  | -1.71 |
| PA14_34970 | PA2290 | gcd | glucose dehydrogenase |  | -1.62 |
| PA14_35070 | PA2281 | PA2281 | probable transcriptional regulator |  | 1.90 |
| PA14_35190 | PA2272 | pbpC | penicillin-binding protein 3A |  | 1.51 |
| PA14_35550 | PA2245 | pslO | hypothetical protein |  | 2.64 |
| PA14_35570 | PA2244 | pslN | hypothetical protein |  | 3.28 |
| PA14_35640 | PA2239 | pslI | PslI |  | -1.94 |
| PA14_35650 | PA2238 | pslH | PslH |  | -1.88 |
| PA14_35670 | PA2237 | pslG | PslG |  | -1.72 |
| PA14_35900 | - | - | - |  | -1.65 |
| PA14_36350 | PA2189 | PA2189 | hypothetical protein |  | 4.61 |
| PA14_36370 | PA2181 | PA2181 | hypothetical protein |  | 3.09 |
| PA14_36390 | PA2179 | PA2179 | hypothetical protein |  | 5.44 |
| PA14_36400 | - | - | - |  | 2.10 |
| PA14_36410 | PA2178 | PA2178 | hypothetical protein |  | 4.73 |
| PA14_36420 | PA2177 | PA2177 | probable sensor/response regulator hybrid |  | 1.78 |
| PA14_36450 | PA2176 | PA2176 | hypothetical protein |  | 3.66 |
| PA14_36460 | PA2175 | PA2175 | hypothetical protein |  | 3.14 |
| PA14_36500 | PA2172 | PA2172 | hypothetical protein |  | 8.48 |
| PA14_36520 | PA2171 | PA2171 | hypothetical protein |  | 6.16 |
| PA14_36540 | PA2168 | PA2168 | hypothetical protein |  | 3.08 |
| PA14_36550 | PA2167 | PA2167 | hypothetical protein |  | 3.37 |
| PA14_36580 | PA2164 | PA2164 | probable glycosyl hydrolase |  | 4.86 |
| PA14_36590 | PA2163 | PA2163 | hypothetical protein |  | 4.69 |
| PA14_36605 | PA2162 | PA2162 | probable glycosyl hydrolase |  | 4.84 |
| PA14_36620 | PA2161 | PA2161 | hypothetical protein |  | 7.01 |
| PA14_36630 | PA2160 | PA2160 | probable glycosyl hydrolase |  | 6.59 |
| PA14_36650 | PA2159 | PA2159 | conserved hypothetical protein |  | 5.85 |
| PA14_36660 | PA2158 | PA2158 | probable alcohol dehydrogenase (Zn-dependent) |  | 6.59 |
| PA14_36680 | PA2156 | PA2156 | conserved hypothetical protein |  | 5.95 |
| PA14_36700 | PA2154 | PA2154 | conserved hypothetical protein |  | 4.63 |
| PA14_36760 | PA2150 | PA2150 | conserved hypothetical protein |  | 5.96 |
| PA14_36770 | PA2149 | PA2149 | hypothetical protein |  | 5.91 |
| PA14_36780 | PA2148 | PA2148 | conserved hypothetical protein |  | 5.22 |
| PA14_36830 | PA2145 | PA2145 | hypothetical protein |  | 3.34 |
| PA14_36840 | PA2144 | glgP | glycogen phosphorylase |  | 5.05 |
| PA14_36850 | PA2143 | PA2143 | hypothetical protein |  | 3.36 |
| PA14_36860 | - | - | - |  | -1.91 |
| PA14_36870 | PA2142 | PA2142 | probable short-chain dehydrogenase |  | 6.53 |
| PA14_36880 | PA2141 | PA2141 | hypothetical protein |  | 5.41 |
| PA14_36890 | PA2140 | PA2140 | probable metallothionein |  | 4.06 |
| PA14_36900 | - | - | - |  | 3.92 |
| PA14_36930 | PA2136 | PA2136 | hypothetical protein |  | 3.65 |
| PA14_36940 | - | - | - |  | 3.51 |
| PA14_36960 | PA2135 | PA2135 | probable transporter |  | 4.90 |
| PA14_37130 | PA2122 | PA2122 | hypothetical protein |  | -2.62 |
| PA14_37320 | PA2109 | PA2109 | hypothetical protein |  | 1.99 |
| PA14_37340 | PA2108 | PA2108 | probable decarboxylase |  | 4.65 |
| PA14_37380 | PA2097 | PA2097 | probable flavin-binding monooxygenase |  | -1.79 |
| PA14_37680 | PA2075 | PA2075 | hypothetical protein |  | -1.84 |
| PA14_37690 | PA2072 | PA2072 | conserved hypothetical protein |  | -1.55 |
| PA14_38080 | PA2044 | PA2044 | hypothetical protein |  | 1.64 |
| PA14_38370 | PA2021 | PA2021 | hypothetical protein |  | 2.82 |
| PA14_38770 | PA1990 | pqqH | PqqH |  | 1.55 |
| PA14_38780 | PA1989 | pqqE | pyrroloquinoline quinone biosynthesis protein E |  | 2.04 |
| PA14_38790 | PA1988 | pqqD | pyrroloquinoline quinone biosynthesis protein D |  | 1.77 |
| PA14_38800 | PA1987 | pqqC | pyrroloquinoline quinone biosynthesis protein C |  | 1.97 |
| PA14_38820 | PA1986 | pqqB | pyrroloquinoline quinone biosynthesis protein B |  | 2.54 |
| PA14_39190 | PA1959 | bacA | bacitracin resistance protein |  | 1.53 |
| PA14_39270 | PA1951 | fapF | FapF |  | -1.71 |
| PA14_39350 | PA1946 | rbsB | binding protein component precursor of ABC ribose transporter |  | -1.70 |
| PA14_39540 | PA1931 | PA1931 | probable ferredoxin |  | 1.59 |
| PA14_39560 | PA1930 | PA1930 | probable chemotaxis transducer |  | -1.65 |
| PA14_39780 | PA1914 | PA1914 | conserved hypothetical protein |  | -2.01 |
| PA14_39790 | PA1913 | PA1913 | hypothetical protein |  | -1.88 |
| PA14_40100 | PA1888 | PA1888 | hypothetical protein |  | -1.81 |
| PA14_40200 | PA1880 | PA1880 | probable oxidoreductase |  | -1.54 |
| PA14_40240 | PA1876 | PA1876 | probable ATP-binding/permease fusion ABC transporter |  | -1.85 |
| PA14_40380 | PA1864 | PA1864 | probable transcriptional regulator |  | -2.71 |
| PA14_40430 | PA1860 | PA1860 | hypothetical protein |  | -1.76 |
| PA14_40490 | PA1857 | PA1857 | conserved hypothetical protein |  | 1.63 |
| PA14_40570 | PA1851 | PA1851 | hypothetical protein |  | -1.54 |
| PA14_40750 | - | - | - |  | -2.10 |
| PA14_40840 | PA1832 | PA1832 | probable protease |  | 1.64 |
| PA14_41300 | - | - | - |  | 2.17 |
| PA14_41470 | PA1787 | acnB | aconitate hydratase 2 |  | 1.77 |
| PA14_41575 | PA1776 | sigX | ECF sigma factor SigX |  | 1.60 |
| PA14_41650 | PA1771 | estX | EstX |  | 1.61 |
| PA14_41710 | PA1767 | PA1767 | hypothetical protein |  | 1.67 |
| PA14_41760 | PA1763 | PA1763 | hypothetical protein |  | -1.95 |
| PA14_42010 | PA1742 | pauD2 | Glutamine amidotransferase class I |  | -1.58 |
| PA14_42250 | PA1725 | pscL | type III export protein PscL |  | 3.52 |
| PA14_42260 | PA1724 | pscK | type III export protein PscK |  | 4.15 |
| PA14_42340 | PA1717 | pscD | type III export protein PscD |  | 3.46 |
| PA14_42360 | PA1715 | pscB | type III export apparatus protein |  | 5.54 |
| PA14_42410 | PA1711 | exsE | ExsE |  | 2.25 |
| PA14_42430 | PA1710 | exsC | ExsC, exoenzyme S synthesis protein C precursor. |  | 3.05 |
| PA14_42530 | PA1700 | pcr2 | Pcr2 |  | 4.30 |
| PA14_42540 | PA1699 | pcr1 | Pcr1 |  | 6.14 |
| PA14_42630 | PA1692 | PA1692 | probable translocation protein in type III secretion |  | 4.10 |
| PA14_43030 | PA1658 | hsiC2 | HsiC2 |  | -1.64 |
| PA14_43040 | PA1657 | hsiB2 | HsiB2 |  | -1.66 |
| PA14_43510 | PA1625 | PA1625 | conserved hypothetical protein |  | 1.80 |
| PA14_43540 | PA1622 | PA1622 | probable hydrolase |  | -1.69 |
| PA14_43550 | PA1621 | PA1621 | probable hydrolase |  | -1.69 |
| PA14_43640 | PA1614 | gpsA | glycerol-3-phosphate dehydrogenase, biosynthetic |  | 1.89 |
| PA14_43670 | PA1611 | PA1611 | hybrid sensor kinase |  | 1.81 |
| PA14_43730 | PA1606 | PA1606 | hypothetical protein |  | 3.98 |
| PA14_43740 | PA1605 | PA1605 | hypothetical protein |  | 2.78 |
| PA14_43840 | PA1597 | PA1597 | hypothetical protein |  | 2.46 |
| PA14_44010 | PA1585 | sucA | 2-oxoglutarate dehydrogenase (E1 subunit) |  | 2.06 |
| PA14_44020 | PA1584 | sdhB | succinate dehydrogenase (B subunit) |  | 1.68 |
| PA14_44030 | PA1583 | sdhA | succinate dehydrogenase (A subunit) |  | 2.00 |
| PA14_44050 | PA1582 | sdhD | succinate dehydrogenase (D subunit) |  | 2.23 |
| PA14_44210 | PA1567 | PA1567 | conserved hypothetical protein |  | -2.21 |
| PA14_44230 | - | - | - |  | -5.73 |
| PA14_44290 | PA1562 | acnA | aconitate hydratase 1 |  | 2.07 |
| PA14_44640 | PA1531 | PA1531 | hypothetical protein |  | -1.57 |
| PA14_44770 | PA1521 | PA1521 | probable guanine deaminase |  | -1.61 |
| PA14_44800 | PA1519 | PA1519 | probable transporter |  | -2.08 |
| PA14_44890 | PA0263 | hcpC | secreted protein Hcp |  | -2.26 |
| PA14_44890 | PA1512 | hcpA | secreted protein Hcp |  | -2.26 |
| PA14_44890 | PA5267 | hcpB | secreted protein Hcp |  | -2.26 |
| PA14_44950 | PA1507 | PA1507 | probable transporter |  | 2.01 |
| PA14_44980 | PA1504 | PA1504 | probable transcriptional regulator |  | 1.57 |
| PA14_45100 | PA1494 | muiA | mucoidy inhibitor gene A |  | 3.99 |
| PA14_45260 | - | - | - |  | 1.95 |
| PA14_46070 | PA1421 | gbuA | guanidinobutyrase |  | -1.85 |
| PA14_46110 | PA1418 | PA1418 | probable sodium:solute symport protein |  | -2.58 |
| PA14_46230 | PA1409 | aphA | acetylpolyamine aminohydrolase |  | 1.86 |
| PA14_46240 | PA1408 | PA1408 | hypothetical protein |  | 1.92 |
| PA14_46260 | PA1406 | PA1406 | hypothetical protein |  | -1.52 |
| PA14_46510 | - | - | - |  | -1.53 |
| PA14_46520 | - | - | - |  | -1.56 |
| PA14_46530 | - | - | - |  | -1.65 |
| PA14_46860 | PA1346 | PA1346 | hypothetical protein |  | 1.84 |
| PA14_47120 | PA1324 | PA1324 | hypothetical protein |  | 4.77 |
| PA14_47130 | PA1323 | PA1323 | hypothetical protein |  | 3.64 |
| PA14_47450 | PA1295 | PA1295 | conserved hypothetical protein |  | 1.96 |
| PA14_47460 | PA1294 | rnd | ribonuclease D |  | 2.19 |
| PA14_47530 | PA1289 | PA1289 | hypothetical protein |  | -1.71 |
| PA14_47840 | PA1268 | lhpA | Hydroxyproline 2-epimerase, LhpA |  | -2.88 |
| PA14_47850 | PA1267 | lhpB | D-hydroxyproline dehydrogenase beta-subunit, LphB |  | -3.88 |
| PA14_47960 | PA1256 | lhpO | ABC transporter ATP-binding protein, LhpO |  | -6.15 |
| PA14_48160 | PA1243 | PA1243 | probable sensor/response regulator hybrid |  | 3.59 |
| PA14_48170 | PA1242 | sprP | SprP |  | 3.46 |
| PA14_49160 | PA1181 | PA1181 | conserved hypothetical protein |  | -1.68 |
| PA14_49200 | PA1178 | oprH | PhoP/Q and low Mg2+ inducible outer membrane protein H1 precursor |  | 1.60 |
| PA14_49210 | PA1177 | napE | periplasmic nitrate reductase protein NapE |  | -2.27 |
| PA14_49230 | PA1175 | napD | NapD protein of periplasmic nitrate reductase |  | -1.64 |
| PA14_49250 | PA1174 | napA | periplasmic nitrate reductase protein NapA |  | -1.60 |
| PA14_49270 | PA1172 | napC | cytochrome c-type protein NapC |  | -1.59 |
| PA14_49290 | PA1170 | PA1170 | conserved hypothetical protein |  | 1.85 |
| PA14_49360 | PA1163 | ndvB | NdvB |  | -1.56 |
| PA14_49940 | PA1115 | PA1115 | hypothetical protein |  | 2.12 |
| PA14_49960 | PA1114 | PA1114 | hypothetical protein |  | 2.54 |
| PA14_50010 | PA1112 | PA1112 | conserved hypothetical protein |  | 1.88 |
| PA14_50020 | PA1111 | PA1111 | hypothetical protein |  | 2.21 |
| PA14_50310 | PA1090 | PA1090 | hypothetical protein |  | -1.56 |
| PA14_50480 | PA1077 | flgB | flagellar basal-body rod protein FlgB |  | -1.53 |
| PA14_50870 | PA1042 | PA1042 | conserved hypothetical protein |  | -2.04 |
| PA14_51050 | PA1027 | amaB | delta1-Piperideine-6-carboxylate dehydrogenase |  | -2.78 |
| PA14_51490 | PA0990 | PA0990 | conserved hypothetical protein |  | 3.19 |
| PA14_51510 | PA0988 | PA0988 | hypothetical protein |  | 1.64 |
| PA14_51740 | PA0970 | tolR | TolR protein |  | 1.52 |
| PA14_51810 | PA0964 | pmpR | pqsR-mediated PQS regulator, PmpR |  | 1.52 |
| PA14_51820 | PA0963 | aspS | aspartyl-tRNA synthetase |  | 1.53 |
| PA14_52230 | PA0931 | pirA | ferric enterobactin receptor PirA |  | 1.92 |
| PA14_52910 | PA0878 | PA0878 | hypothetical protein |  | -2.31 |
| PA14_53220 | PA0854 | fumC2 | fumarate hydratase |  | 1.85 |
| PA14_53260 | PA0851 | PA0851 | hypothetical protein |  | -1.56 |
| PA14_53370 | PA0843 | plcR | phospholipase accessory protein PlcR precursor |  | 2.74 |
| PA14_53410 | PA0839 | PA0839 | probable transcriptional regulator |  | -2.14 |
| PA14_53420 | PA0838 | PA0838 | probable glutathione peroxidase |  | -1.76 |
| PA14_53520 | PA0831 | oruR | transcriptional regulator OruR |  | 1.53 |
| PA14_53800 | PA0809 | PA0809 | probable transporter |  | -2.33 |
| PA14_53820 | PA0807 | ampDh3 | AmpDh3 |  | 2.03 |
| PA14_54240 | PA0776 | PA0776 | hypothetical protein |  | -1.79 |
| PA14_54390 | PA0766 | mucD | serine protease MucD precursor |  | 2.40 |
| PA14_54410 | PA0764 | mucB | negative regulator for alginate biosynthesis MucB |  | 3.02 |
| PA14_54420 | PA0763 | mucA | anti-sigma factor MucA |  | 2.77 |
| PA14_54430 | PA0762 | algU | sigma factor AlgU |  | 2.78 |
| PA14_54730 | PA0738 | PA0738 | conserved hypothetical protein |  | 2.84 |
| PA14_54740 | PA0737 | PA0737 | hypothetical protein |  | 2.41 |
| PA14_54750 | - | - | - |  | 1.86 |
| PA14_55000 | - | - | - |  | -3.04 |
| PA14_55220 | PA0703 | PA0703 | probable major facilitator superfamily (MFS) transporter |  | -1.65 |
| PA14_55780 | PA4293 | pprA | two-component sensor PprA |  | -1.94 |
| PA14_55820 | PA4297 | tadG | TadG |  | -2.05 |
| PA14_55860 | PA4300 | tadC | TadC |  | -2.72 |
| PA14_56030 | PA4311 | PA4311 | conserved hypothetical protein |  | 1.65 |
| PA14_56050 | PA4313 | PA4313 | hypothetical protein |  | 1.58 |
| PA14_56220 | PA4328 | PA4328 | hypothetical protein |  | -1.52 |
| PA14_56380 | PA4337 | PA4337 | hypothetical protein |  | 1.71 |
| PA14_56390 | PA4338 | PA4338 | hypothetical protein |  | 2.26 |
| PA14_56480 | PA4344 | PA4344 | probable hydrolase |  | 2.02 |
| PA14_56510 | PA4345 | PA4345 | hypothetical protein |  | 2.85 |
| PA14_56660 | PA4356 | xenB | xenobiotic reductase |  | 2.20 |
| PA14_56750 | PA4364 | PA4364 | hypothetical protein |  | -3.22 |
| PA14_56780 | PA4366 | sodB | superoxide dismutase |  | 2.21 |
| PA14_57650 | PA4438 | PA4438 | conserved hypothetical protein |  | 1.53 |
| PA14_57820 | PA4451 | PA4451 | conserved hypothetical protein |  | 1.68 |
| PA14_57990 | PA4467 | PA4467 | hypothetical protein |  | -2.45 |
| PA14_58050 | PA4472 | pmbA | PmbA protein |  | -1.63 |
| PA14_58210 | PA4485 | PA4485 | conserved hypothetical protein |  | 1.91 |
| PA14_58330 | PA4495 | PA4495 | hypothetical protein |  | 2.61 |
| PA14_58375 | PA4498 | mdpA | metallo-dipeptidase aeruginosa, MdpA |  | -1.62 |
| PA14_58580 | PA4515 | PA4515 | conserved hypothetical protein |  | 2.32 |
| PA14_58600 | PA4516 | PA4516 | hypothetical protein |  | 1.74 |
| PA14_58800 | PA4531 | PA4531 | hypothetical protein |  | 1.97 |
| PA14_58820 | PA4533 | PA4533 | hypothetical protein |  | 2.13 |
| PA14_58830 | PA4534 | PA4534 | hypothetical protein |  | 2.12 |
| PA14_59180 | - | - | - |  | 1.82 |
| PA14_59560 | - | - | - |  | 2.31 |
| PA14_60520 | PA4573 | PA4573 | hypothetical protein |  | -1.98 |
| PA14_60970 | PA4608 | PA4608 | hypothetical protein |  | -1.57 |
| PA14_61270 | PA4630 | PA4630 | hypothetical protein |  | -1.88 |
| PA14_61400 | PA4640 | mqoB | malate:quinone oxidoreductase |  | 1.87 |
| PA14_61410 | - | - | - |  | -1.55 |
| PA14_61580 | PA4655 | hemH | ferrochelatase |  | 1.65 |
| PA14_61650 | PA4661 | pagL | Lipid A 3-O-deacylase |  | 2.41 |
| PA14_61770 | PA4670 | prs | ribose-phosphate pyrophosphokinase |  | 1.57 |
| PA14_61910 | PA4680 | PA4680 | hypothetical protein |  | -1.94 |
| PA14_61960 | PA4684 | PA4684 | hypothetical protein |  | 1.53 |
| PA14_62260 | PA4704 | cbpA | cAMP-binding protein A |  | -1.76 |
| PA14_62680 | PA4738 | PA4738 | conserved hypothetical protein |  | 2.79 |
| PA14_62690 | PA4739 | PA4739 | conserved hypothetical protein |  | 2.98 |
| PA14_62780 | PA4746 | PA4746 | conserved hypothetical protein |  | 1.62 |
| PA14_63250 | PA4785 | PA4785 | probable acyl-CoA thiolase |  | 2.10 |
| PA14_63270 | PA4786 | PA4786 | probable short-chain dehydrogenase |  | 2.32 |
| PA14_63650 | PA4815 | PA4815 | hypothetical protein |  | 2.38 |
| PA14_64050 | PA4843 | gcbA | GcbA |  | -1.61 |
| PA14_64080 | PA4845 | dipZ | thiol:disulfide interchange protein DipZ |  | 1.54 |
| PA14_64430 | - | - | - |  | 1.99 |
| PA14_64490 | PA4877 | PA4877 | hypothetical protein |  | 3.11 |
| PA14_64500 | PA4878 | brlR | BrlR |  | -1.93 |
| PA14_65720 | PA4972 | PA4972 | hypothetical protein |  | 1.72 |
| PA14_66630 | PA5041 | pilP | type 4 fimbrial biogenesis protein PilP |  | -1.61 |
| PA14_66640 | PA5042 | pilO | type 4 fimbrial biogenesis protein PilO |  | -1.65 |
| PA14_66650 | PA5043 | pilN | type 4 fimbrial biogenesis protein PilN |  | -1.72 |
| PA14_67440 | PA5106 | PA5106 | conserved hypothetical protein |  | 1.55 |
| PA14_67450 | PA5107 | blc | outer membrane lipoprotein Blc |  | 1.51 |
| PA14_67490 | PA5110 | fbp | fructose-1,6-bisphosphatase |  | 1.83 |
| PA14_67500 | PA5111 | gloA3 | lactoylglutathione lyase |  | 3.40 |
| PA14_67600 | PA5119 | glnA | glutamine synthetase |  | -1.98 |
| PA14_67680 | PA5125 | ntrC | two-component response regulator NtrC |  | -241 |
| PA14_68120 | PA5158 | PA5158 | probable outer membrane protein precursor |  | 1.52 |
| PA14_68300 | PA5170 | arcD | arginine/ornithine antiporter |  | -1.59 |
| PA14_68330 | PA5171 | arcA | arginine deiminase |  | -1.62 |
| PA14_68350 | PA5173 | arcC | carbamate kinase |  | -1.58 |
| PA14_68430 | PA5180 | PA5180 | conserved hypothetical protein |  | -2.02 |
| PA14_68450 | - | - | - |  | 1.78 |
| PA14_68460 | - | - | - |  | 2.27 |
| PA14_68660 | PA5197 | rimK | ribosomal protein S6 modification protein |  | 1.57 |
| PA14_68810 | PA5209 | PA5209 | hypothetical protein |  | 2.24 |
| PA14_68840 | PA5212 | PA5212 | hypothetical protein |  | 3.03 |
| PA14_68850 | PA5213 | gcvP1 | glycine cleavage system protein P1 |  | -1.61 |
| PA14_69030 | PA5227 | PA5227 | conserved hypothetical protein |  | 1.71 |
| PA14_69260 | PA5245 | PA5245 | conserved hypothetical protein |  | 1.78 |
| PA14_69270 | PA5246 | PA5246 | conserved hypothetical protein |  | 2.37 |
| PA14_69600 | PA5271 | PA5271 | hypothetical protein |  | -1.59 |
| PA14_69850 | PA5291 | betT2 | BetT2 |  | 2.18 |
| PA14_69925 | PA5297 | poxB | pyruvate dehydrogenase (cytochrome) |  | 4.67 |
| PA14_69950 | PA5299 | PA5299 | hypothetical protein |  | 1.74 |
| PA14_70040 | PA5304 | dadA | D-amino acid dehydrogenase, small subunit |  | -1.56 |
| PA14_70170 | PA5314 | PA5314 | hypothetical protein |  | -1.69 |
| PA14_70270 | PA5322 | algC | phosphomannomutase AlgC |  | 1.90 |
| PA14_70280 | PA5323 | argB | acetylglutamate kinase |  | 1.59 |
| PA14_70740 | PA5359 | PA5359 | hypothetical protein |  | -1.74 |
| PA14_71310 | PA5401 | PA5401 | hypothetical protein |  | -3.54 |
| PA14_71400 | - | - | - |  | -2.38 |
| PA14_71490 | PA5417 | soxD | sarcosine oxidase delta subunit |  | -4.76 |
| PA14_71510 | PA5419 | soxG | sarcosine oxidase gamma subunit |  | -4.19 |
| PA14_71560 | PA5421 | fdhA | glutathione-independent formaldehyde dehydrogenase |  | -1.79 |
| PA14_71570 | PA5422 | PA5422 | hypothetical protein |  | 2.14 |
| PA14_71890 | PA5445 | PA5445 | probable coenzyme A transferase |  | 3.38 |
| PA14_71960 | PA5451 | wzm | membrane subunit of A-band LPS efflux transporter |  | -1.69 |
| PA14_72060 | PA5460 | PA5460 | hypothetical protein |  | -2.33 |
| PA14_72090 | PA5463 | PA5463 | hypothetical protein |  | 1.89 |
| PA14_72110 | PA5464 | PA5464 | hypothetical protein |  | 1.69 |
| PA14_72300 | PA5477 | PA5477 | hypothetical protein |  | 1.83 |
| PA14_72380 | PA5483 | algB | two-component response regulator AlgB |  | 3.71 |
| PA14_72450 | PA5489 | dsbA | thiol:disulfide interchange protein DsbA |  | 1.85 |
| PA14_72760 | PA5514 | PA5514 | probable beta-lactamase |  | 2.26 |
| PA14_72840 | PA5521 | PA5521 | probable short-chain dehydrogenase |  | 2.10 |
| PA14_72920 | PA5527 | PA5527 | hypothetical protein |  | -1.82 |
